# Bioinspired Photoluminescent “Spider Web” as Ultrafast and Ultrasensitive Airflow–Acoustic Bimodal Sensor for Human–Computer Interaction and Intelligent Recognition

**DOI:** 10.1021/acscentsci.4c01182

**Published:** 2024-09-26

**Authors:** Kai Zhu, Bing Yan

**Affiliations:** † School of Chemical Science and Engineering, Tongji University, Siping Road 1239, Shanghai 200092, China

## Abstract

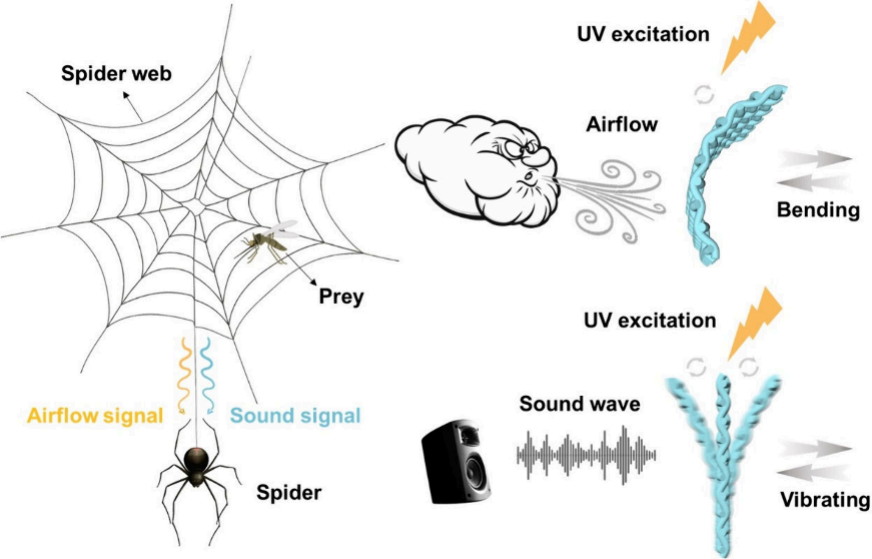

Nature provides massive biomimetic design inspiration for constructing structural materials with desired performances. Spider webs can perceive vibrations generated by airflow and acoustic waves from prey and transfer the corresponding information to spiders. Herein, by mimicking the perception capability and structure of a spider web, an ultrafast and ultrasensitive airflow–acoustic bimodal sensor (HOF-TCPB@SF) is developed based on the postfunctionalization of hydrogen-bonded organic framework (HOF-TCPB) on silk film (SF) through hydrogen bonds. The “spider web-like” HOF-TCPB@SF possesses light weight and high elasticity, endowing this airflow sensor with superior properties including an ultralow detection limit (DL, 0.0076 m s^–1^), and excellent repeatability (480 cycles). As an acoustic sensor, HOF-TCPB@SF exhibits ultrahigh sensitivity (105140.77 cps Pa^–1^ cm^–2^) and ultralow DL (0.2980 dB), with the greatest response frequency of 375 Hz and the ability to identify multiple sounds. Moreover, both airflow and acoustic sensing processes show an ultrafast response speed (40 ms) and multiangle recognition response (0–180°). The perception mechanisms of airflow and acoustic stimuli are analyzed through finite element simulation. This bimodal sensor also achieves real-time airflow monitoring, speech recognition, and airflow–acoustic interoperability based on human–computer interaction, which holds great promise for applications in health care, tunnel engineering, weather forecasting, and intelligent textiles.

## Abstract 2

A photoluminescent “spider web-like” ultrafast and ultrasensitive airflow−acoustic bimodal bioinspired sensor based on HOF-TCPB@SF was fabricated that can realize human−computer interaction and intelligent recognition, which exhibits significant potential in human health detection, weather forecasting, tunnel engineering, warning reconnaissance, intelligent textiles, and smart bionics.

## Introduction

Nature has developed ever-present optimal structures with remarkable characteristics and swift stimuli-responsive capabilities over millions of years of evolution, which provides precious sources of inspiration for the construction of next-generation functional materials.^1−4^ Bionics is aimed to leverage the precision-engineered designs observed in nature, including the structure, function, and adaptability of organisms, to develop innovative methodologies and technologies.^5−7^ Bioinspired sensor systems, compared with traditional sensing systems, employ existing technologies and processes to simulate natural structures and materials, bringing about performance that is comparable to natural sensing systems.^8^ In nature, spiders are one of the oldest species. Spider web, composed of silk threads secreted by spiders, is an interwoven network with high tensile strength, toughness, stability, and elasticity and has distinct optical and biocompatible properties.^9−11^ As is well-known, spiders can sensitively perceive vibration differences transmitted by the spider webs, thus accurately orienting their preys.^12^ According to recent research, a spider web can capture vibrations caused by slight airflow and acoustic waves originating from insects flapping their wings, vibrating the eardrum, etc., and further transmit the information on the surroundings to the spider in a noncontact manner, which resembles outsourcing hearing to inform spiders in advance of potential preys in their vicinity, thereby capturing food.^13−16^ The remote sensing ability of spiders through spider webs to external airflow and sound signals provides unique features for the design of a novel airflow–acoustic bimodal detector for precise dynamics measurement and manipulation.

In recent years, flexible biomimetic sensors have developed explosively, and so far, some airflow or acoustic sensors have been reported.^17−24^ Among them, there are only sporadic reports of spider-inspired airflow or acoustic sensors. For example, an ultrasensitive and flexible all-textile airflow sensor based on fabric with in situ grown carbon nanotubes has been developed by mimicking the spider’s fluff.^25^ A freely suspended nanofiber mesh similar to spider silk shows broad sensitivity bandwidth at hearing-safe sound pressure levels.^26^ However, compared with extensive studies on other response types of sensors such as flexible pressure and temperature sensors, research on flexible biomimetic airflow and sound sensors is far behind.^27−30^ Moreover, previous research on detecting airflow or sound mainly focused on electronic devices, while the studies on optical airflow or acoustic sensors, especially optical airflow–acoustic bimodal sensors, have not been reported. The optical airflow–acoustic bimodal sensors can be applied in environmental monitoring, weather forecasting, tunnel engineering, speech recognition, and healthcare fields, possessing the merits of high accuracy, high stability, high sensitivity, and high efficiency. Therefore, it is highly desirable to develop optical airflow–acoustic bimodal sensors.

As novel porous crystalline materials, hydrogen-bonded organic frameworks (HOFs), self-assembled from organic building blocks through intermolecular hydrogen bonding interactions and π–π stacking, have attracted extensive attention due to mild synthesis conditions and solution processability.^31−34^ Since the large π-conjugated systems of aromatic moieties are the common building blocks, most HOFs can produce strong fluorescence emission.^35,36^ And the strong hydrogen bonding interactions and dense π–π stacking in HOFs are conducive to restricting nonradiative transitions, thus further enhancing fluorescence.^37^ The intense fluorescence can serve as the detection indicator for the physical signal (airflow, sound, etc.) sensing of HOFs. Moreover, the presence of unpaired and residual hydrogen donors and acceptors in the HOF structure enables HOFs to facilely incorporate with exogenous materials through hydrogen bonds.^38^ Silk fiber is one of the most highly engineered natural fibers, which is widely used in the textile industry and scientific community attributed to its low cost, high elasticity, light weight, great biocompatibility, and outstanding mechanical properties.^39−41^ Silk fiber, spun from the two glands of a silkworm, is composed of two primary filaments called brin, which contains fibroin proteins.^42^ There are massive dissociative amine (−NH_2_) and hydroxyl (−OH) groups in fibroin proteins of silk fibers, which can form X–H···O (X = O, N) hydrogen bonds with carboxyl groups (−COOH) in HOF-TCPB to coat HOF-TCPB onto silk film (SF) for the preparation of HOF-TCPB@SF.^43^ HOF-TCPB@SF combines the advantages of both HOF-TCPB and SF, the network structure of which is similar to that of a spider web, endowing HOF-TCPB@SF with superior performance to detect airflow and sound signals as sensitively as spiders.

**Scheme 1:**
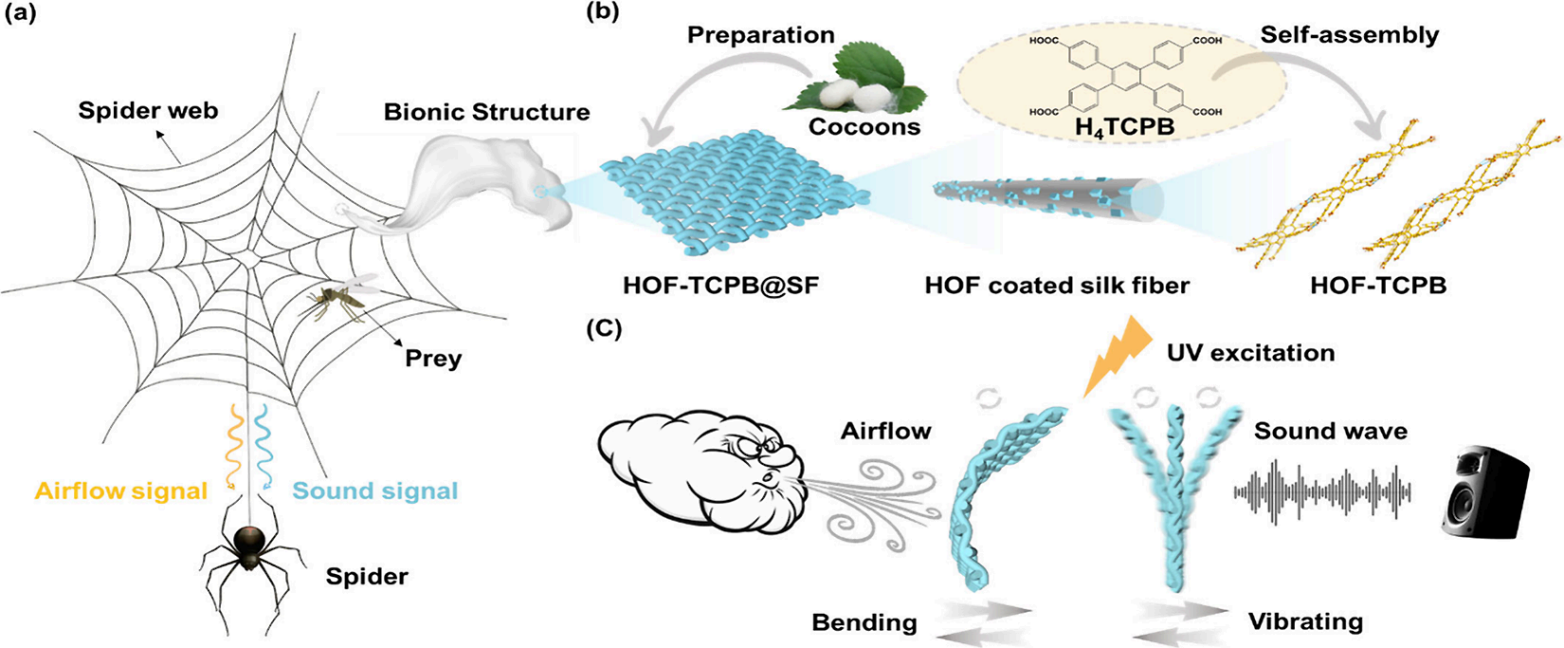
(a) The Process of Spiders Preying on Prey by Perceiving Airflow and Sound Signals through Spider Web, (b) Scheme of the Spider Web Biomimetic Structure and Microscopic Composition of HOF-TCPB@SF, and (c) Scheme of Airflow and Sound Wave Sensing for HOF-TCPB@SF Airflow–Acoustic Bimodal Sensor Based on Bending and Vibrating Response

Enlightened by the network structure and perception capability of the spider web, HOF-TCPB@SF was prepared as an optical airflow–acoustic bimodal sensor based on the assembly of HOF-TCPB and SF via hydrogen bonds (Scheme 1a,b). When external airflow and sound signals are received, the sensor will undergo bending and vibration, respectively, to achieve the sensing of these two signals (Scheme 1c). HOF-TCPB@SF with 0.02 cm thickness displays light weight, high elasticity, and outstanding flexibility and has an ultralow detection limit (DL, 0.0076 m s^–1^), ultrahigh sensitivity (12.48 m^–1^ s), and great repeatability (480 cycles) during airflow sensing procedures within 0.04–1.6 m s^–1^. Besides, HOF-TCPB@SF as an acoustic sensor can monitor broad-band sound in the range of 110–35 dB, with ultrahigh sensitivity (105140.77 cps Pa^–1^ cm^–2^), ultralow DL (0.2980 dB), great precision (relative standard deviation, RSD < 3.5%), and excellent repeatability (330 cycles). The airflow and acoustic sensing processes both exhibit ultrafast response speed (40 ms) and multiangle recognition response (0–180°). Based on finite element simulation analysis, the sensing mechanism of HOF-TCPB@SF for airflow sensing can be attributed to the bending of the sensor, while that for sound can be due to its vibration. Combined with human–computer interaction technology, this bimodal sensor can not only realize Morse code-assisted respiratory information expression, real-time airflow monitoring, and speech recognition with high precision but also implement the airflow–acoustic interoperability. This research presents insights into designing and preparing high-performance airflow–acoustic sensors, enabling significant potential in health care, tunnel engineering, weather forecasting, warning reconnaissance, intelligent textiles, intelligent bionics, and flexible optical devices.

## Experimental Section

### Preparation of HOF-TCPB

HOF-TCPB was prepared according to the previous report with some modifications.^44^ 0.1 mmol (55.8 mg) of 1,2,4,5-tetrakis(4-carboxyphenyl)benzene (H_4_TCPB) was dissolved in 7.5 mL of DMF to obtain a clear solution by ultrasound. Subsequently, 9 mol (162 mL) of water was added into the above solution. After stirring for 45 min at room temperature, the mixture was separated via centrifugation at 10000 rpm for 30 min and then was washed continuously with 10 mL of water for two times. Finally, the product could be obtained after annealing at 150 °C for 24 h.

### Preparation of HOF-TCPB@SF as Airflow–Acoustic Bimodal Sensor

The SF with a size of 4.0 × 2.0 × 0.02 cm^3^ was immersed in ethanol with ultrasonic treatment for 10 min to obtain clean SF. The naturally dried SF was immersed into 10 mL of an ethanol solution containing 20 mg of HOF-TCPB powder for 2 h. Then this piece of SF-attached HOF-TCPB was taken out from the solution and was dried at room temperature for 12 h to obtain HOF-TCPB@SF as an airflow–acoustic bimodal sensor.

### Airflow Velocity Testing

According to different velocities, there are two airflow velocity (*v*) testing methods. When the airflow velocity was lower than 0.4 m s^–1^, it was calculated using the formula *v* = *Q*/*S*, in which *v* is the airflow velocity, *Q* is the flow rate of nitrogen controlled by the flowmeter, and *S* is the pipeline cross-sectional area (*S* = π*r*^2^). As the airflow velocity exceeded 0.4 m s^–1^, it was directly measured with a high-precision anemometer.

### Airflow Sensing Process

A pipeline connecting to a nitrogen (N_2_) cylinder was placed behind the HOF-TCPB@SF airflow sensor, and one side of the sensor was fixed on a clip. N_2_ flowed out of the pipeline and blew to the HOF-TCPB@SF airflow sensor at a certain distance, and the variation in the fluorescence intensity at 406 nm of the sensor was measured using an Edinburgh FLS920 spectrophotometer at room temperature. The relative variation in fluorescence intensity (Δ*I*/*I*_0_) was calculated according to the measurement results, in which *I*_0_ represents the initial fluorescence intensity without airflow, *I* represents the fluorescence intensity when airflow is applied to the sensor, and Δ*I* is the fluorescence intensity variation *I* – *I*_0_.

### Finite Element Simulation of SF in Airflow Field

The analysis of the airflow sensing process was achieved based on the multiphysics field coupling—“fluid-structure interaction”. Finite element simulation (FES) of HOF-TCPB@SF airflow sensor in an airflow field has been simulated by COMSOL 6.2 software. The 3D model including airflow field and HOF-TCPB@SF airflow sensor were constructed. The size of the airflow field was 18 × 15 × 10 cm^3^, and the size of the HOF-TCPB@SF airflow sensor was 4.0 × 2.0 × 0.02 cm^3^. The physical fields contain “laminar flow”, “solid mechanics”, and “ fluid-structure interaction”. The temperature and pressure were set as 293.15 K and 1 atm, respectively. The airflow blowing angle (θ) was 90°. The output results contained the bending changes of the film with the variation in airflow velocity (0–1.6 m s^–1^).

### SPL and Frequency Testing

The APP software “dB Meter” in a phone was used to monitor the SPLs, and the APP “Audio generator” was applied to generate specific sounds of different frequencies (*f*).

### Acoustic Sensing Process

A loudspeaker connected to a mobile phone or computer was placed behind the HOF-TCPB@SF acoustic sensor, and one side of the sensor was fixed on a clip. When a series of sounds with different SPLs (110, 90, 70, 60, 50, 40, 35 dB) were emitted toward the acoustic sensor, the kinetic curve was measured by monitoring the 406 nm emission peak of the acoustic sensor. The variation in fluorescence intensity (Δ*I*) at 406 nm induced by the sounds with different SPLs was measured using an Edinburgh FLS920 spectrophotometer.

The measurement of different frequencies was similar to SPL measurements except that the loudspeaker emitted sounds with different frequencies.

### Finite Element Simulation of SF in Sound Field

The analysis of the sound sensing process was achieved based on the Multiphysics field coupling—“sound-structure interaction”. FES of the HOF-TCPB@SF acoustic sensor in the sound field has been simulated by COMSOL 6.2 software. A 3D model including sound field and HOF-TCPB@SF acoustic sensor was constructed. The size of the sound field was 18 × 15 × 10 cm^3^, and the size of the HOF-TCPB@SF airflow sensor was 4.0 × 2.0 × 0.02 cm^3^. The physical fields contain “solid mechanics”, “pressure acoustics, frequency domain”, and “sound-structure boundary”. The temperature and pressure were set as 293.15 K and 1 atm, respectively. The sound incidence angle (γ) was 90°. The output results contained stress, total sound pressure, total sound pressure isosurfaces, and sound pressure level distribution of the HOF-TCPB@SF acoustic sensor.

### Repeatability Testing of Airflow and Acoustic Sensing

First, with regard to the airflow sensing process, we operated the airflow valve to open and close the airflow repeatedly to test the repeatability of the sensor for airflow sensing. Furthermore, for the sound sensing process, the same sound was played repeatedly to test the repeatability of the sensor for sound sensing.

### DFT Calculation

The molecular structure optimization was carried out by using the Gaussian 16W package based on the b3lyp/6-31g basis group and method. The HOMO and LUMO energy levels of the H_4_TCPB molecule were acquired by inputting the chk. file of the optimized structure into the GaussView 6.0 package.

### Construction of Back-Propagation Neural Network

BPNN1, BPNN2, BPNN3, and BPNN4 were constructed by using the “Maishishenjingwangluo” software. All supporting data of BPNN1–4 are given in Tables S4–S19.

## Results and Discussion

### Design and Characterization of HOF-TCPB@SF as Airflow–Acoustic Bimodal Sensor

**Figure 1:**
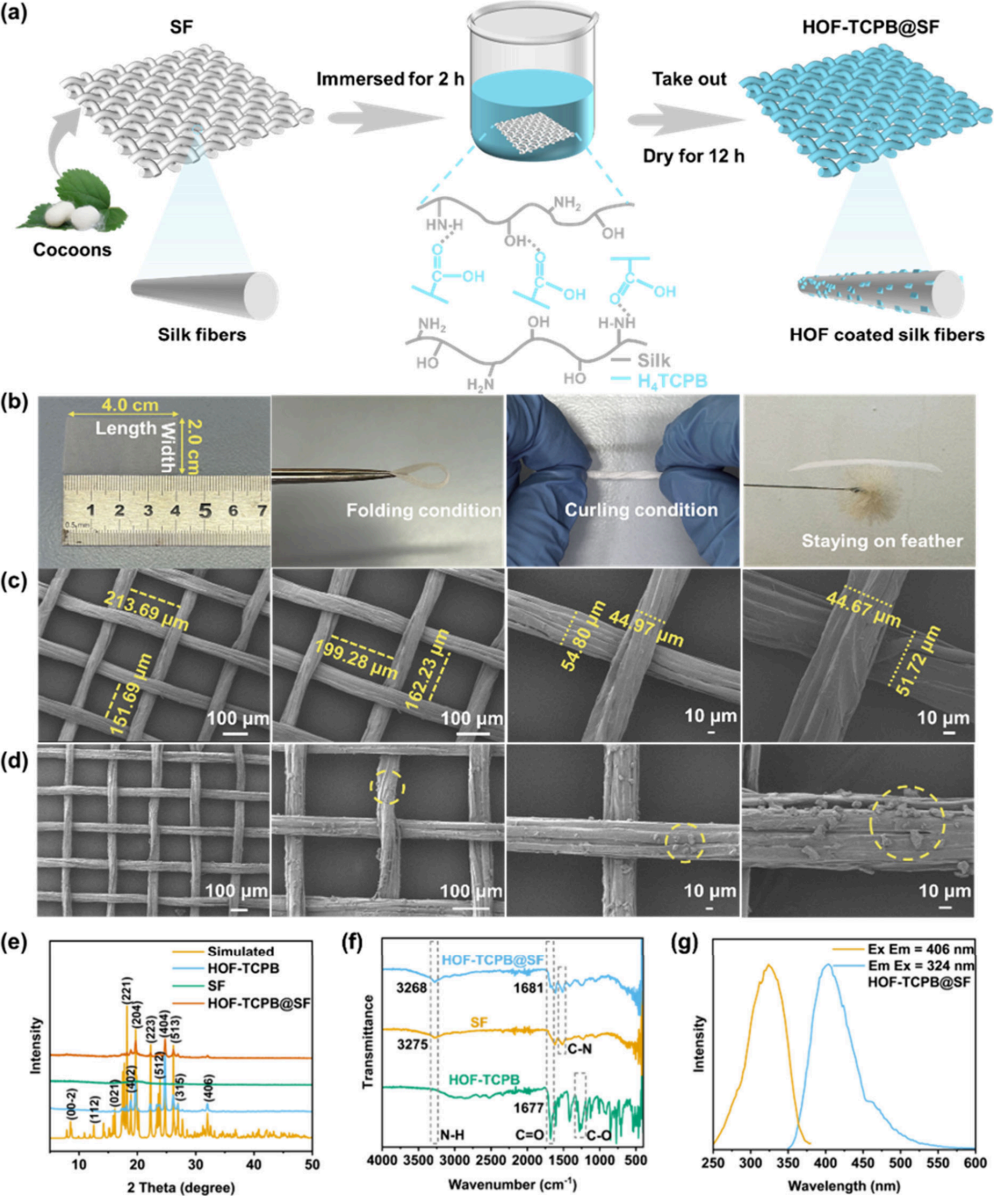
(a) Scheme of the preparation of HOF-TCPB@SF. (b) Pictures of HOF-TCPB@SF with a size of 4.0 × 2.0 × 0.02 cm^3^ under natural light, with the ability of folding, curling, and staying on a feather. (c) SEM pictures of SF. (d) SEM pictures of HOF-TCPB@SF. (e) PXRD patterns of HOF-TCPB, SF, and HOF-TCPB@SF. (f) FT-IR spectra of HOF-TCPB, SF, and HOF-TCPB@SF. (g) Excitation and emission spectra of HOF-TCPB@SF.

The HOF-TCPB@SF airflow–acoustic bimodal sensor was prepared by using an efficient one-step dip-coating approach. HOF-TCPB is constructed based on the self-assembly of 1,2,4,5-tetrakis(4-carboxyphenyl)benzene (H_4_TCPB) through duplex H-bonds (Figure S1), with strong blue fluorescence.^44^ Herein, HOF-TCPB was chosen as a luminescent substrate rather than other crystalline materials such as metal organic frameworks (MOFs) or covalent organic frameworks (COFs) owing to these advantages. (i) Compared with MOFs and COFs, there are plentiful terminal carboxylic groups on the X-shaped organic building blocks of HOF-TCPB, facilitating HOF-TCPB to bond with SF film through hydrogen bonds. (ii) Benefiting from the metal-free property, HOF-TCPB shows better biocompatibility and lower toxicity compared with MOFs, which has lower harmfulness during the use process. (iii) HOF-TCPB possesses stronger luminescence than most COFs. Silk fiber is a soft, smooth texture, and lightweight (density ∼25.83 g m^–3^) natural protein-based biomaterial, which possesses the appealing properties of high flexibility, high biocompatibility, high elasticity, and light weight. Due to the high density of −OH and – NH_2_ groups, silk fiber can integrate with the terminal carboxylic groups of HOF-TCPB via hydrogen bonds to prepare HOF-TCPB@SF. Figure 1a illustrates the fabrication process of the all-textile airflow–acoustic bimodal sensor. Clean SF was completely immersed into an HOF-TCPB ethanol solution for 2 h and then taken out horizontally. After it was dried naturally at room temperature for 12 h, a white HOF-TCPB@SF bimodal sensor with a size of 4.0 × 2.0 × 0.02 cm^3^ (Figure 1b and Figure S2) that can emit brilliant blue fluorescence was obtained. Based on a low load bending test (Figure S3), the bending stiffness of SF was measured to be 9.6618 MPa. Hence, HOF-TCPB@SF combines the strong and stable photoluminescence characteristics of HOF-TCPB with the exceptional flexibility (folding and curling ability), light weight (staying on feather), resilience, and mechanical properties of SF (Figure 1b), endowing it with enormous potential as an airflow–acoustic dual-mode sensor. The representative scanning electron microscopy (SEM) morphology of SF indicates its clearly evident monolayer woven fabric structure similar to that of a spider web, which is formed by the interconnection of silk fibers with a diameter of ∼44.67–54.80 μm (Figure 1c). As shown in Figure 1c, the original silk fibers exist as a clean and flat surface, and the distance between adjacent fibers is ∼151.69–213.69 μm. After SF was soaked in a HOF-TCPB solution and dried, the as-fabricated HOF-TCPB@SF still maintained its original fabric structure consisting of silk fibers (Figure 1d). Meanwhile, large numbers of bulk crystals were uniformly and closely coated on the branch fibers within the skeleton of the SF (Figure 1d). And the crystal morphology on SF fibers is consistent with that of pure HOF-TCPB, proving that HOF-TCPB was attached on SF fibers and the crystal structure remained stable (Figure 1d and Figure S4). Energy dispersive X-ray (EDX) mapping images manifest that there are C, O, and N elements in SF fibers (Figure S5). After HOF-TCPB was decorated on SF, the C and O elements of HOF-TCPB on fiber chains could be scanned out plainly by EDX mapping (Figure S6). Additionally, the increasing weight percentages of C (57.91%) and O (28.97%) elements in HOF-TCPB@SF measured by EDX compared with those of SF (C, 53.79%; O, 28.14%) also indicate the attachment of HOF-TCPB on the SF fiber skeleton (Figure S7 and Table S2). As shown in Figure 1e, the powder X-ray diffraction (PXRD) pattern reveals that the diffraction peaks of HOF-TCPB@SF are in accordance with those of both the prepared HOF-TCPB and the simulated pattern, which demonstrates that the 2-fold interpenetrating framework structures of HOF-TCPB do not change in the HOF-TCPB@SF system. And the PXRD peaks of HOF-TCPB after immersion in methanol, toluene, ethanol, acetonitrile, acetone, dichloromethane, acidic (pH = 3), neutral (pH = 7), and alkaline (pH = 11) aqueous solutions for 24 h match well with the original PXRD patterns, which illustrates that the framework structure of HOF-TCPB exhibits excellent chemical, water, and pH stability (Figure S8). Moreover, the Fourier transform infrared (FT-IR) spectra of HOF-TCPB, SF, and HOF-TCPB@SF were measured to further study the binding interaction between HOF-TCPB and SF. As Figure 1f shows, the FT-IR spectrum of HOF-TCPB@SF contains the characteristic absorption peaks of HOF-TCPB and SF concurrently in the range of 4000–1500 cm^–1^. After HOF-TCPB was attached to the SF framework, the absorption peak at 1677 cm^–1^ attributed to the antisymmetric stretching vibration of C=O bonds (−COO– groups) of HOF-TCPB was red-shifted to 1681 cm^–1^ of HOF-TCPB@SF (Figure 1f), which may be due to the hydrogen bonding interaction between HOF-TCPB and SF. Compared with primordial SF, there was a new peak at 1681 cm^–1^ ascribed to C=O bonds belonging to HOF-TCPB emerged and the peak at 3275 cm^–1^ red-shifted to 3268 cm^–1^ for HOF-TCPB@SF. And simultaneously, the absorption peak of HOF-TCPB@SF in the range of 3100–3600 cm^–1^ was obviously stronger and wider than that of SF (Figure S9), suggesting that massive amounts of X–H···O (X = O, N) hydrogen bonds were formed between HOF-TCPB and SF. The thermogravimetric analysis (TGA) of HOF-TCPB manifests that when the temperature reached 404 °C, the framework of HOF-TCPB started to collapse (Figure S10), which indicates the remarkable thermal stability of HOF-TCPB below 404 °C. In conclusion, SEM, EDX, PXRD, FT-IR, and TGA all strongly confirm the successful fabrication of the HOF-TCPB@SF bimodal sensor.

The luminescence performances of HOF-TCPB and HOF-TCPB@SF were further investigated. As shown in Figure S11, the UV absorption spectrum of HOF-TCPB presents a wide absorption band within 190–400 nm, and correspondingly, the optimal excitation of HOF-TCPB is at 340 nm (Figure S12a), which is located in the scope of a UV spectrum. Under 340 nm excitation, HOF-TCPB can generate a wide fluorescence emission band centered at 406 nm, deriving from the S_1_ → S_0_ transition of HOF-TCPB (Figure S12a). Correspondingly, the emission map of HOF-TCPB also shows that with the excitation varying from 240 to 360 nm, the optimal fluorescence emission peak is located at 406 nm, and the change trend of emission peak intensity is in line with that of excitation intensity, while the excitation peak at 340 nm is the strongest (Figure S12b). In the CIE chromaticity diagram of HOF-TCPB (Figure S12c), it is obvious that the chromatic coordinates attributed to the emission spectrum of HOF-TCPB are located in the blue range (0.1578, 0.0713). Besides, the short and long fluorescence lifetimes (τ_1_ and τ_2_) of the 406 nm peak are 9.02 and 120.06 μs, respectively, and according to the formula
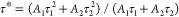
1in which *A*_1_ = 762.44 and *A*_2_ = 24.03, the average decay lifetime (τ*) of HOF-TCPB was calculated to be 41.84 μs (Figure S12d). Furthermore, based on the b3lyp/6-31g density functional theory calculation, the highest occupied molecular orbital and lowest unoccupied molecular orbital of a H_4_TCPB molecule were calculated as −6.3213 and −2.0941 eV, respectively (Figure S13). The photoluminescence quantum yield of HOF-TCPB was measured up to 64.51% (Figure S14), which may be because (i) the H_4_TCPB molecule comprises five benzene rings, the π–π conjugation of which can promote fluorescence emission, (ii) the presence of a rigid planar structure in a H_4_TCPB molecule can enhance fluorescence, and (iii) the hydrogen bonding interactions in the HOF-TCPB framework can effectively suppress the nonradiation energy dissipation resulting from the vibration of H_4_TCPB molecules. After HOF-TCPB was decorated on the SF structure, owing to the hydrogen bonding interactions between HOF-TCPB and SF, the optimal excitation of HOF-TCPB@SF blue-shifted from 340 to 324 nm (Figure 1g). Under excitation at 324 nm, there is also a strong emission band for HOF-TCPB@SF situated at 406 nm (Figure 1g). Compared with the original SF without luminescence, HOF-TCPB@SF can emit visible blue fluorescence under a 310 nm UV lamp (Figure S15). And according to eq 1, the decay lifetime (τ*) of HOF-TCPB@SF at 406 nm was determined as 41.29 μs (Figure S16), which is similar to that of HOF-TCPB (41.84 μs). Further studies on the chemical and temperature stability of luminescence for HOF-TCPB@SF were carried out. As displayed in Figure S17a,b, when HOF-TCPB@SF was exposed to various atmospheres of volatile gases including water, ether, methanol, acetonitrile, cyclohexane, trichloromethane, toluene, and acetone, the fluorescence intensity of HOF-TCPB@SF remained stable, indicating the great chemical and water stability of luminescence. Furthermore, after HOF-TCPB@SF was taken out from a 30–80 °C temperature environment, the emission intensity of HOF-TCPB@SF showed only a slight enhancement (30359–31783 cps), manifesting the good luminescence thermal stability of HOF-TCPB@SF within 30–80 °C (Figure S17c,d). The excellent luminescent performance and outstanding anti-interference ability of HOF-TCPB@SF demonstrate its enormous potential as an optical airflow–acoustic sensor.

### Airflow Sensing and Mechanism

**Figure 2:**
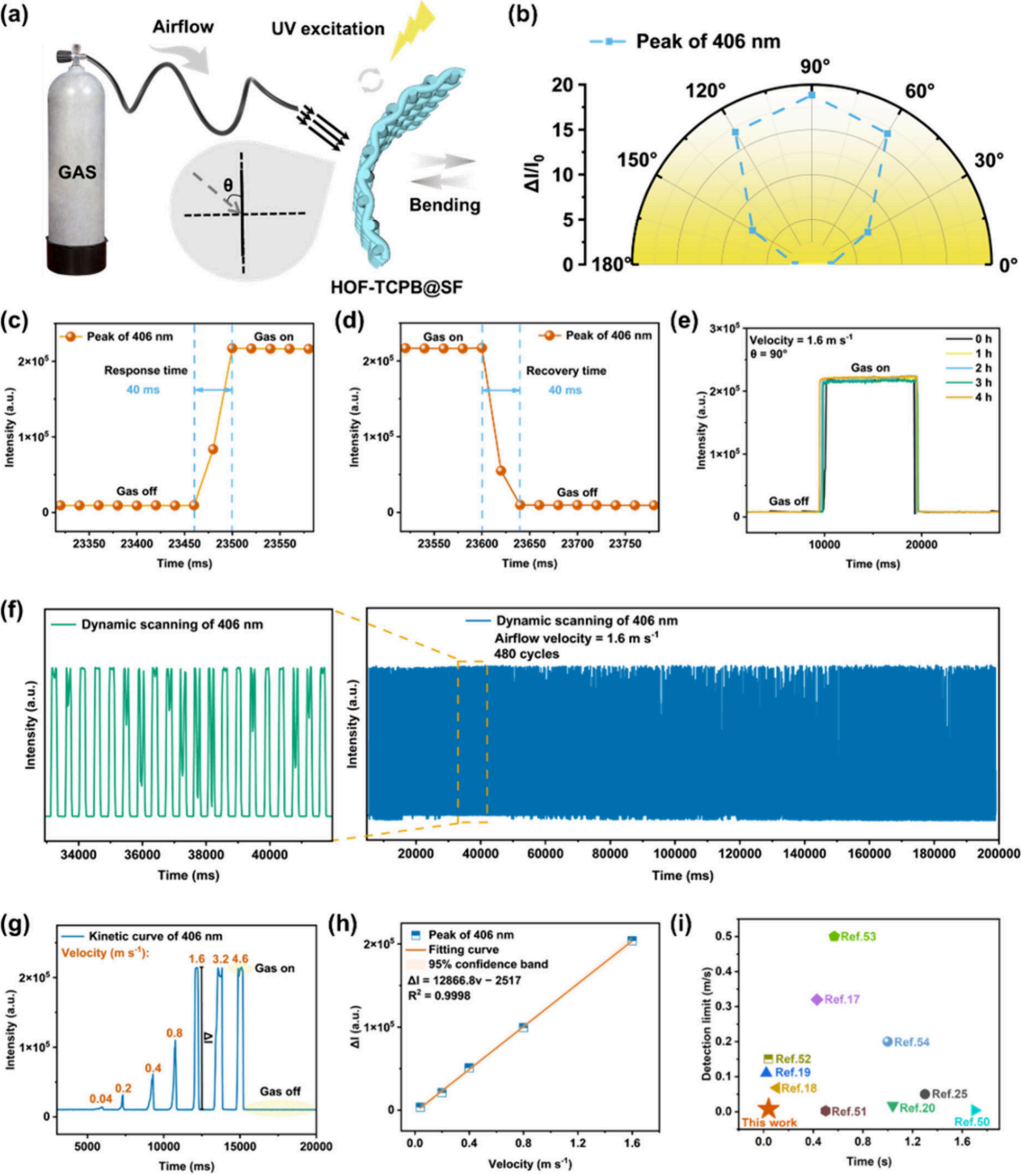
(a) Schematic diagram of the setup for the airflow sensing test, where θ represents the airflow blowing angle. (b) Dependence of Δ*I*/*I*_0_ at 406 nm on different airflow blowing angles θ (0 to 180°). (c) Response time of the HOF-TCPB@SF sensor to airflow. (d) Recovery time of the HOF-TCPB@SF sensor to airflow. (e) Robustness test of the HOF-TCPB@SF airflow sensor under a constant airflow (1.6 m s^–1^) for 4 h. (f) Optical response of the HOF-TCPB@SF airflow sensor under 480 cycles of nitrogen flow loading. The figure on the left is an enlarged view of 20 cycles. (g) Optical response of the HOF-TCPB@SF airflow sensor under a nitrogen flow with different airflow velocities (0.04–4.6 m s^–1^). (h) Linear relationship between the fluorescence intensity variation (Δ*I*) of HOF-TCPB@SF and the airflow velocity. (i) Comparison of the response time and the detection limit of the HOF-TCPB@SF airflow sensor with those of airflow sensors in refs (17−20, 25, and 50−54).

Airflow sensing plays a critical role in many applications, such as the aerospace industry, breath monitoring, and weather forecasting.^45−48^ Based on advantages such as ultralight weight, excellent flexibility, and strong fluorescence emission, the as-prepared “spider web-like” HOF-TCPB@SF optical airflow sensor displays multiangle airflow recognition response, ultrafast response speed, and great robustness. The setup for testing airflow sensing performance is shown in Figure S18, including a nitrogen tank, glass rotameter, and HOF-TCPB@SF-based airflow sensor. The nitrogen tank connected to a gas tube was used to generate and introduce reciprocating airflow with a certain velocity, which could drive HOF-TCPB@SF to bend in turn, resulting in the change in recorded optical signal (Figure 2a and Movie S1). The detection of airflows using an HOF-TCPB@SF-based sensor was realized by measuring its fluorescence intensity variations at 406 nm under a constant bias. Figure 2a illustrates the testing of airflow with various airflow blowing angles (θ) using this airflow sensor. The blowing angle is defined as the angle between the tube and the sensor surface. As shown in Figure 2b, with the angle increasing from 0 to 90°, the fluorescence variation of the sensor gradually increases. On the contrary, when the airflow angle changes from 90° to 180°, the fluorescence variation of the sensor gradually decreases, which may be due to the gradual reduction of the vertical component force of the airflow at the same velocity. It is obvious that 90° is the optimal blowing angle for airflow; hence, the subsequent experiments were conducted at this angle (Figure S19). To study the influence of the distance between the gas outlet and the sensor surface on the fluorescence response, the distance between the pipe outlet and the HOF-TCPB@SF plane was sequentially increased from 2 to 4, 6, 8, and 10 cm. As shown in Figure S20a, with the distance increases, the viscosity of the air can gradually weaken the airflow, resulting in a gradual decrease in fluorescence intensity variation. The relationship between relative fluorescence intensity variation Δ*I*/*I*_0_ and distance (*d*) can be expressed by the equation (Figure S20b)
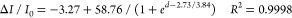
2

The response and recovery performance of the airflow sensor are also investigated in Figure 2c,d. Herein, the response and recovery time are defined as the time from flow on/off to 100% increase/decrease. Based on the kinetic curve of 406 nm, the response time and recovery time of the airflow sensor are both as ultralow as 40 ms, which further demonstrates that the HOF-TCPB@SF-based airflow sensor shows high efficiency to detect airflow. Meanwhile, the response time and recovery time of the airflow sensor are the same, confirming its remarkable rebound performance. The robustness and stability of the HOF-TCPB@SF-based airflow sensor were further assessed. Figure 2e shows that when the HOF-TCPB@SF airflow sensor was placed under a constant airflow (1.6 m s^–1^) for 4 h, similar and steady response curves could be obtained at different times during 4 h, manifesting the exceptional robustness of the fiber structure of the airflow sensor. And the fluorescence intensity at 406 nm remains basically unchanged under a continual airflow (1.6 m s^–1^) within 10–40 s (Figure S21), proving the stability of the airflow sensor under a continuous airflow. Additionally, the HOF-TCPB@SF airflow sensor also behaves great repeatability, ultralow DL, and ultrahigh sensitivity. As shown in Figure 2f, the fluorescence sensing signal reveals no significant signal degradation in the inset over 480 consecutive cycles of airflow on/off, suggesting the excellent repeatability and stability of the HOF-TCPB@SF-based airflow sensor. During the repeatability testing process, some of the top region of the curve can be split, which probably can be attributed to the bouncing of the airflow causing multiple blowing of HOF-TCPB@SF surface. The fluorescence intensity variation at 406 nm in response to airflow at different velocities was explored. Figure 2g displays the plot of the change in fluorescence intensity versus airflow velocity. With the airflow velocity increasing from 0.04 to 1.6 m s^–1^, the fluorescence intensity variation of the sensor increased gradually. When the airflow velocity continues to increase, the fluorescence intensity changes remain basically constant at 3.2 and 4.6 m s^–1^. Within the range 0.04–1.6 m s^–1^ for airflow velocity, a linear relationship can be determined between fluorescence intensity variation (Δ*I*) and airflow velocity (*v*) with the equation (Figure 2h)
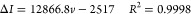
3

According to the criterion of International Union of Pure and Applied Chemistry
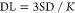
4where *K* (12866.8) is the slope of the fitting curve (Figure 2h) and SD is the standard deviation of 10 times of the initial emission intensity (*I*_0_), the DL was calculated to be as ultralow as 0.0076 m s^–1^ for airflow monitoring. Moreover, the fluorescence response signal of the HOF-TCPB@SF sensor before and after bending shows no apparent variation, revealing the superb flexibility of the airflow sensor (Figure S22). And based on the equations
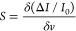
5
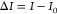
6in which *I*_0_ and *I* represent the initial fluorescence intensity without airflow and the fluorescence intensity when airflow is applied, respectively, Δ*I* is the fluorescence intensity variation, and ν is the airflow velocity, the sensitivity of this airflow sensor can be obtained to be as high as 12.48 m^–1^ s. To study the precision of the airflow sensor, taking airflow velocity = 1.6 m s^–1^ as an example, we investigate the relative fluorescence intensity changes (Δ*I*/*I*_0_) over ten cycles (Figure S23). The results showed that Δ*I*/*I*_0_ remained stable during 10 cycles of measurement with the calculated RSD = 0.48%, demonstrating the high precision and reliability of the airflow sensor. Moreover, as a common concern, the effects of temperature on airflow measurement were measured in this work. As shown in Figure S24, with the temperature gradually rising from room temperature (22 °C) to 100 °C, there is only a slight increase in Δ*I*/*I*_0_ (27.45–29.23), indicating that the airflow sensing process is less affected by temperature.

Figure 2i presents the comparison of the sensing performance between our HOF-TCPB@SF airflow sensor and those of other airflow sensors reported in the literature. It is worth noting that owing to the extreme light weight and excellent flexibility of the “spider web-like” structure, our sensor realizes an ultrafast response (40 ms) and ultralow detection limit (0.0076 m s^–1^) simultaneously, which is superior to the vast majority of reported airflow sensors (Table S2).^16−20,25,49−59^ Although a few sensors possess a relatively fast response or low detection limit, most of them cannot have both a fast response time and low detection limit, which severely limits their use. For instance, a miniaturized thermoelectric device that can distinguish slow airflow was reported, while its response time required at least 1.7 s.^55^ A single ultralong silicon nanowire airflow sensor with a high response speed equivalent to that of our sensor was fabricated. However, its detection limit was measured as 0.15 m s^–1^.^57^ Therefore, HOF-TCPB@SF is an extremely excellent candidate as an airflow sensor by comparison analysis.

**Figure 3:**
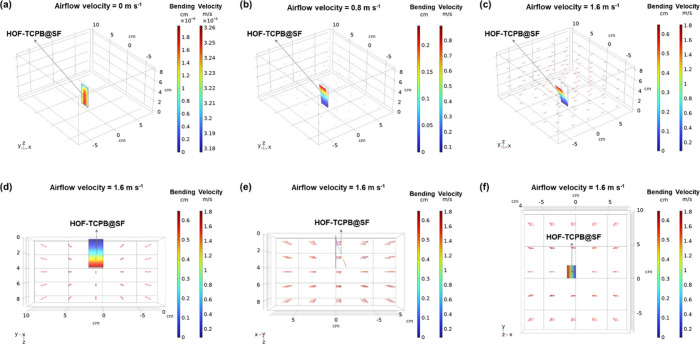
3D FES model of HOF-TCPB@SF airflow sensor in the airflow field under airflow velocities of (a) 0 m s^–1^, (b) 0.8 m s^–1^, and (c) 1.6 m s^–1^, respectively. 2D FES model of the HOF-TCPB@SF airflow sensor in the airflow field at the an airflow velocity of 1.6 m s^–1^ from three different angles including (d) the *x*-axis, (e) the *y*-axis, and (f) the *z*-axis, respectively.

The in-depth sensing mechanism of HOF-TCPB@SF as an airflow sensor was explored. In the airflow field, when the external airflow is transmitted to this airflow sensor, it can bend under motivation via the drag force of the airflows, causing a fluctuation in the optical signal. The sensing process was analyzed based on the Multiphysics field coupling–”fluid-structure interaction” through FES of COMSOL 6.2. And a 3D HOF-TCPB@SF model with a size of 4.0 × 2.0 × 0.02 cm^3^ in the airflow field was constructed. The two color legends in Figure 3 represent the degree of bending of the HOF-TCPB@SF model (cm) and the magnitude of the airflow velocity (m s^–1^), respectively. And the arrows in the airflow field represent airflow. As shown in Figure 3a, when the airflow velocity is 0 m s^–1^, the film is in a straight state without bending. As the airflow speed gradually increases from 0 to 0.8 m s^–1^, the curling degree of the film model increases under the thrust of the airflow (Figure 3b). When the airflow velocity rises to 1.6 m s^–1^, the model reaches the maximum curling extent (Figure 3c), and simultaneously, the fluorescence response variation at this velocity also reaches its maximum value, which is consistent with the trend of airflow sensing performance in the range of 0–1.6 m s^–1^ (Figure 2g). Figure 3d–f displays the model diagrams from three different angles including *x*-axis, *y*-axis, and *z*-axis at airflow velocity = 1.6 m s^–1^. It can be concluded that the bending condition of the film can be clearly observed from three different perspectives and the bending degree of the film gradually increases from the fixed end to the free end according to the color legend. Moreover, Movie S2 reveals the complete dynamic bending process of the 3D HOF-TCPB@SF model with the airflow increasing from 0 to 1.6 m s^–1^, which further demonstrates that the airflow sensing mechanism can be attributed to the variation of the airflow current velocity. And the airflow velocity can change the curvature of the HOF-TCPB@SF, and then induce a fluorescence intensity change.

### Acoustic Sensing and Mechanism

**Figure 4:**
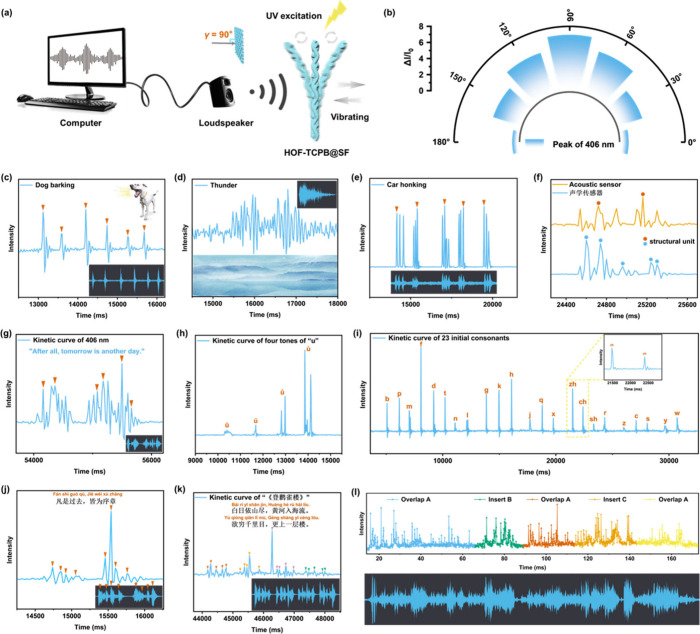
(a) Schematic diagram of the setup for the sound sensing test, where γ represents the sound incidence angle. (b) Dependence of Δ*I*/*I*_0_ at 406 nm on different sound incidence angles γ (0 to 180°). Optical response signal of HOF-TCPB@SF acoustic sensor to (c) dog barking, (d) thunder, and (e) car honking. (f) Recognition of English words and Chinese characters. (g) Recognition signals of English movie lines. (h) Optical signal responses toward the sounds of four tones of “u”. (i) Optical response signals toward the sounds of 23 initial consonants. (j) Optical response signals toward the sound of the Chinese eight-character epigram “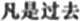,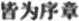,”. (k) Kinetic curve of the sound of landscape poetry “
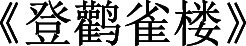
”. (l) Music visualization of a whole rondo of “Fur Elise”.

Sound plays an essential role in perceiving the world, which enables learning, communicating, predicting potential dangers, diagnosing diseases, and much more. In air, the vibration of the sound source produces sound and propagates in the form of acoustic waves, which carries critical information about the sound source. When the acoustic waves collide with the acoustic sensor during propagation, vibration energy can be transmitted, causing intense vibration of the sensor. Benefiting from its low quality, high elasticity, high flexibility, and bright blue fluorescence emission, HOF-TCPB@SF can be an excellent candidate as an acoustic sensor. Herein, to investigate the recognition ability of the sensor for various sounds including biological sounds, geophysical sounds, anthropogenic sounds, textual sounds, and music, we first investigated some basic performances of the sensor. During the evaluation process of sound sensing performance, a series of indexes such as incidence angle, repeatability, response time, sensitivity, DL, RSD, frequency (*f*) response, etc. were studied. The observation of sound signals was achieved by measuring the fluorescence intensity variation at 406 nm through dynamic scanning after the sound source produces sound. The devices for sound sensing are shown in Figure S25. As the schematic diagram of the sound sensing process shows (Figure 4a), when an amplifier connected to a computer produces a sound signal, it will cause the HOF-TCPB@SF-based acoustic sensor to vibrate, leading to a fluctuation in the optical signal (Movie S3). Figure 4b shows that the acoustic sensor not only can respond to sound from the perpendicular direction (incidence angle γ = 90°) but also can detect sound with an oblique direction, manifesting its wide applicable range.

As the sound incidence angle γ changes from 0 to 90° or from 180 to 90°, the fluorescence intensity changes of the sensor gradually increase, which suggests 90° is the optimal direction (Figure S26). As displayed in Figure S27a–e, with the distance increasing from 1 to 5 cm, the fluorescence intensity change gradually decreases. There is a great linear relationship between the relative fluorescence intensity change (Δ*I*/*I*_0_) and distance (*d*), which can be fitted as the equation (Figure S27f)
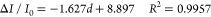
7

And in order to study the stability and repetitiveness of the HOF-TCPB@SF acoustic sensor, the repeatability experiment was conducted. Figure S28 shows that the fluorescent response signal was basically stable without obvious upward or downward fluctuation at 406 nm after 330 cycle responses for a bright drum sound (*f* = 388 Hz, SPL = 110 dB), demonstrating the outstanding recyclability of this acoustic sensor. Further experiments with response time testing were conducted. As shown in Figure S29, when a Chinese ancient poem called 
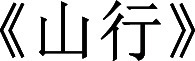
 was played (SPL = 110 dB, γ = 90°), this acoustic sensor can clearly record the sound signal corresponding to each of its four sentences. The response time of the strongest peak sound signal is only 40 ms, proving the ultrafast response speed of this sensor. Loudness, pitch, and timbre are the three primary characteristics of sound, which are the basis of distinguishing sounds. The level of sound loudness can be described by SPL using dB as a unit. To explore the effect of SPL on fluorescence response signals, a sound signal gradually decreased from 110 to 35 dB, and the characteristic curve of SPL change process was recorded. As displayed in Figure S30, with the SPL of the sound decreasing, the vibration of HOF-TCPB@SF sensor gradually weakened, resulting in a gradual decrease in fluorescence intensity changes. And in the range of 110–35 dB, there is a good linear relationship between Δ*I* and SPL, which can be perfectly fitted to the linear equation (Figure S31)
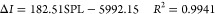
8

Besides, on the basis of the equation^56^
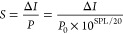
9in which Δ*I* is the difference of emission intensity (*I*) with the sound and initial emission intensity (*I*_0_), *P* is the sound pressure, and *P*_0_ is the reference sound pressure (2 × 10^–5^ Pa), the maximum sensitivity (*S*_max_) was calculated to be 841126.16 cps Pa^–1^ with SPL = 35 dB (Figure S32), which is higher than that in previous work of 330687.96 cps Pa^–1^.^60^ The sensing effective area for HOF-TCPB@SF is 4.0 × 2.0 cm^2^, and the sensitivity per area (figure of merit, FOM_sen_) is 105140.77 cps Pa^–1^ cm^–2^. These results indicate its wide detection range and high sensitivity. The DL of the sensor toward SPL was determined as 0.2980 dB according to eq 4. The performance comparison of the HOF-TCPB@SF acoustic sensor with other acoustic sensors is displayed in Table S3, which indicates the following unique superiorities of this sensor. (i) The sensor shows ultrahigh sensitivity. (ii) This acoustic sensor is based on optical signals, while the previously reported acoustic sensors have nearly been designed on the basis of electrical signal variations. (iii) This sensor shows an ultrafast response time (40 ms), which was not studied in other research. (iv) The DL of this sensor to SPL is as low as 0.2980 dB, whereas other acoustic sensors may not have measured this.^26,56,61−70^Figure S33a,b displays the effect of HOF-TCPB@SF before and after bending on the sound response signal. As shown in Figure S33c, after the bending of HOF-TCPB@SF, the whole response values (Δ*I*/*I*_0_) measured for 10 cycles represented a slight decrease, with the averages changing from 11.34 before bending to 10.92 after bending (Figure S33d), proving that bending has a slight impact on sound detection. Figure S34 presents the boxplot of fluorescence response signals for sounds (*f* = 388 Hz, γ = 90°) of 110, 90, 70, 60, 50, 40, and 35 dB under 10 parallel measurements (Figure S35a–g), which reveals the mean and the standard deviation of the response signals. Taking SPL = 110 dB as an example, the mean and standard deviation were 14843 ± 320 cps. Correspondingly, within the SPL range of 110–35 dB, the RSD was calculated to be less than 3.5%, demonstrating the high stability and reliability of HOF-TCPB@SF acoustic sensor for different SPL recognition (Figure S35h). Moreover, Chinese classical poetry “
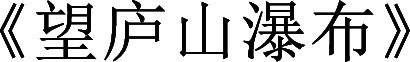
” with 10 cycles was recorded (Figure S36), the optical signal wave forms of which are in good accord with those of the electrical signal, suggesting the high fidelity and high practicality of the HOF-TCPB@SF acoustic sensor. Pitch is a significant parameter of sound that is determined by *f*. Figure S37 shows the different fluorescence responses of the HOF-TCPB@SF acoustic sensor under diverse frequencies. When the sensor receives the specific frequency signal, it will generate a “bridge-shaped” response signal, and the height difference of “bridge-shaped” signals will change with frequencies. As displayed in Figure S38, this sensor can detect sound within 200–500 Hz, and the height difference increases gradually in the range of 200–375 Hz, while it decreases in the range of 375–500 Hz. Hence, the optimal response frequency of the HOF-TCPB@SF acoustic sensor to sound is 375 Hz. Timbre is a kind of signal that heavily represents one’s own personality, which is the standard for distinguishing different people’s voices. When two identical words are spoken by male and female, respectively, the different peaks are exhibited, with males showing stronger fluctuations in their vocal signals than females (Figure S39).

Therefore, this acoustic sensor can also be used for the discrimination of timbre. Figure S40a shows that the influence of the temperature on sound sensing is relatively weak. As revealed in Figure S40b, Δ*I*/*I*_0_ of the HOF-TCPB@SF acoustic sensor toward sound slightly increases (6.50–7.39) as the temperature increases from room temperature (22 °C) to 100 °C, proving the great anti-interference ability of the sensor toward temperature during sound sensing.

Additionally, a series of sounds including biological sounds, geophysical sounds, anthropogenic sounds, textual sounds, and music were identified by this sensor. Soundscapes, as the collection of sounds perceived in the environment, consist of biological sounds (e.g., bees), geophysical sounds (e.g., wind), and anthropogenic sounds (e.g., traffic noise), in which natural sounds are beneficial for psychological well-being while noise can lead to health problems.^71^ Therefore, detecting and distinguishing these sounds can attain environmental information, thereby further improving human health level and living experience. With regard to various biological sounds including dog, wolf, bees, tiger, and rooster, their fluorescence response signals are displayed in Figure 4c and Figure S41. It can be noticed that all of the signals exhibit a high signal-to-noise ratio, with recognizable and unique waveforms for every sound, endowing the HOF-TCPB@SF sensor with the novel ability to distinguish various sounds. There is also a high degree of consistency between the optical and the original electrical signals of these sounds. The geophysical sounds containing thunder, waves, and wind can also be clearly identified (Figure 4d and Figure S42). Natural sounds can help alleviate the increasingly serious mental health and anxiety problems of modern people; therefore, the HOF-TCPB@SF acoustic sensor can lay the foundation for exploring the impact of sound on health. Meanwhile, this acoustic sensor can recognize traffic noises such as car honking, train whistle, and subway electric whistle, which is meaningful for noise prevention and control (Figure 4e and Figure S43). Moreover, the ability of the HOF-TCPB@SF acoustic sensor to recognize texts and languages was explored. The sound “acoustic sensor” was repeated using English and Chinese, respectively. As shown in Figure 4f, the smallest structural unit of languages (English word and Chinese character) can be detected explicitly, indicating the desirable discriminative ability of the sensor. The excellent recognition ability is more prominent in long sentences. Some English movie lines are shown in Figure 4g and Figure S44, and it can be observed that the sensor can detect almost every word in these sentences. In modern Chinese phonetics, the minimum unit of speech is a syllable (Chinese character), which is composed of three basic elements: tone, initial consonant, and final vowel. Tone refers to the inherent high and low sounds within Chinese syllables that can distinguish meaning, which include “high and level tone”, “rising tone”, “falling-rising tone”, and “falling tone”. The four tones of six vowel letters can be clearly distinguished by this sensor (Figure 4h and Figure S45). Besides, the HOF-TCPB@SF acoustic sensor also displays an excellent differentiation effect on the 23 brisk initials and 24 loud finals (Figure 4i and Figure S46). When receiving the sound of Chinese eight-character epigrams, this sensor can output its own corresponding spiky response signals, and these signals match well with electrical signals; hence, the different contents of these epigrams can be studied in depth (Figure 4j and Figure S47). Landscape poetries are used by poets to express their devotion for natural scenery. After the written ancient poetry is converted into voiced language, the acoustic sensor can convert the detected sound signals into an image display. Consistent with the original electrical signal, the optical response images can not only display a particular response signal of each word in sentences but also accurately identify pauses in specific sentences, manifesting the capacity to collect extremely complex and weak signals (Figure 4k and Figure S48). Music compositions, as another form of sound carrier, can only show continuity and fluidity at the time level, without an actual-existence spatial property. Nevertheless, music visualization can present music information through a visual way of graphic images to enhance the expressiveness of music.^72,73^Figure S49 shows the optical signal variation of the HOF-TCPB@SF acoustic sensor stimulated by the sound of seven different musical notes (“Do”, “Re”, “Mi”, “Fa”, “So”, “La”, and “Ti”), the fundamental elements in music, at the same SPL (110 dB). It can be observed that different musical notes result in different output profiles, proving its potential in music imaging. To verify its ability to visualize music, the complete response signals of the piano piece “Fur Elise” were recorded by HOF-TCPB@SF sensor. As shown in Figure 4l, different colors represent various rondo sections, as “ABACA”. The visual images can fully display almost all the attributes of music signals, enabling people to intuitively feel the beauty of melody from a visual perspective. Due to its ultrafast response speed, the optical signals of the sensor display high synchronization with the original electrical signals. And there is a significant difference between different sections, indicating the complete music presentation ability and persistent stable working characteristics of the acoustic sensor. Hence, it is convinced that the HOF-TCPB@SF acoustic sensor possesses promising development prospects in the sound visualization field.

**Figure 5:**
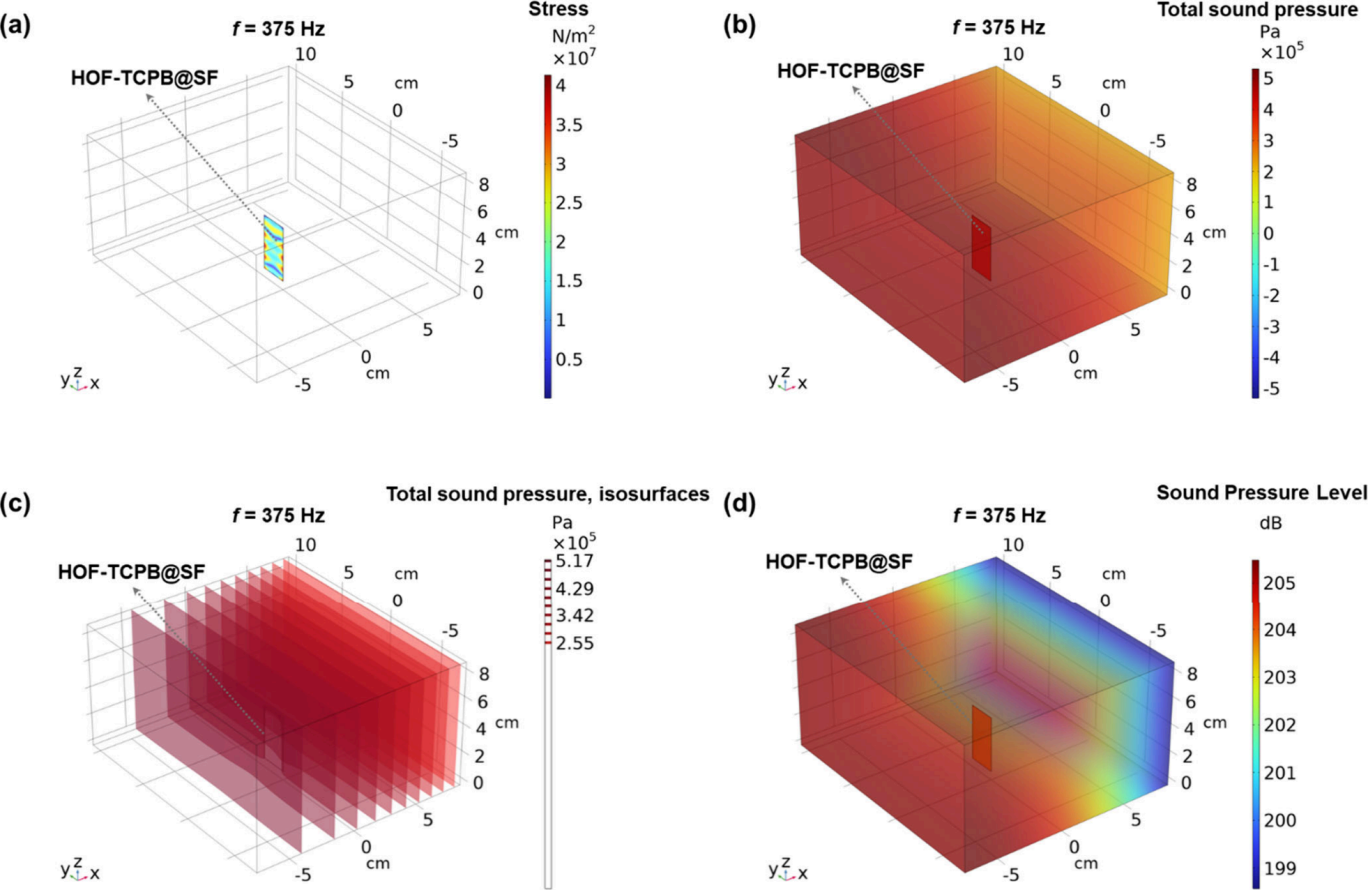
(a) Stress distribution of the 3D FES model of HOF-TCPB@SF acoustic sensor in the sound field when *f* = 375 Hz. (b) Total sound pressure distribution of the 3D FES model of HOF-TCPB@SF acoustic sensor in the sound field when *f* = 375 Hz. (c) Total sound pressure isosurface distribution of the 3D FES model of HOF-TCPB@SF acoustic sensor in the sound field when *f* = 375 Hz. (d) Sound pressure level distribution of the 3D FES model of HOF-TCPB@SF acoustic sensor in the sound field when *f* = 375 Hz.

The working mechanism of the HOF-TCPB@SF acoustic sensor was further investigated. As displayed in Movie S3, in the sound field, when the external sound emitted by a loudspeaker is transmitted to the HOF-TCPB@SF acoustic sensor, it can vibrate under the action of air vibrations, resulting in a fluctuation in the optical signal. To elucidate the working mechanism of the acoustic sensor, we built up a 3D model of HOF-TCPB@SF in the sound field (Figure 5). The sensing process was analyzed based on the Multiphysics field coupling–“sound-structure interaction” via FES of COMSOL 6.2. This model simulates two parts for actual sound propagation, including the sound field (18 × 15 × 10 cm^3^) and HOF-TCPB@SF acoustic sensor (4.0 × 2.0 × 0.02 cm^3^). In actual measurement, this sensor exhibits a clear response to the sound within 200–500 Hz (Figure S38), with the strongest fluorescence response at 375 Hz. Hence, we simulated the response of the sensor to sound in the range 200–500 Hz. The output results contain stress, total sound pressure, total sound pressure isosurfaces, and the sound pressure level distribution of the sound field. As shown in Figure S50, the dependence of total sound pressure on sound frequency obtained by FES indicates that in the range of 200–375 Hz, the total sound pressure gradually increases with increasing frequency. At 375 Hz, the total sound pressure reaches its maximum value and then gradually decreases with increasing frequency. The simulated result is in accordance with the curves that were actually measured (Figure S38), which demonstrates the reliability of the simulated results. Figure 5a–d represents the stress (N/m^2^), total sound pressure (Pa), total sound pressure isosurfaces (Pa), and sound pressure level (dB) distribution of the sound field at 375 Hz. According to the color legend, it can be inferred that these physical quantities are all high at 375 Hz, further proving the strong fluorescence response at 375 Hz. Moreover, in line with Figure S30 and the equation
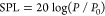
10in which *P* and *P*_0_ are the sound pressures and reference sound pressure (2 × 10^–5^ Pa), it can be obtained that the sound pressure is proportional to the fluorescence response Δ*I*/*I*_0_. Movie S4 reveals the change process of stress and total sound pressure, which further proves the 3D FES model total sound pressure distribution and the stress distribution on HOF-TCPB@SF are the highest when *f* is at 375 Hz, proving that HOF-TCPB@SF exhibits the highest fluorescence response Δ*I*/*I*_0_ at 375 Hz. In summary, the sensing process of sound can be attributed to the changes in sound causing changes in sound pressure and stress, in turn leading to changes in air and HOF-TCPB@SF vibration and ultimately resulting in the variations in fluorescence response.

### Intelligent Applications of Airflow–Acoustic Bimodal Sensor Based on Human-Computer Interaction

**Figure 6:**
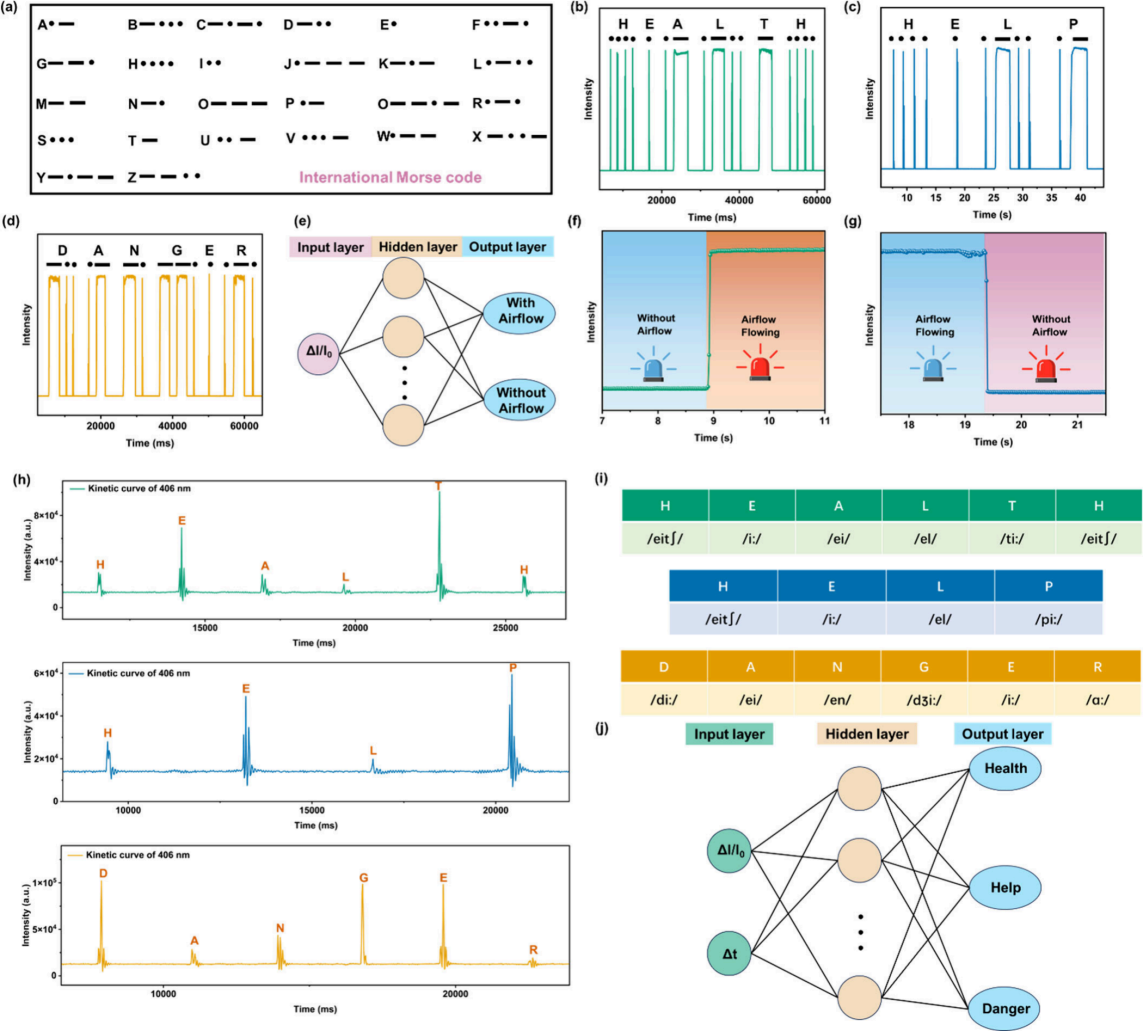
(a) International Morse password comparison table. (b) Transmitting the Morse code for the word “HEALTH”. (c) Transmitting Morse code for the word “HELP”. (d) Transmitting Morse code for the word “DANGER”. (e) Structure of BPNN1 for real-time monitoring of the presence or absence of airflow under different conditions. (f) Condition I for the intelligent airflow detection platform. The blue LED corresponds to the absence of airflow (output: “0”) and the red LED corresponds to the existence of airflow (output: “1”). (g) Condition II for the intelligent airflow detection platform. The blue LED corresponds to the existence of airflow (output: “1”) and the red LED corresponds to the absence of airflow (output: “0”). (h) Optical signal wave forms of sounds “HEALTH”, ““HELP”, and “DANGER” obtained by HOF-TCPB@SF acoustic sensor. (i) Pronunciations of “H, E, A, L, T, H”, “H, E, L, P”, and “D, A, N, G, E, R”. (j) Structure of BPNN3 for the recognition of “Health”, “Help”, and “Danger”.

The potential applications of the HOF-TCPB@SF airflow–acoustic bimodal sensor, including Morse code-assisted respiratory information expression, real-time airflow monitoring, intelligent speech recognition, and airflow–acoustic interoperability, were further explored. Benefiting from the advantages of rapid response and recovery speed to airflow, the HOF-TCPB@SF as an airflow sensor could be applied for information encryption and communication. In accordance with Morse code (Figure 6a), each English letter can be standardized using the sequences of two different signals: dots “·” and lines “–”. The instantaneous airflow represents a dot “·” in Morse code and the lasting airflow for a few seconds signals the line “–” in the Morse code. For instance, the words “health”, “help”, and “danger” can be outputted by blowing the airflow with continuous short and long duration based on the Morse code method (Figure 6b–d). Owing to the high sensitivity, high efficiency, and great stability, the HOF-TCPB@SF airflow sensor can also be used to monitor respiratory status and possesses the potential to diagnose diseases relevant with breath. Figure S51a shows that this airflow sensor can detect respiratory states such as normal, apnea, shortness, and hypopnea when a tester breathes toward the sensor. As displayed in the blue zone in Figure S51a, when the tester breathes normally and slightly, the optical signal variation shows a relatively low frequency and low intensity. In the case where the tester stops breathing, the fluorescence intensity remains basically unchanged, which indicates “apnea”. The optical signal frequency increases when the tester is under “shortness”, while the optical response signal lasts for a long time under “hypopnea” conditions. Integrating the Morse code transmission with the sensitive response to breath, the HOF-TCPB@SF airflow sensor could assist people who cannot speak or write to convey information and express needs. For example, patients with quadriplegic aphasia can convey “HELP” information to others by controlling their breathing patterns (Figure S51b), which is significant for the development of medical healthcare services and medical devices.

Furthermore, integrated intelligent airflow detection platforms were constructed by combining the HOF-TCPB@SF airflow sensor with a human–computer interaction system—a back-propagation neural network (BPNN). The working mechanism of BPNN is as follows. First, during the procedure of forward propagation, the input signal was transferred from neurons of the hidden layer to the output layer through the activation function (FANN_SIGMOID_SYMMETRIC) calculation. Provided the desired results cannot be output from the output layer, the MSE change values of the output layer were calculated, and subsequently the MSE signals were transferred back along the original connection path to modify the weight of neurons at each layer until the desired goal was achieved. Two BPNNs have been built up: BPNN1 is utilized for real-time monitoring of the presence or absence of airflow under different conditions, and BPNN2 is employed to analyze the airflow velocity. For BPNN1, the relative fluorescence intensity variation (Δ*I*/*I*_0_) as input information was inputted into BPNN1 with or without airflow (output: “1” or “0″, respectively) as output information was outputted from BPNN1 (Figure 6e and Table S4), which can efficiently confirm the presence of trace or instantaneous airflow. The network structure information for BPNN1 is shown in Table S5, which indicates that the input layer, hidden layer, and output layer of BPNN1 contain 1 neuron, 6 neurons, and 2 neurons, respectively. And the training function of BPNN is “FANN_TRAIN_RPROP”. The intelligent airflow detection platform based on BPNN1 can work in two conditions. Figure 6f displays condition I of the platform, in which the green LED signifies the absence of airflow (“0”) and the red LED indicates that a constant airflow blows in (“1”). Condition I can be applied to prevent the infiltration of polluted airflow in a superclean room. Correspondingly, as shown in Figure 6g, condition II suggests that the red LED will illuminate when the airflow suddenly disappears (“0”), which is suitable for real-time monitoring of airflow in underground tunnel engineering to ensure the circulation of fresh air, preventing workers from suffocation and avoiding the accumulation of toxic gases. The mean squared errors (MSEs) for the BPNN1 system were lower than 1.37 × 10^–10^ (Table S6), demonstrating that BPNN1 possesses an ultrahigh accuracy (>99.99%) for determining the presence of airflow. And the corresponding Matlab code of BPNN1 is given in Table S7.

To realize the real-time sensing function to airflow velocity (*v*), another intelligent airflow detection platform based on BPNN2 was constructed; all the information of BPNN2 has been placed in Tables S8–S11. According to the Δ*I*/*I*_0_–*v* relationship (Figure 2g), the relative intensity variations (Δ*I*/*I*_0_) as input information were inputted into BPNN2, and the airflow velocities could be outputted (Figure S52). And the calculated MSEs of BPNN2 were below 4.36 × 10^–7^ (*v* = 0.2 m s^–1^) based on the original output matrix (Table S10). Therefore, BPNN2 can be utilized for wind speed testing in environmental monitoring or accurate weather forecasting. Moreover, both BPNN1 and BPNN2 can achieve the real-time human–computer interaction for airflow state detection and airflow velocity detection, which facilitates their applications in actual measurements (Figures S53 and S54).

HOF-TCPB@SF as an acoustic sensor can record speech information and convert it into a spectrogram signal. Based on human–computer interaction techniques, the fluctuation peak information corresponding to each letter or word can be analyzed, enabling one to output textual information in real time and with accuracy. This process can achieve speech recognition and transformation from speech to text. Herein, an intelligent speech recognition platform was constructed by combining BPNN3 with an HOF-TCPB@SF acoustic sensor to apply to real-time word information detection. In the acoustic sensing process, the pronouncing optical signal curves of 26 English letters were first recorded as a pronunciation database (Figure S55). Then a sequence of sounds such as “H, E, A, L, T, H”, “H, E, L, P”, and “D, A, N, G, E, R” were recorded as spectrograms by kinetic curves (Figure 6h). And the pronunciations of these letters are shown in Figure 6i. The relative fluorescence intensity variations (Δ*I*/*I*_0_) for the strongest fluctuation for each letter pronunciation as input information were inputted in BPNN3 (Figure 6j and Table S12). For instance, while Δ*I*/*I*_0_ (“1.29”, “4.09”, “1.22”, “0.55’, “6.15”, and “1.09”) for “H, E, A, L, T, H” was inputted in BPNN3, respectively, the relevant word “health” could be outputted (Figure 6j), which indicates that a real-time human–computer interaction from voice messages to text messages has been implemented (Figure S56). Similarly, “help” and “danger” can also be outputted by the training of BPNN3. Table S13 displays the network structure information of BPNN3. The entire speech information recognition procedure has a low deviation (MSEs < 3.88 × 10^–7^) and high accuracy (accuracy >99.99%), suggesting the high reliability and practicality of the intelligent sound recognition system in the field of speech recognition and word conversion (Table S14). And the Matlab code of BPNN3 is revealed in Table S15. This intelligent recognition platform can help human obtain real-time audio information through vision, improving the efficiency of communication and information acquisition, and also assist hearing-impaired people to obtain accurate sound information. Additionally, the platform is similar to the voice input function of smartphones, which can convert the voices spoken by people into texts and output them, indicating its huge application prospects in the future intelligent speech recognition field.

Since textual information can be obtained through both airflow and sound signals (Figure 6b–d,h), conversely, the corresponding spectrograms of airflow and sound can be obtained simultaneously when using text information as an output signal outputted from BPNN4. All of the information for BPNN4 is shown in Tables S16–S19. The recorded optical signal responses of English pronunciation of the order numbers for 26 English letters—numbers 1–26—are exhibited in Figure S57. For example, the order numbers in 26 English letters and Δ*I*/*I*_0_ values of these order numbers (“8, 3.1”, “5, 0.7”, “12, 1.24”, and “16, 2.65”) are input as information for BPNN4 (Figure S57), and the output text result “help” could be obtained (Figure S58 and Table S16). And the airflow Morse code spectrograms and sound spectra corresponding to “help” can be further inferred (Figure 6c,h), which builds a bridge between airflow and sound signals through textual information. The real-time human–computer interaction of BPNN4 can also be achieved (Figure S59), which achieves the assembly and interoperability of airflow and sound signals. Spiders can simultaneously monitor airflow and sound signals through spider webs, thereby achieving perception and capture of prey. By simulating the function of spider webs, dual-monitoring of airflow and sound signals can be achieved, which can predict the surrounding environmental conditions more accurately, enhancing the robustness of recognition. Moreover, dual-mode information perception possesses a significantly broad application prospect in fields such as interactive artificial intelligence and multimodal robot perception. Moreover, the simultaneous detection of sound and airflow signals plays a unique role in fields such as information monitoring, early warning reconnaissance, meteorological detection, and stealth target detection.

## Conclusions

In summary, motivated by the network structure and airflow–acoustic perception capability of a spider web, we prepared “spider web-like” HOF-TCPB@SF as an optical airflow–acoustic bimodal sensor using an efficient one-step dip-coating approach. HOF-TCPB@SF possesses the merits of high flexibility, high elasticity, light weight, and excellent fluorescence emission. As an airflow sensor, HOF-TCPB@SF shows an ultralow DL (0.0076 m s^–1^), ultrahigh sensitivity (12.48 m^–1^ s), ultrafast response and recovery time (40 ms), and excellent repeatability (480 cycles). During the process of acoustic sensing, the HOF-TCPB@SF acoustic sensor has ultrahigh sensitivity (FOM_sen_ = 105140.77 cps Pa^–1^ cm^–2^), ultrahigh precision (RSD < 3.5%), ultrafast response time (40 ms), ultralow DL (0.2980 dB), and great recyclability (330 cycles), which can also identify various sounds such as natural sounds, anthropogenic sounds, English/Chinese voices, and music. The highest response frequency toward sound is 375 Hz. Both the airflow and acoustic sensing procedures exhibit multiangle recognition response (0–180°), and their perception mechanisms are analyzed in detail through FES of COMSOL 6.2. By combining Morse code and human respiratory status, this airflow sensor can be designed into personal assistive devices. Besides, based on human–computer interaction technology, this bimodal sensor can not only realize real-time airflow monitoring and speech recognition with high accuracy but also achieve airflow–acoustic interoperability. The superior performances of the airflow–acoustic bimodal sensor indicate its promise in wide potential applications in human health detection, weather forecasting, tunnel engineering, warning reconnaissance, intelligent textiles, and smart bionics.

## Supporting Information Available

Materials and methods, PXRD patterns, SEM images, EDX energy spectra, luminescence spectra, kinetic curve spectra, and BPNN data (PDF)
Movies as described in the text (ZIP)
