# Supplementary material for: Bioinspired Photoluminescent “Spider Web” as Ultrafast and Ultrasensitive Airflow–Acoustic Bimodal Sensor for Human–Computer Interaction and Intelligent Recognition
Source: ACS Cent Sci. 2024 Sep 26;10(10):1894–909. doi: 10.1021/acscentsci.4c01182 (PMC11503498; doi:10.1021/acscentsci.4c01182)
Supplement: Supplementary file 1 — oc4c01182_si_001.pdf [file oc4c01182_si_001.pdf]

Supporting Information

Bioinspired Photoluminescent "Spider Web" as Ultrafast and Ultrasensitive Airflow-Acoustic Bimodal Sensor for Human-Computer Interaction and Intelligent Recognition

Kai Zhu[a] and Bing Yan[a] *

School of Chemical Science and Engineering, Tongji University, Siping Road 1239, Shanghai 200092, China [a]

* Corresponding author: Email address: byan@tongji.edu.cn (Bing Yan)

Experimental Section

Materials and Methods

1,2,4,5-tetrakis (4-carboxyphenyl) benzene (H_4TCPB) were purchased from Adamas-beta and used without further purification. Silk film (SF), anemometer, and loudspeaker were purchased from Taobao Mall.

SEM was performed on a Hitachi S-4800 field emission scanning electron microscope operating at 3 kV. Energy dispersive X-ray spectroscopy (EDX) and the EDX mapping image were obtained by the scanning electron microscope operating at 15 kV. Powder X-ray diffraction (PXRD) patterns were collected with a Bruker D8 ADVANCE diffractometer using Cu *Kα* radiation at 40 mA and 40 kV. Fourier transform infrared (FT-IR) spectra were recorded using a Nicolet IS10 infrared spectrophotometer. TGA curve was collected on a Bruker Tensor-II TGA 55 under the N_2 atmosphere with a heating rate of 10 °C from 30 to 800 °C. The fluorescence spectra and kinetic scan curves were recorded on an Edinburgh FLS920 spectrophotometer with a 450 W xenon lamp as an excitation source. Photoluminescence lifetime measurements were measured on the Edinburgh FLS920 spectrophotometer with a microsecond lamp (100 mW).

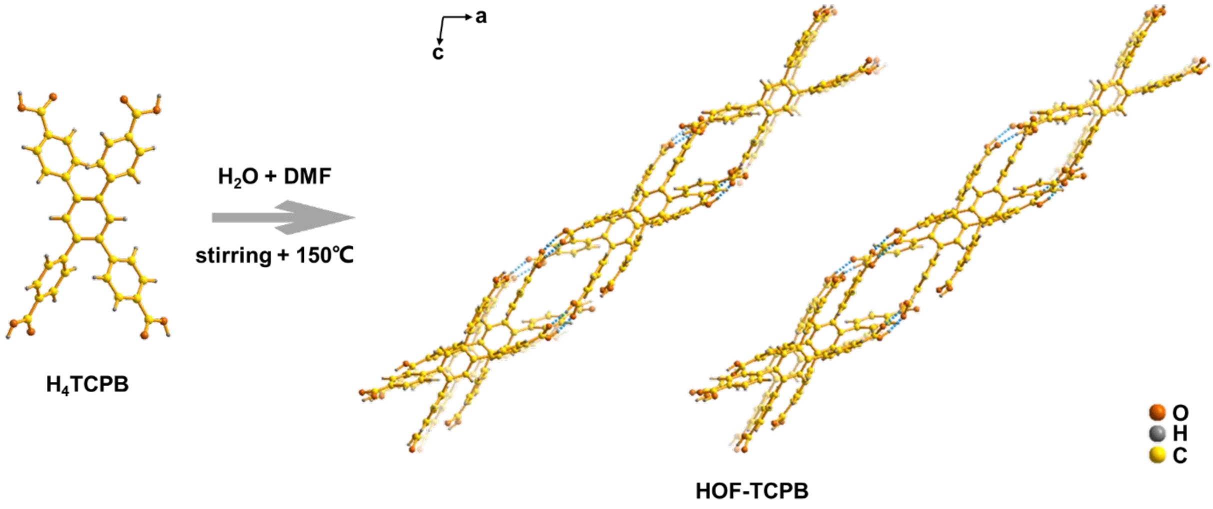

Figure S1. The graphical illustration of the synthesis of HOF-TCPB.

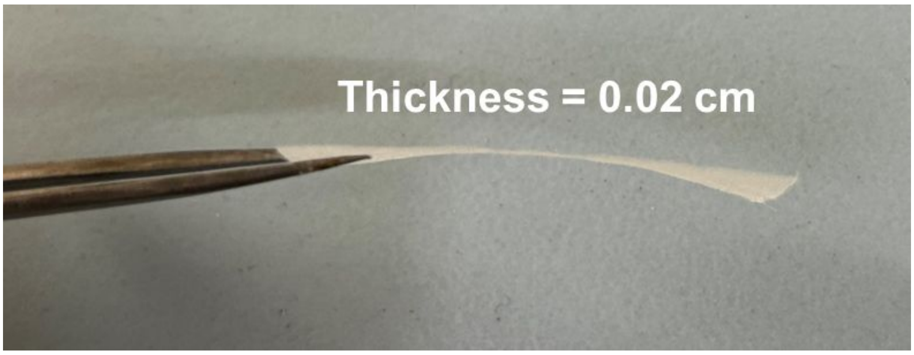

Figure S2. Picture of HOF-TCPB@SF with thickness of 0.02 cm under natural light.

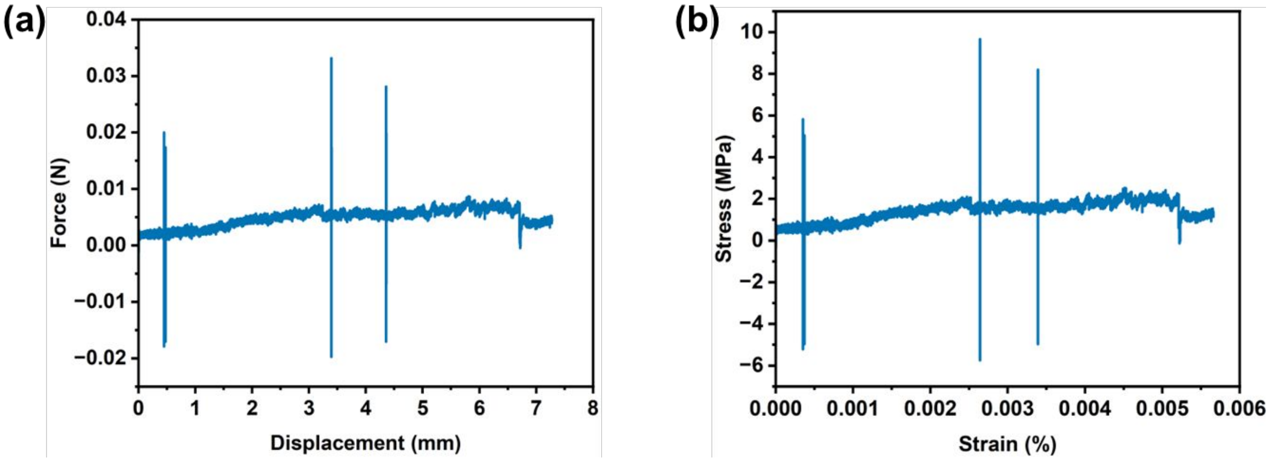

Figure S3. (a) The relationship between force and displacement in bending stiffness testing of SF. (b) The relationship between stress and strain in bending stiffness testing of SF.

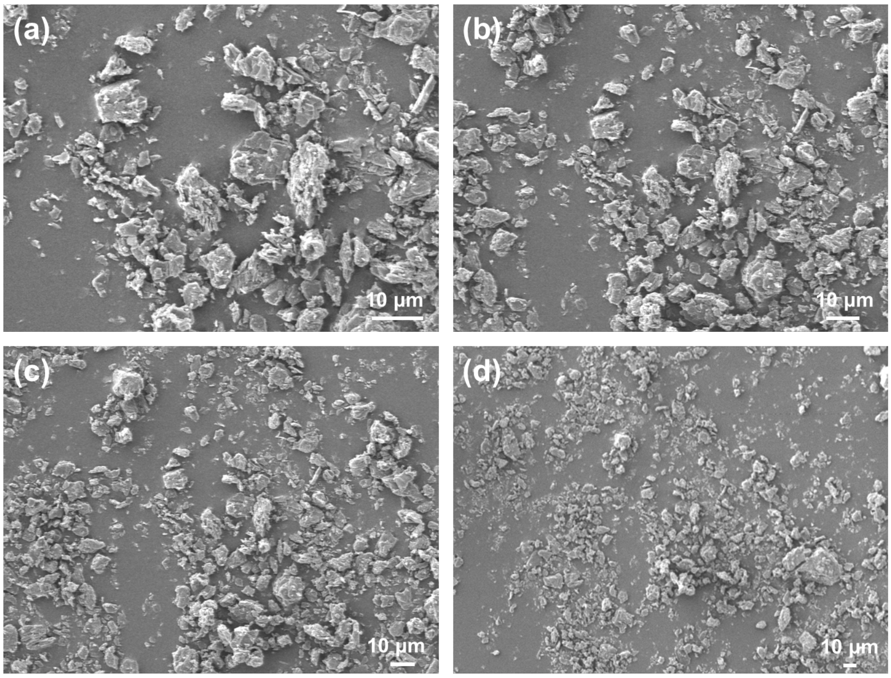

Figure S4. (a) (b) (c) (d) SEM images of HOF-TCPB crystal.

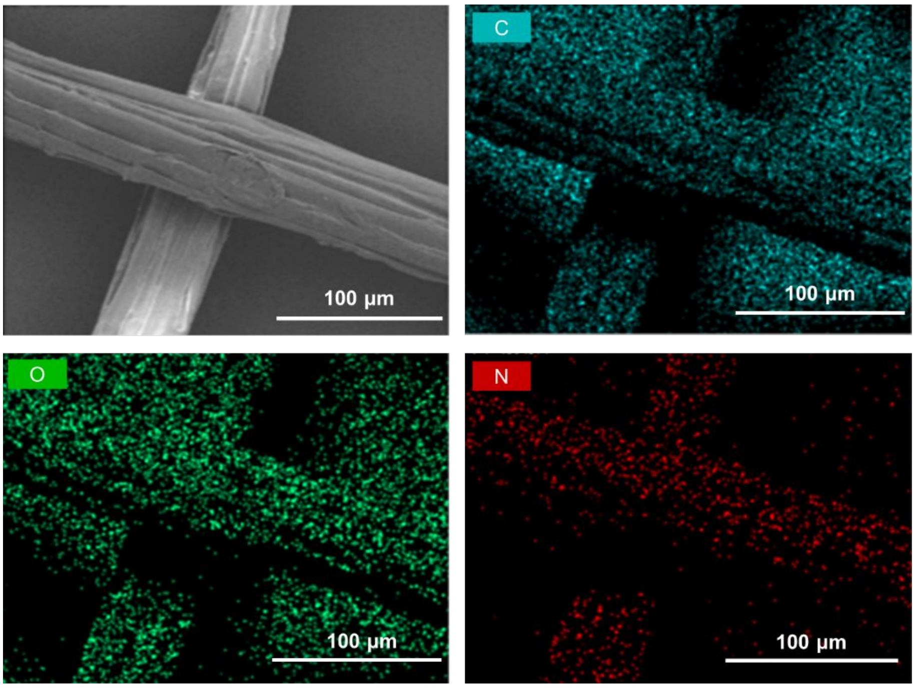

Figure S5. EDX mappings of C, O and N elements in SF.

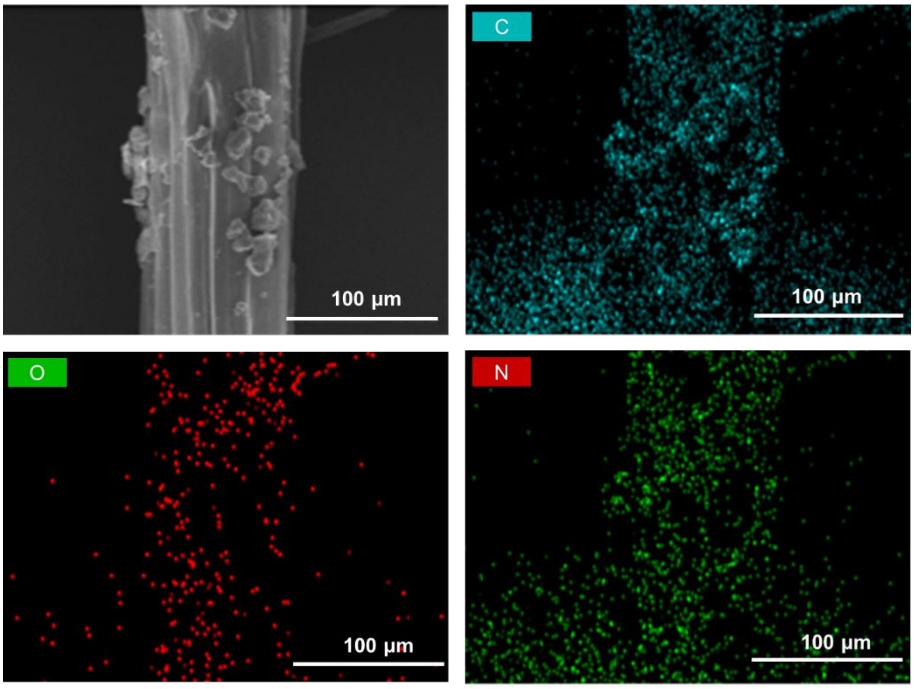

Figure S6. EDX mappings of C, O and N elements in HOF-TCPB@SF.

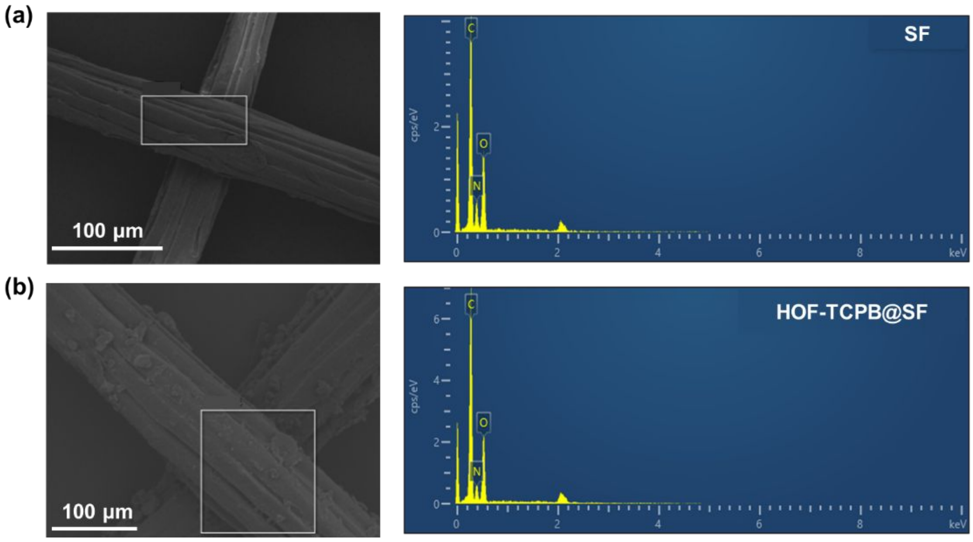

Figure S7. (a) SEM image and corresponding EDX energy spectrum of C (53.79%), O (28.14%) and N (18.08%) elements of SF. (b) SEM image and corresponding EDX energy spectrum of C (57.91%), O (28.97%) and N (13.13%) elements of HOF-TCPB@SF.

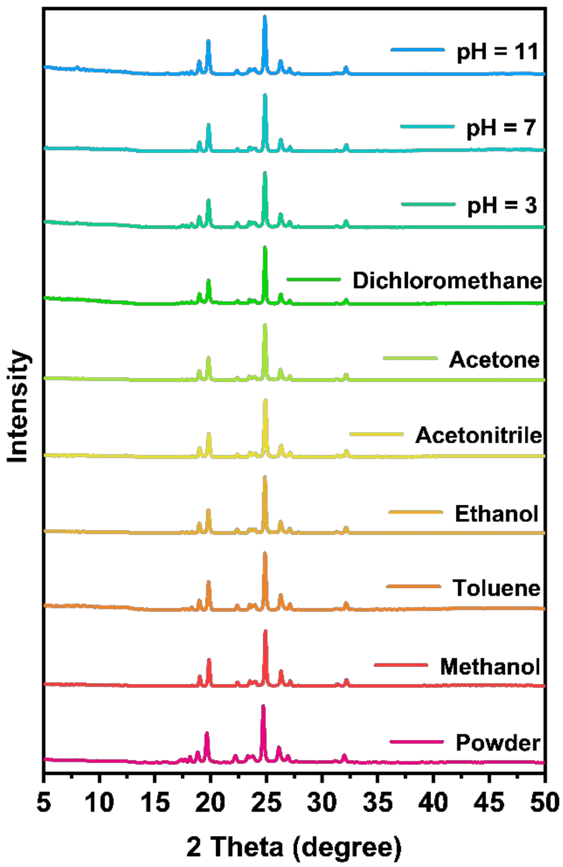

Figure S8. (a) PXRD patterns of HOF-TCPB powder and HOF-TCPB taken out from methanol, toluene, ethanol, acetonitrile, acetone, dichloromethane, acidic (pH = 3), neutral (pH = 7) and alkaline (pH = 11) aqueous solutions for 24 h.

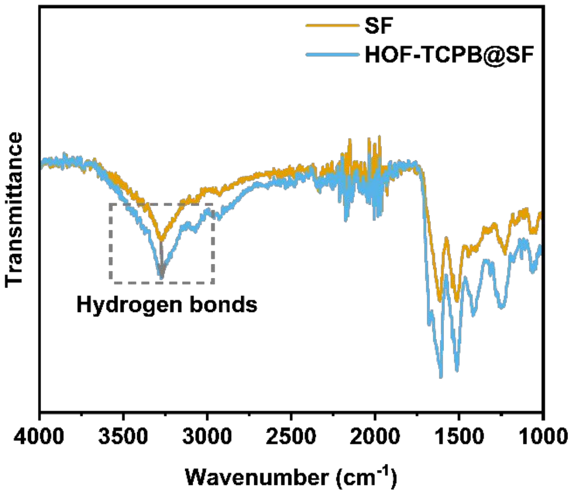

Figure S9. FT-IR spectra of HOF-TCPB, and HOF-TCPB@SF used for the comparison of the 3100 – 3600 cm^{-1} absorption peaks.

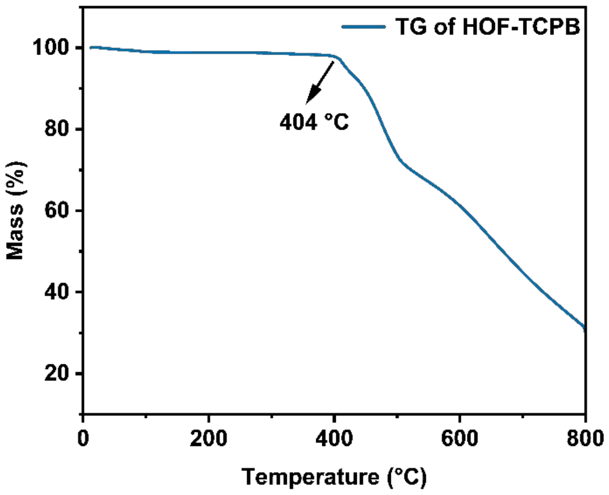

Figure S10. TGA analysis of HOF-TCPB.

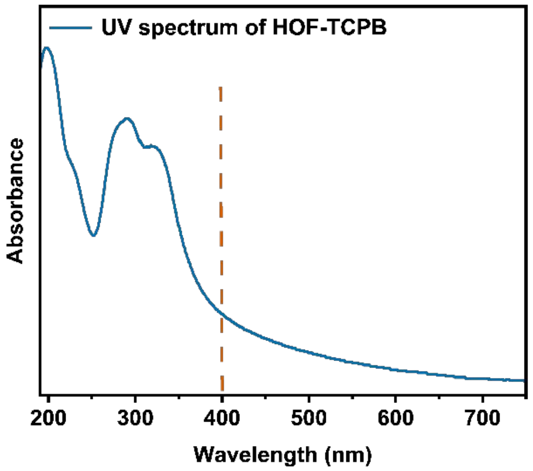

Figure S11. UV absorption spectrum of HOF-TCPB.

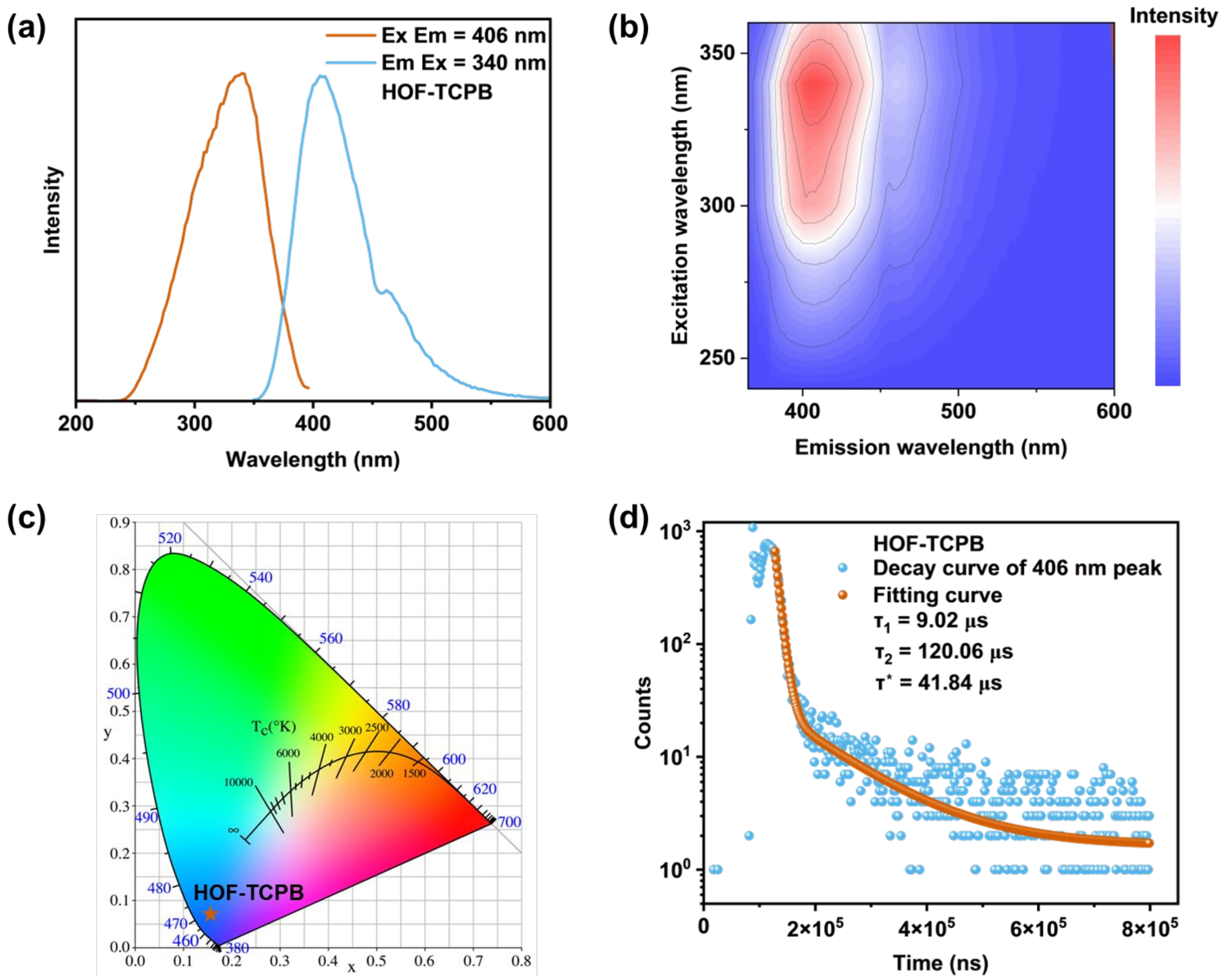

Figure S12. (a) Excitation and emission spectra of HOF-TCPB. (b) Excitation-emission mapping of HOF-TCPB (λ_{ex} = 240 − 360 nm). (c) CIE chromaticity coordinates of HOF-TCPB powder (0.1578, 0.0713). (d) Decay lifetime of 406 nm emission peak for HOF-TCPB.

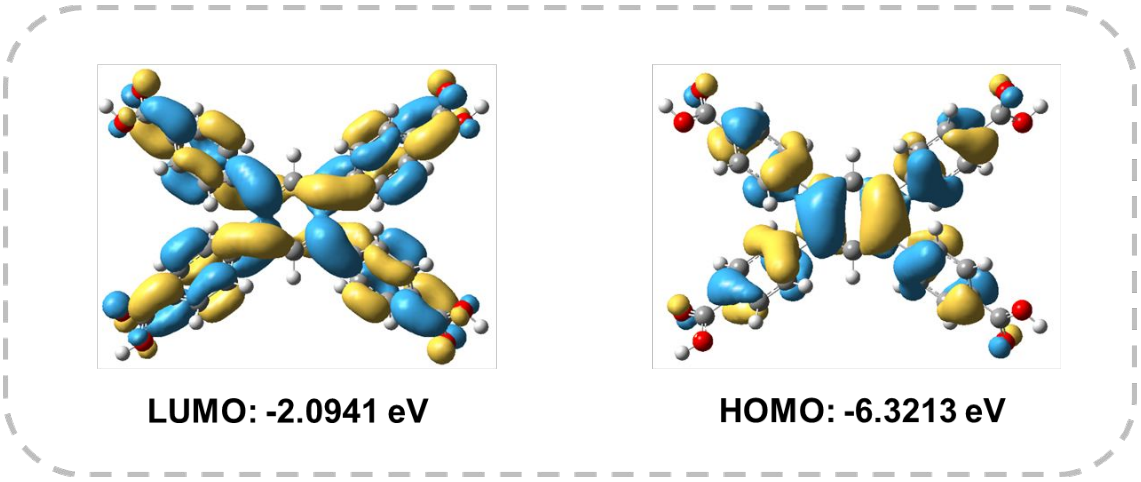

Figure S13. Lowest unoccupied molecular orbital (LUMO) and highest occupied molecular orbital (HOMO) energy level of H_4TCPB.

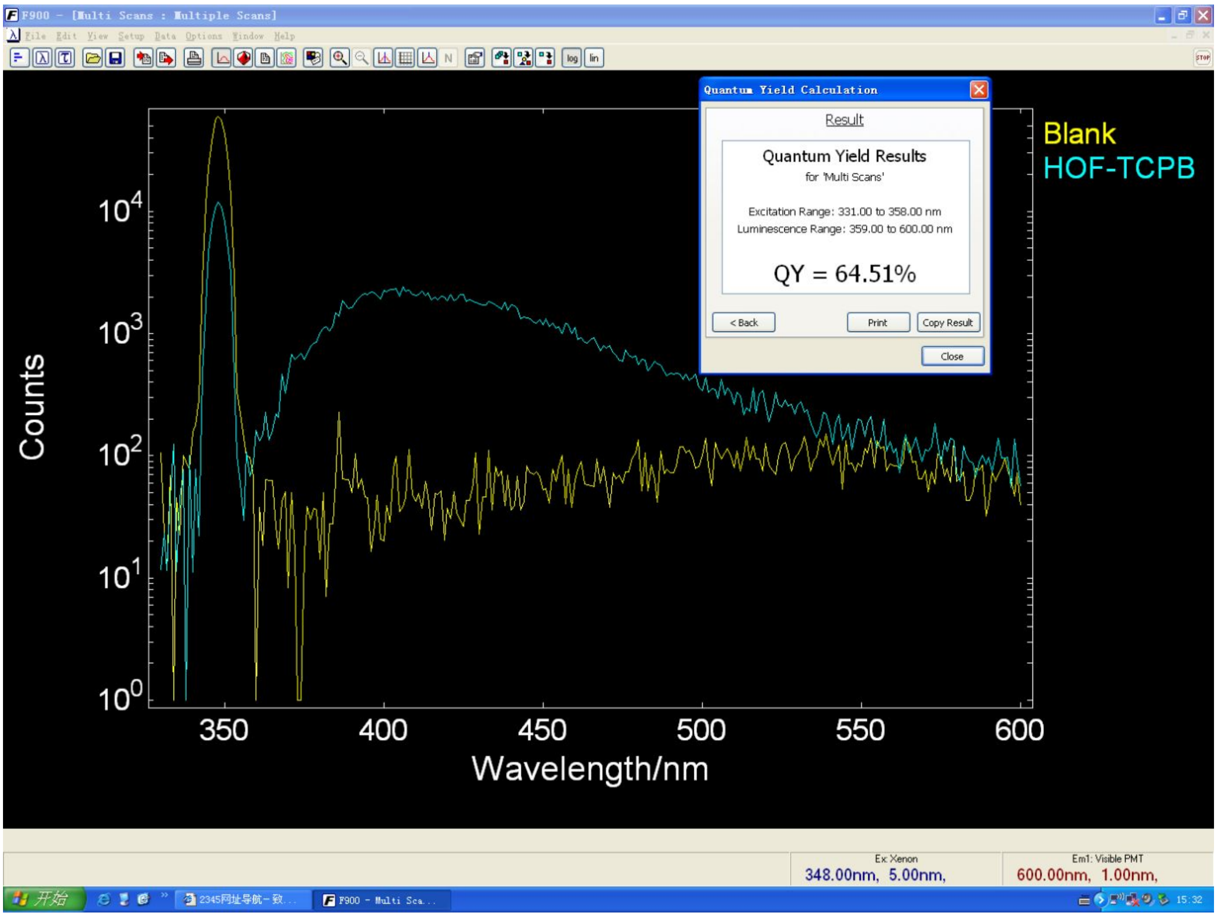

Figure S14. The photoluminescence quantum yield (PLQY) of HOF-TCPB.

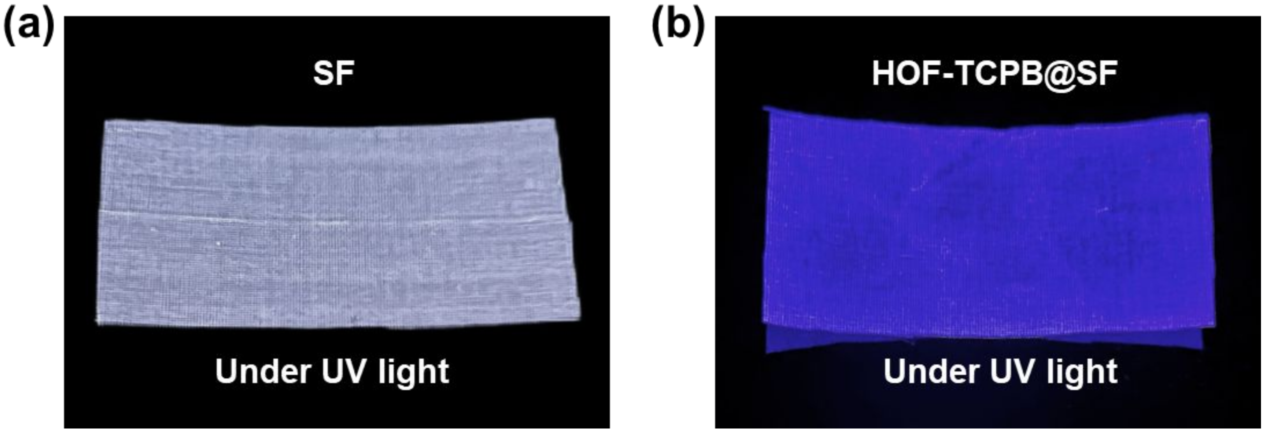

Figure S15. (a) Silk film (SF) under a 310 nm UV light. (b) HOF-TCPB@SF under a 310 nm UV light.

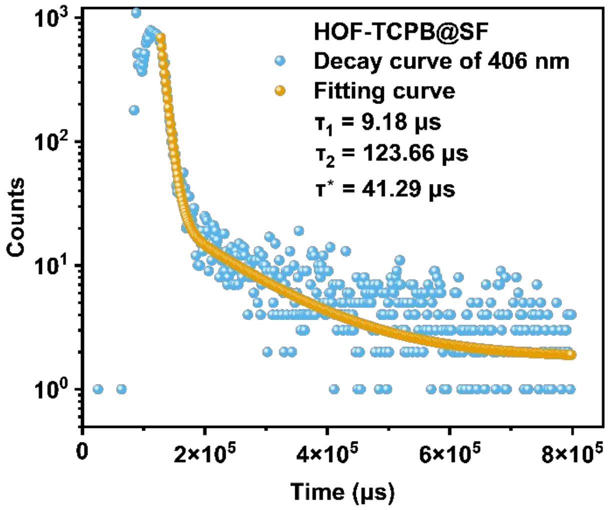

Figure S16. Decay lifetime of 406 nm emission peak for HOF-TCPB@SF (λ_{ex} = 324 nm).

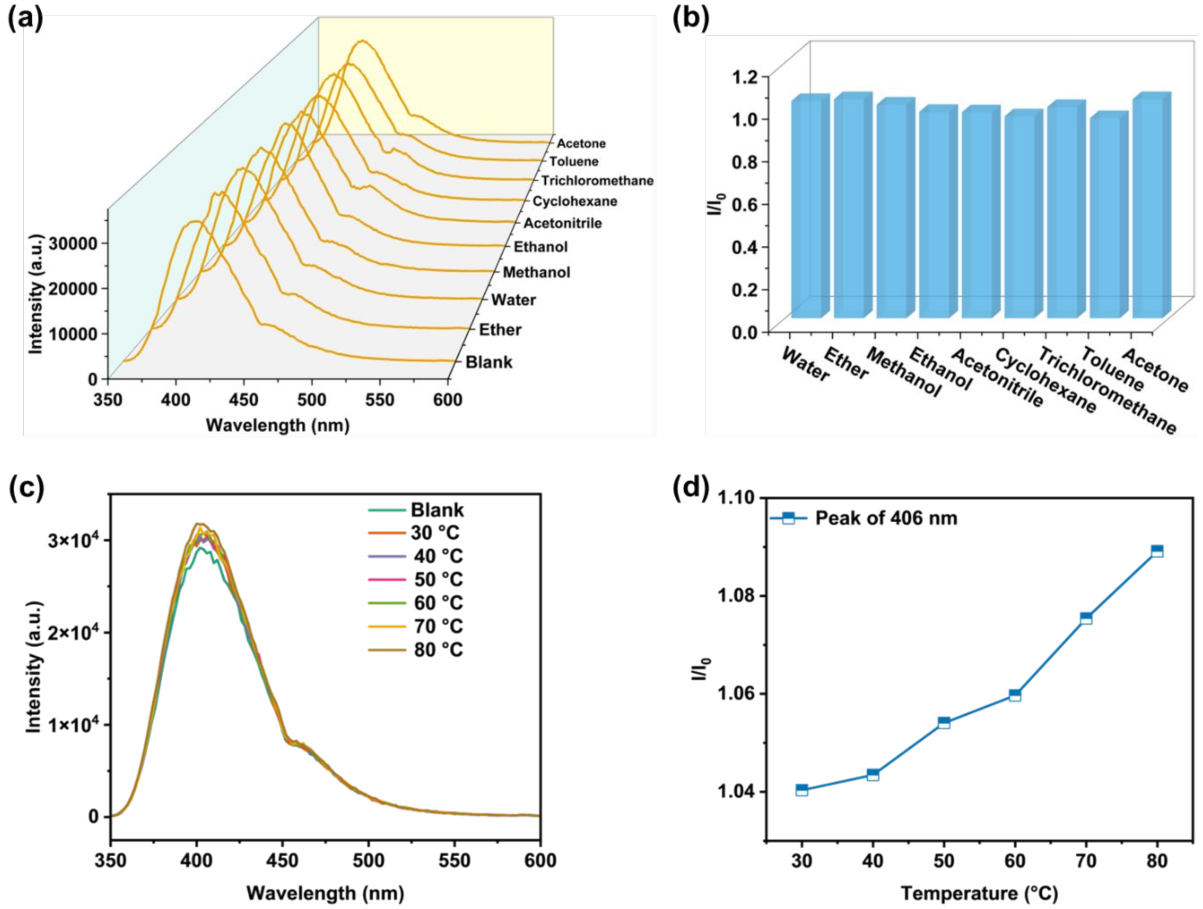

Figure S17. (a) Emission spectra of HOF-TCPB@SF in various atmospheres of volatile gases including water, ether, methanol, acetonitrile, cyclohexane, trichloromethane, toluene and acetone. (b) Histogram for relative fluorescence intensity variation (I/I_0) for HOF-TCPB@SF in various atmospheres of volatile gases. (c) Emission spectra of HOF-TCPB@SF in the temperature range of 30 – 80 °C under λ_{ex} = 324 nm. (d) Line chart of I/I_0 for HOF-TCPB@SF in the temperature range of 30 – 80 °C upon λ_{ex} = 324 nm.

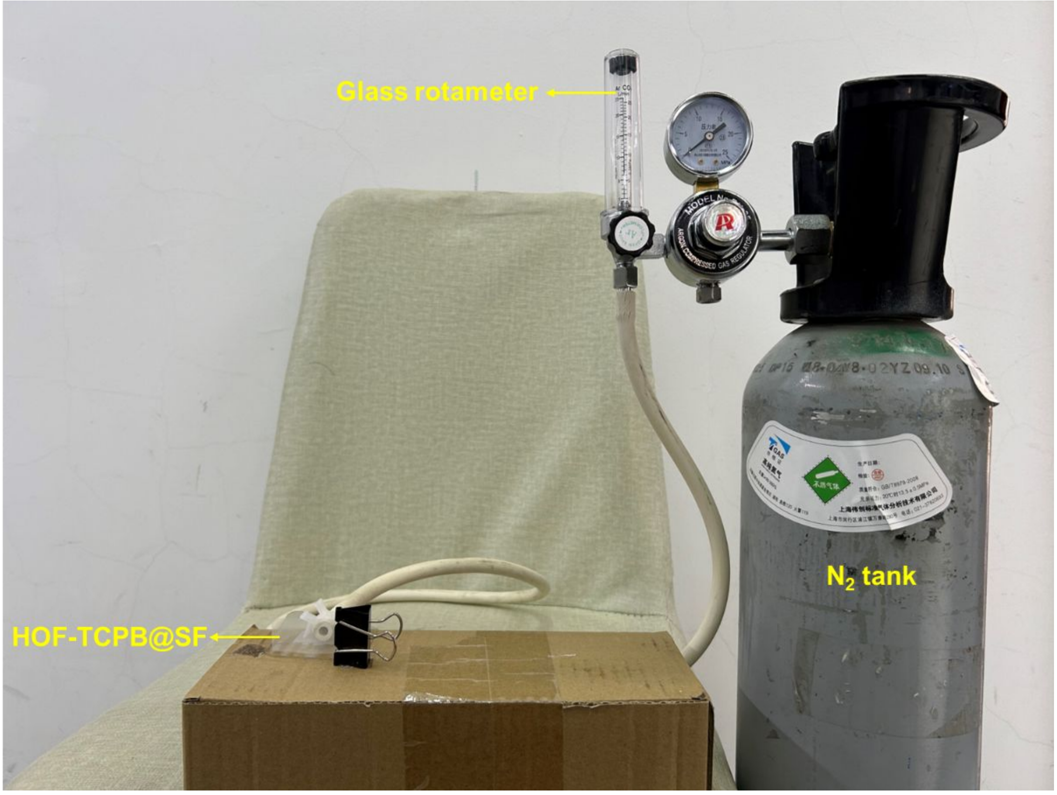

Figure S18. (a) Pictures of the setup for airflow test.

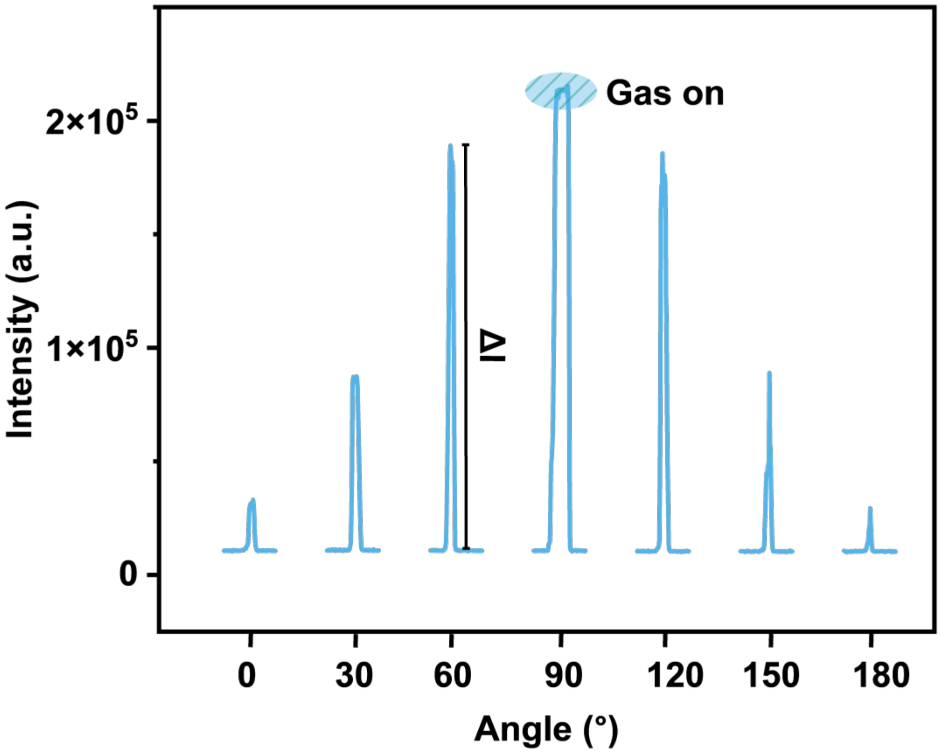

Figure S19. Optical response signal at 406 nm of the HOF-TCPB@SF airflow sensor to the airflow of 1.6 m s^{-1} with airflow blowing angles θ of 0°, 30°, 60°, 90°, 120°, 150°, and 180°, respectively.

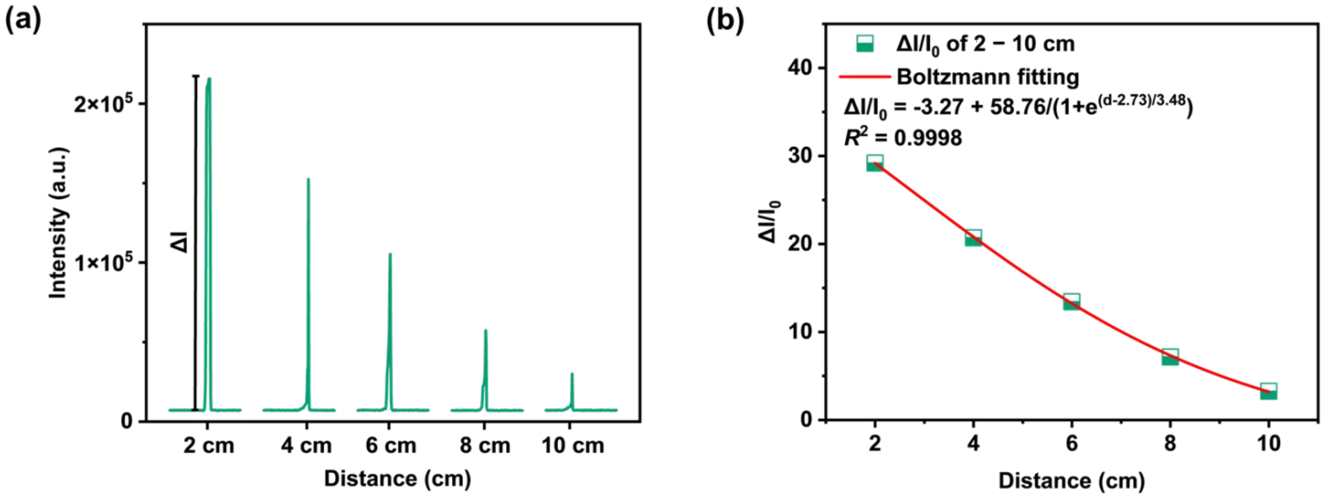

Figure S20. (a) Optical response signal at 406 nm of the HOF-TCPB@SF airflow sensor to the airflow (θ = 90°) of 1.6 m s^{-1} at distances of 2 cm, 4 cm, 6 cm, 8 cm, and 10 cm, respectively. (b) The $\Delta I/I_0$-distance relationship for HOF-TCPB@SF airflow sensor.

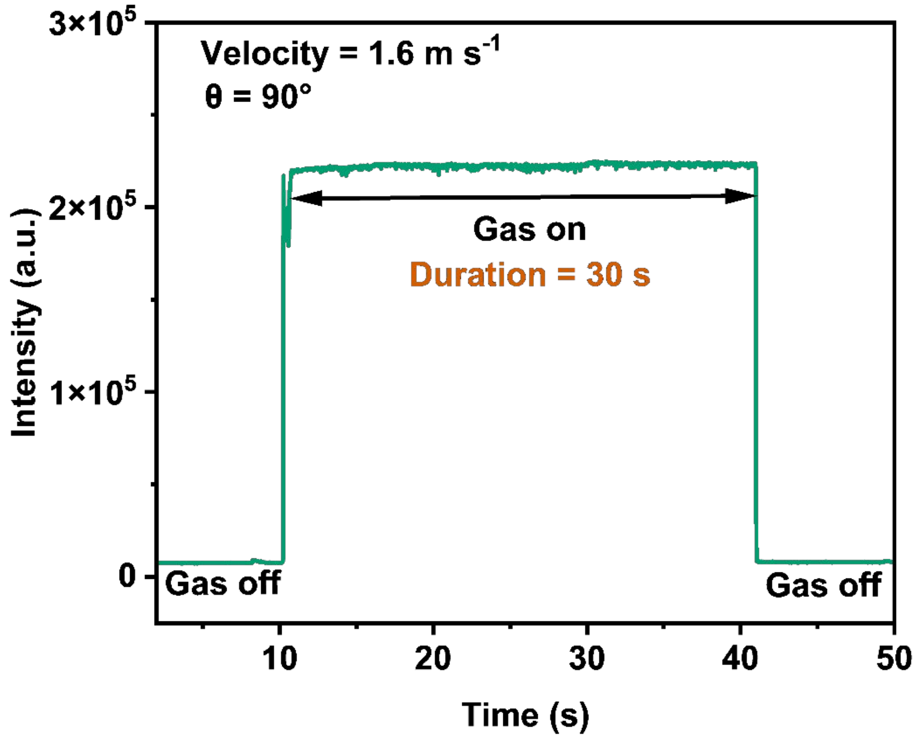

Figure S21. Kinetic curve of HOF-TCPB@SF airflow sensor at 406 nm for airflow (velocity = 1.6 m s^{-1}, airflow blowing angles θ = 90°) stability testing within 30 s duration.

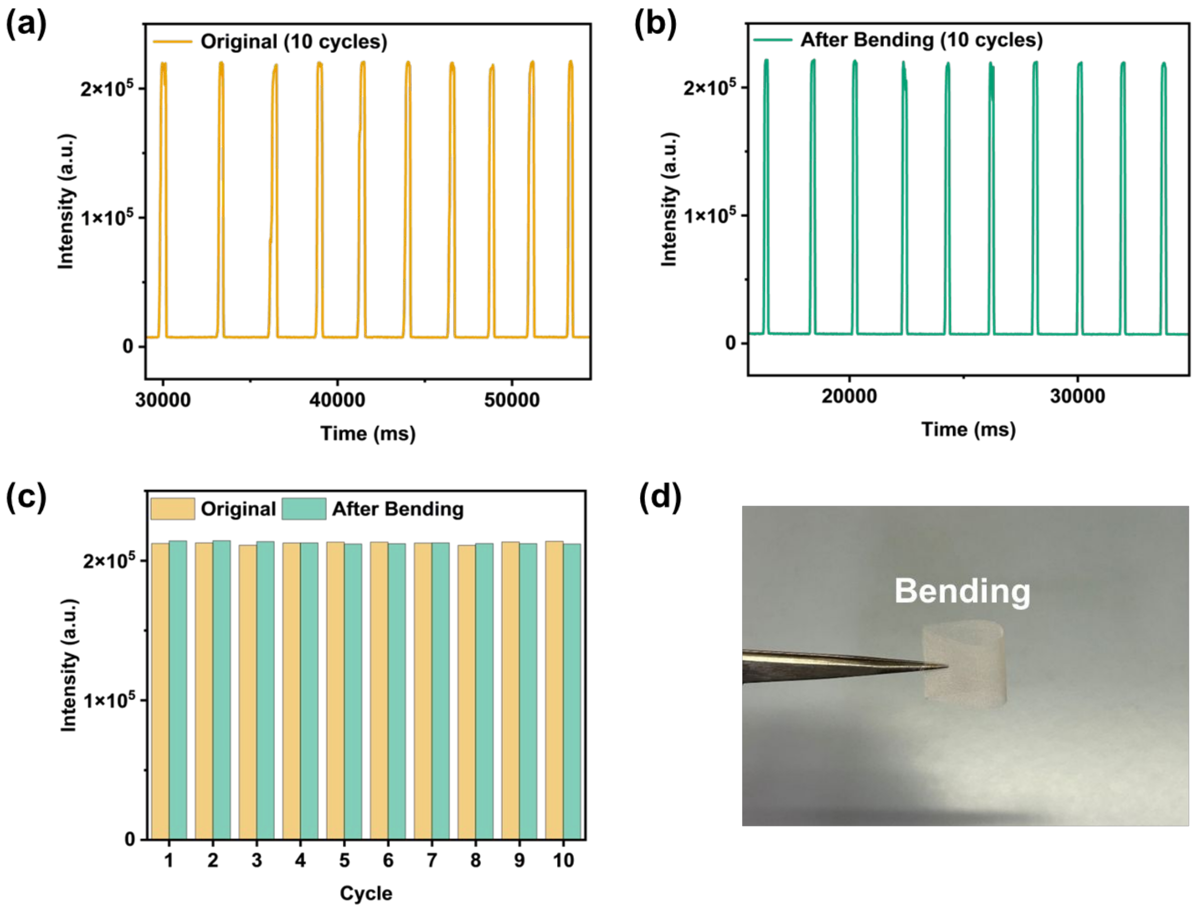

Figure S22. Optical response signal at 406 nm of the HOF-TCPB@SF airflow sensor to the airflow (θ = 90°, velocity = 1.6 m s^{-1}) for 10 cycles (a) before bending, and (b) after bending. (c) Histogram of fluorescence intensity variation of the HOF-TCPB@SF airflow sensor to the airflow (θ = 90°, velocity = 1.6 m s^{-1}) before and after bending. (d) Picture of HOF-TCPB@SF under bending condition.

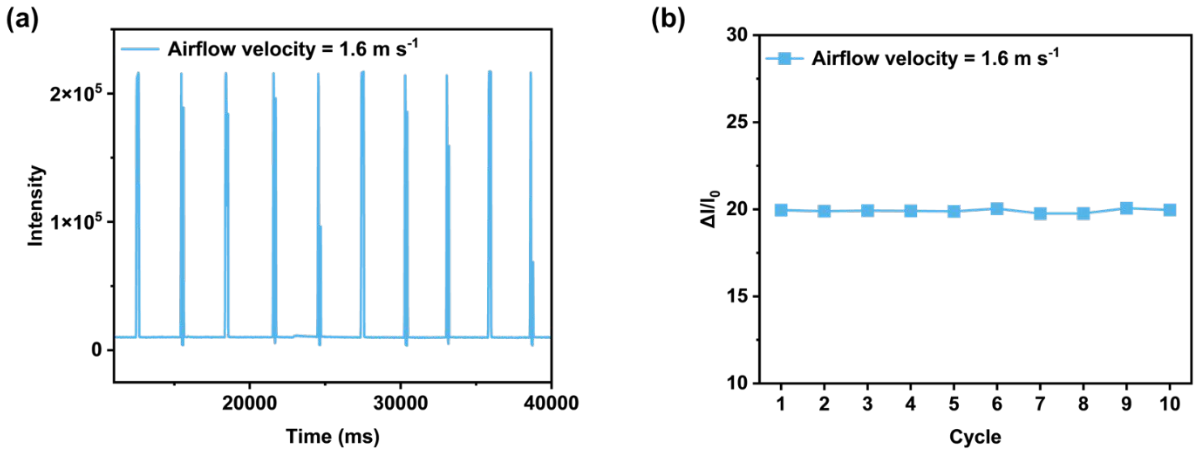

Figure S23. (a) 10 cycles repeated measurement of airflow velocity = 1.6 m s^{-1} for HOF-TCPB airflow sensor utilized for the calculation of RSD. (b) The relative fluorescence intensity variation ($\Delta I/I_0$) of HOF-TCPB@SF for 10 cycles repeated measurement of airflow velocity = 1.6 m s^{-1}.

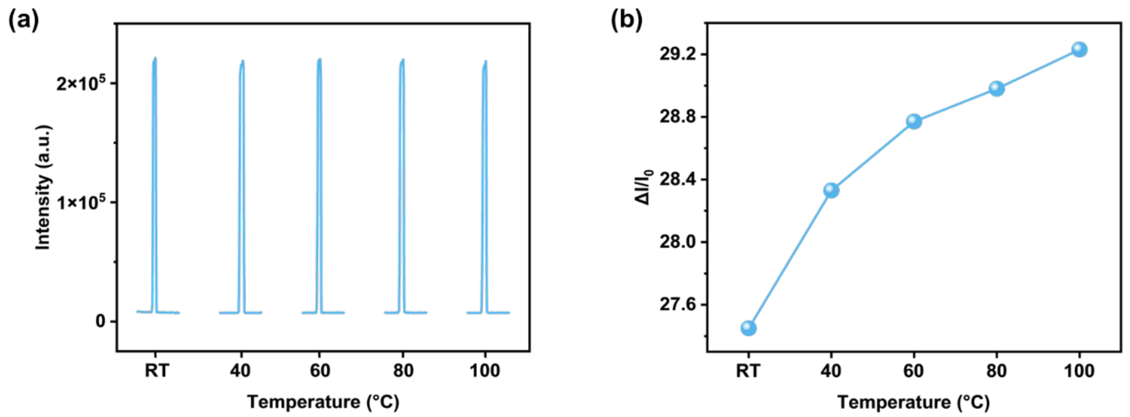

Figure S24. (a) Optical response signal at 406 nm of the HOF-TCPB@SF airflow sensor to the airflow ($\theta = 90°$) of 1.6 m s^{-1} at temperatures of room temperature (RT, 22 °C), 40 °C, 60 °C, 80 °C, and 100 °C, respectively. (b) The relationship between relative fluorescence intensity variation ($\Delta I/I_0$) and temperature.

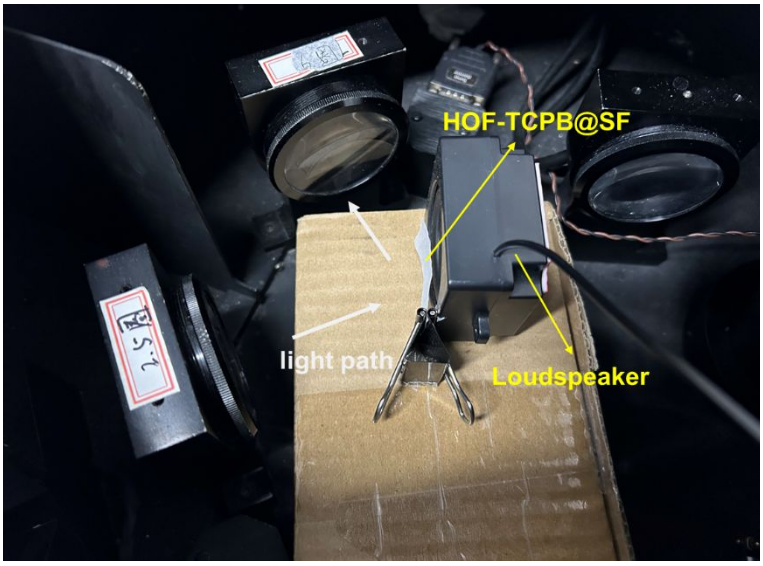

Figure S25. Picture of sound sensing measurement system.

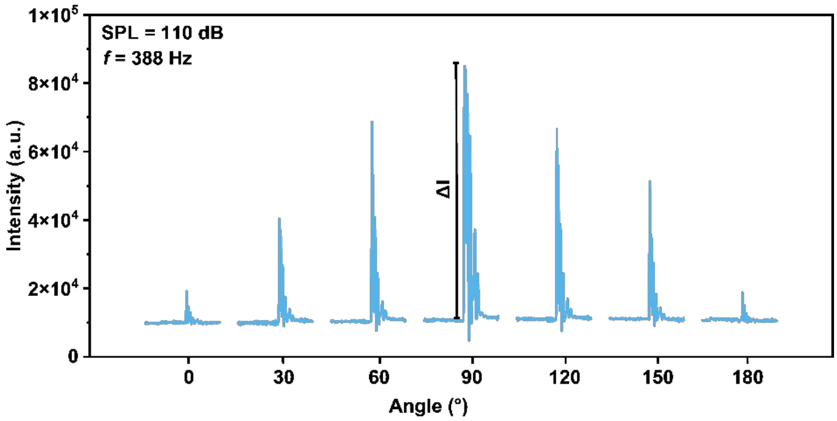

Figure S26. Optical response signal at 406 nm of the HOF-TCPB@SF acoustic sensor to sound (SPL = 110 dB, $f = 388$ Hz) with sound incidence angle γ of 0°, 30°, 60°, 90°, 120°, 150°, and 180°, respectively.

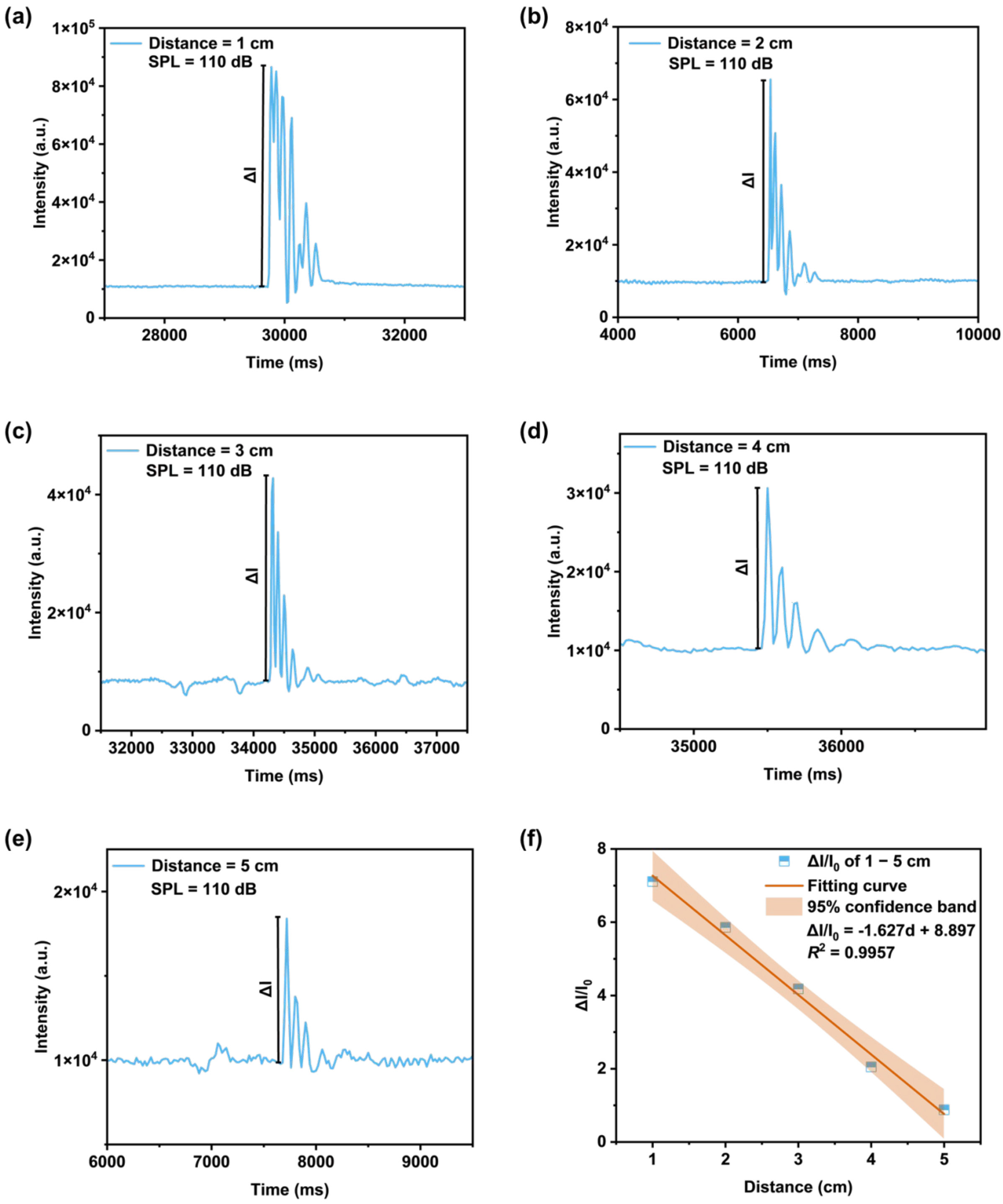

Figure S27. Fluorescence response at 406 nm of the HOF-TCPB@SF acoustic sensor to the sound of 110 dB at a distance of (a) 1 cm, (b) 2 cm, (c) 3 cm, (d) 4 cm, and (e) 5 cm. (f) Linear fitting curve at 406 nm of the HOF-TCPB@SF acoustic sensor to the sound of 110 dB at different distances.

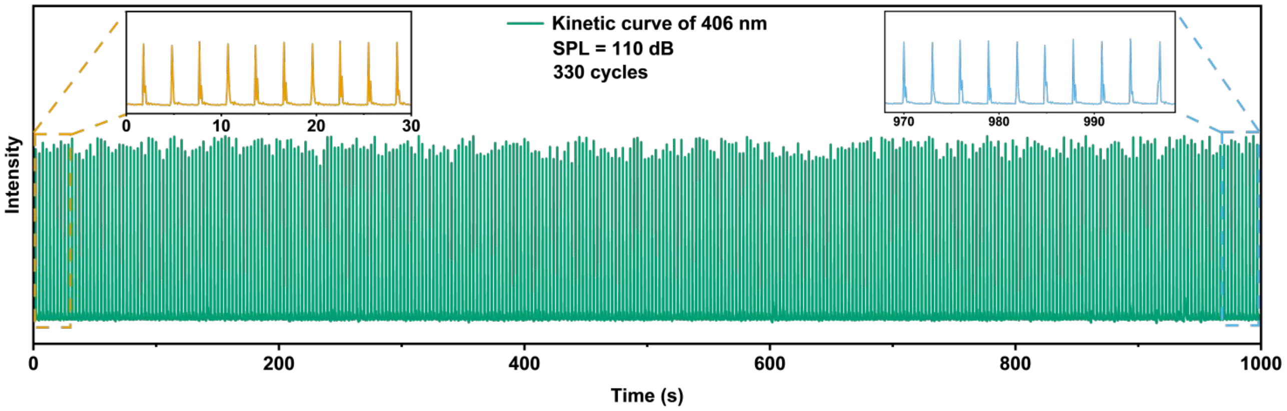

Figure S28. The optical response of the HOF-TCPB@SF acoustic sensor under 330 cycles for monitoring the sound with SPL = 110 dB. The enlarged two figures represent the initial and final ten cycles.

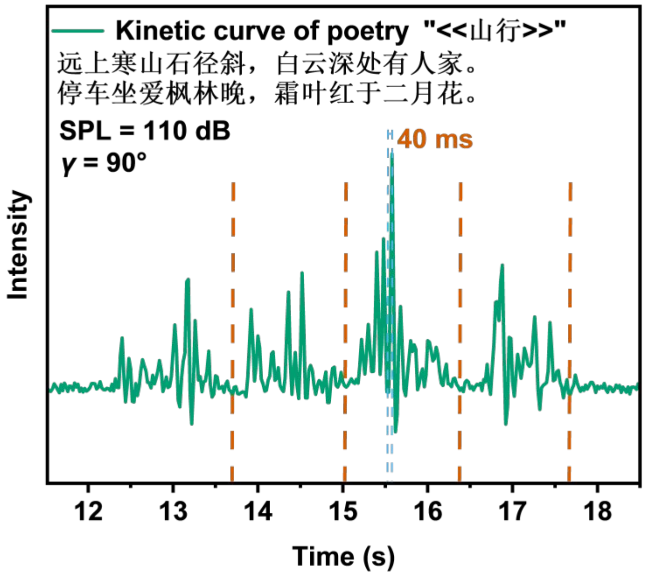

Figure S29. The response time of the HOF-TCPB@SF acoustic sensor to sound (SPL = 110 dB, γ = 90°) through analyzing the speech of a Chinese ancient poem called《山行》.

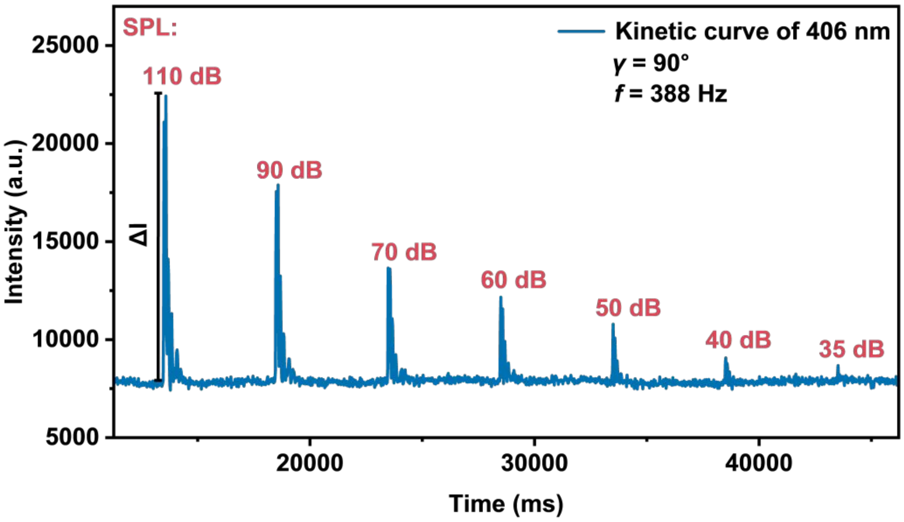

Figure S30. Optical response of the HOF-TCPB@SF acoustic sensor under the sound (f = 388 Hz, γ = 90°) with different SPLs (110 dB – 35 dB).

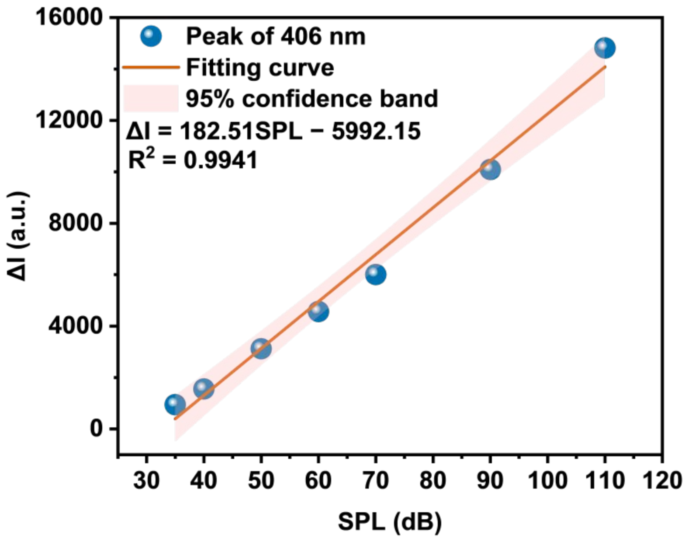

Figure S31. The linear relationship between the fluorescence intensity variation (ΔI) of HOF-TCPB@SF and SPL.

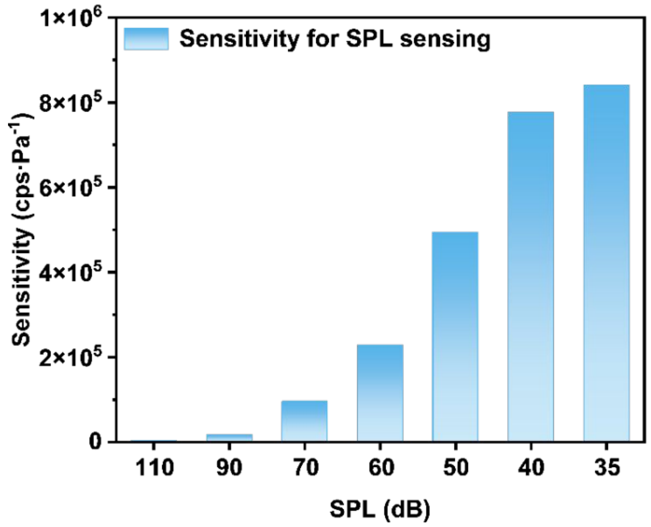

Figure S32. Histogram of sensitivity for HOF-TCPB@SF on SPL = 110 dB, 90 dB, 70 dB, 60 dB, 50 dB, 40 dB, and 35 dB.

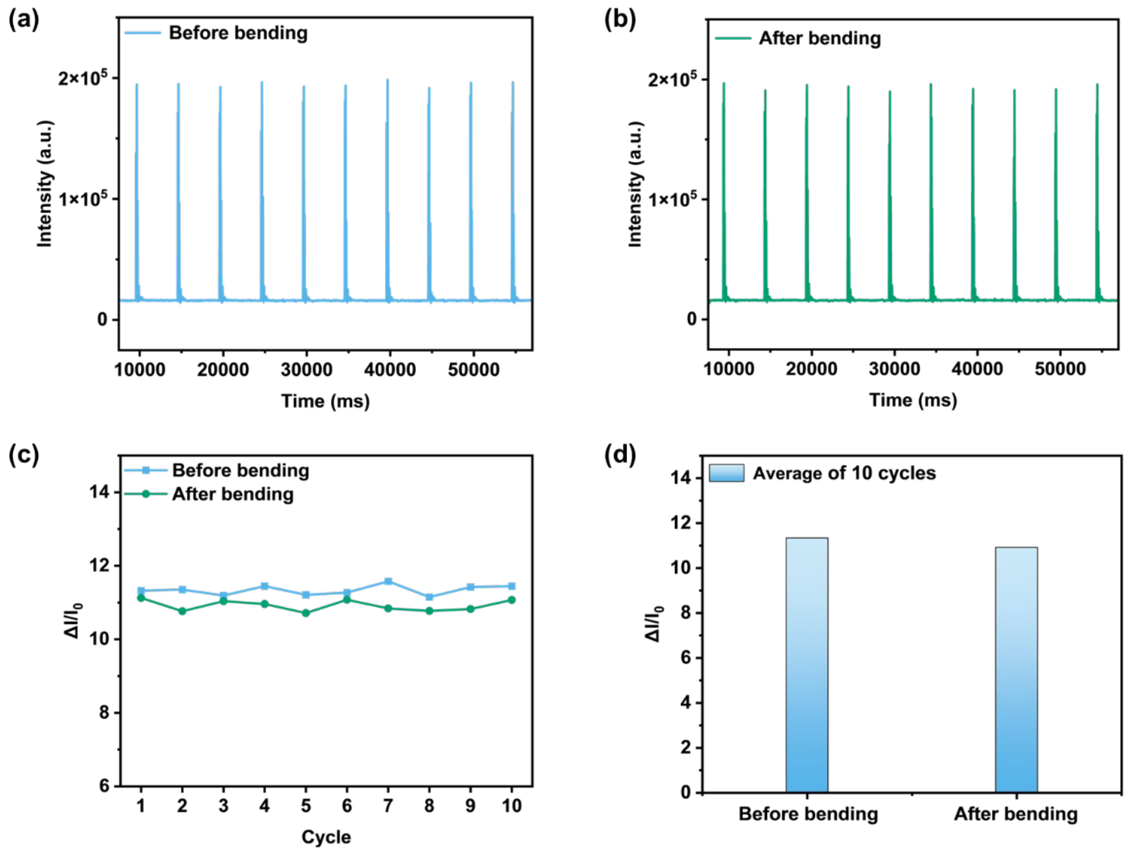

Figure S33. Optical response signal at 406 nm of the HOF-TCPB@SF acoustic sensor to the sound (γ = 90°, SPL = 110 dB) for 10 cycles (a) before bending, and (b) after bending. (c) Line chart of fluorescence intensity variation ($\Delta I/I_0$) of the HOF-TCPB@SF acoustic sensor to the sound (γ = 90°, SPL = 110 dB) before and after bending. (d) Average of fluorescence intensity variation ($\Delta I/I_0$) before and after bending.

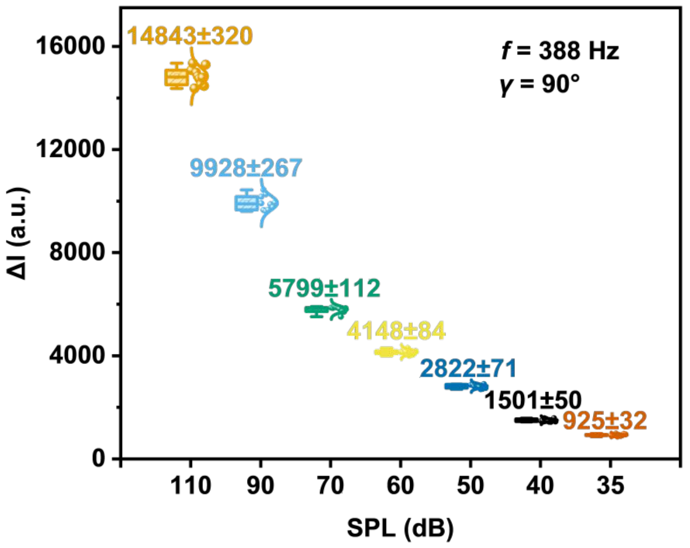

Figure S34. The boxplot of fluorescence response signals for the sound (f = 388 Hz, γ = 90°) of 110 dB, 90 dB, 70 dB, 60 dB, 50 dB, 40 dB, and 35 dB under 10 parallel measurements.

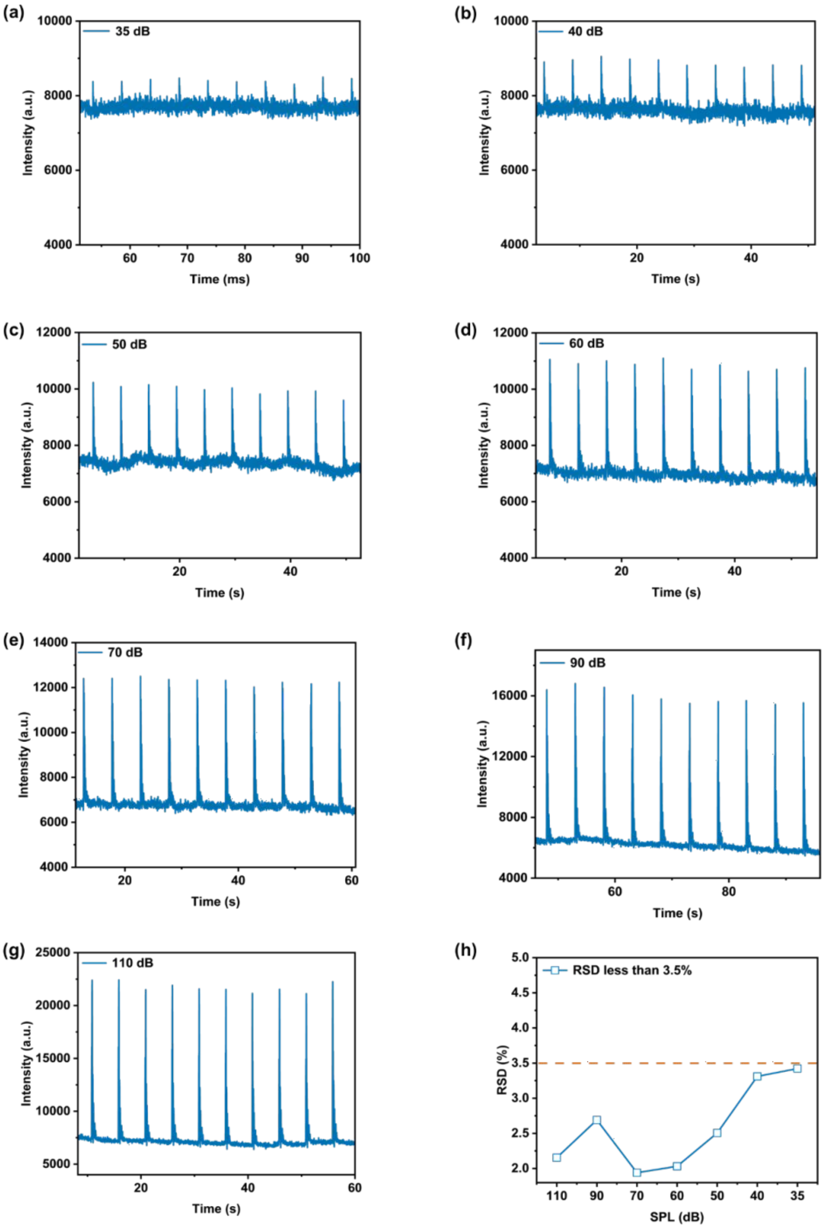

Figure S35. 10 cycles repeated measurements of (a) 35 dB, (d) 40 dB, (c) 50 dB, (d) 60 dB, (e) 70 dB, (f) 90 dB, and (g) 110 dB utilized for the calculation of RSD. (h) The RSD variation of HOF-TCPB@SF in sound fields with different SPLs of 110 – 35 dB.

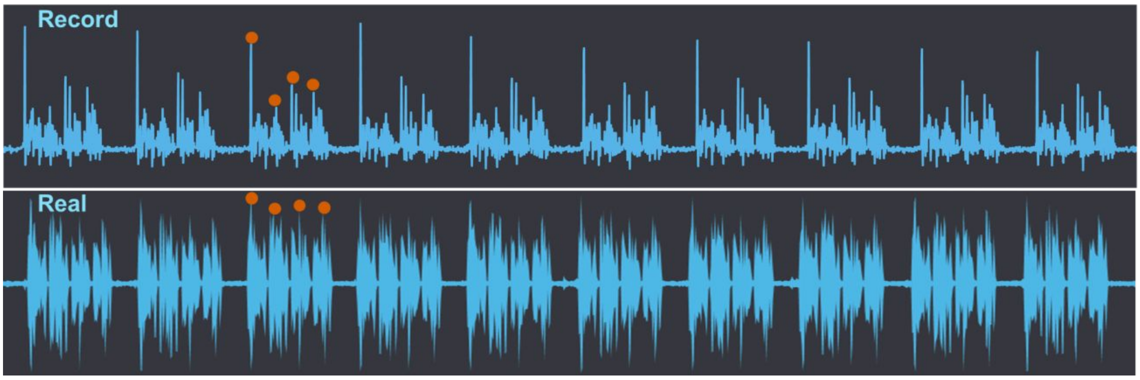

Figure S36. The comparison of real and recorded signal curve for the speech of a Chinese classical poetry《望庐山瀑布》with 10 cycles.

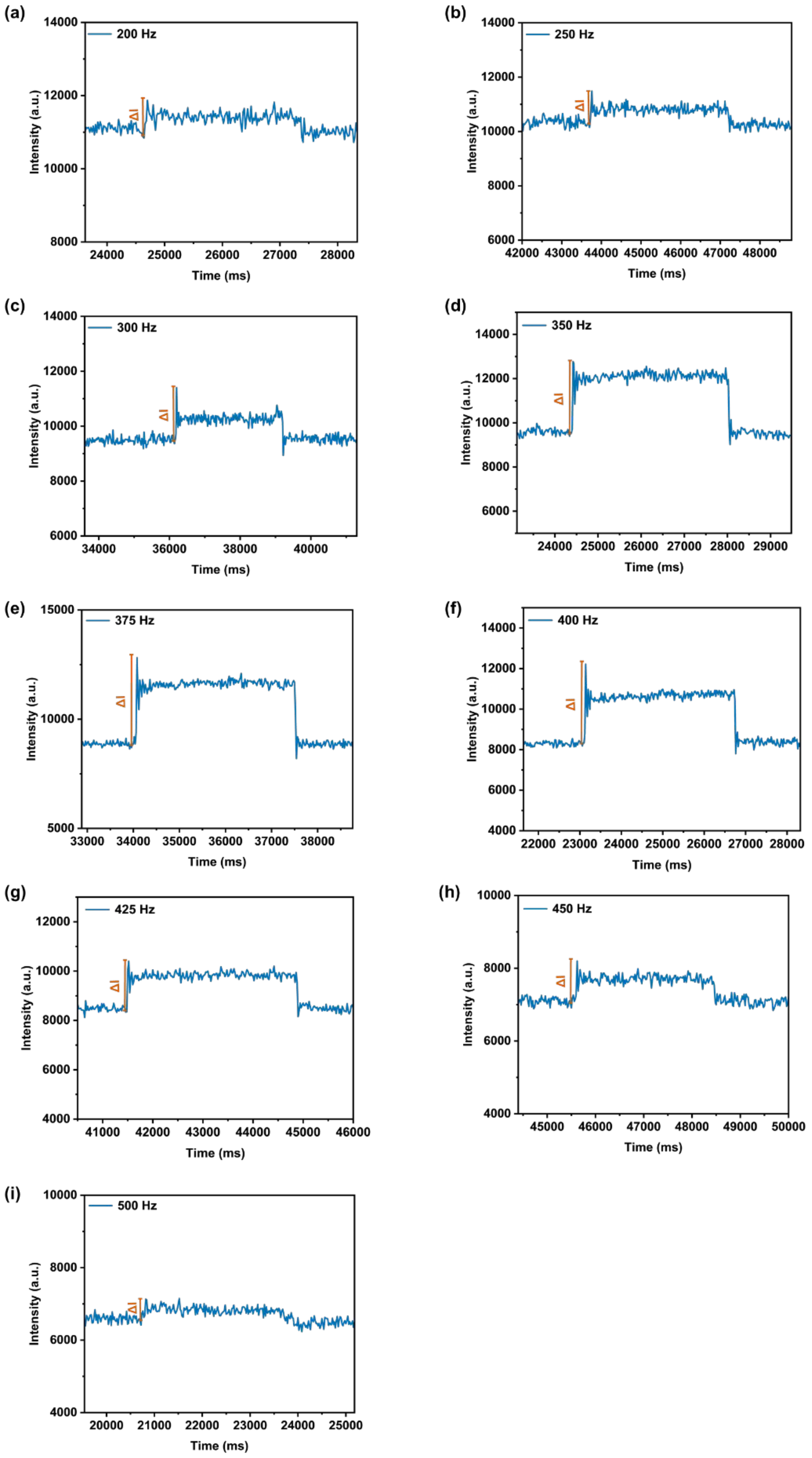

Figure S37. (a – i) The optical signal responses toward the sounds with different frequencies (200 – 500 Hz) for sound sensing.

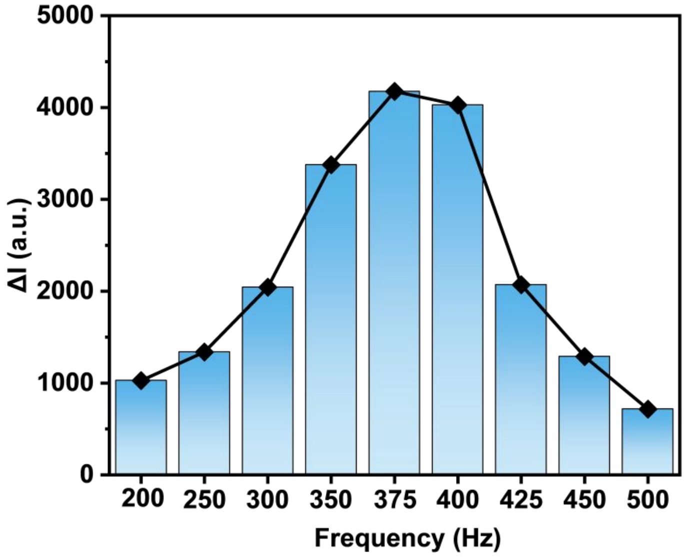

Figure S38. The dependence of ΔI on sound frequency of HOF-TCPB@SF acoustic sensor in sound sensing process.

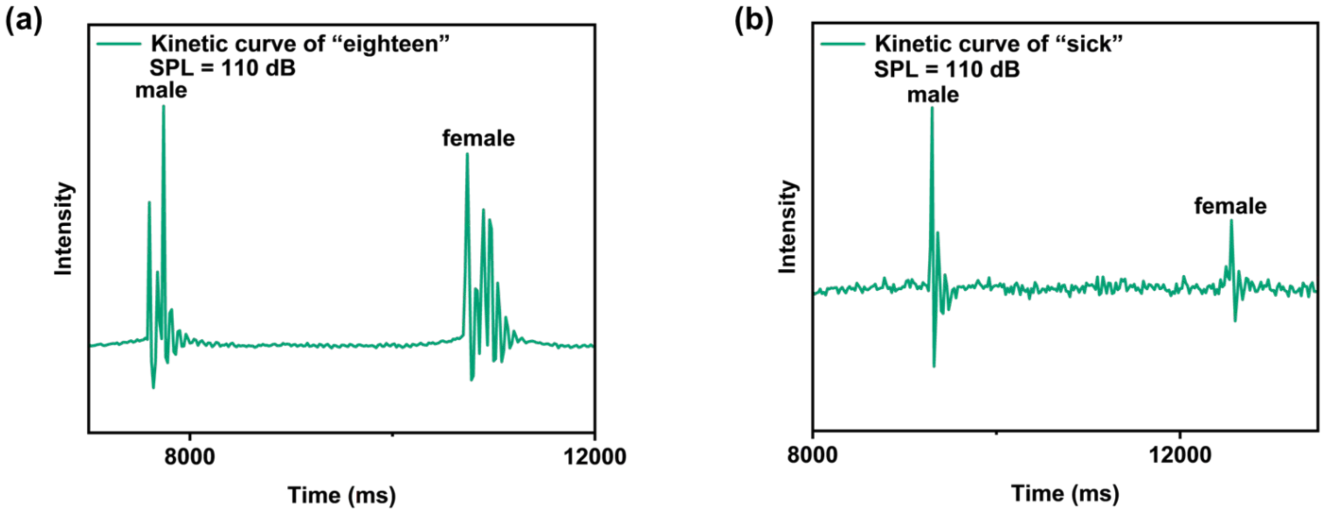

Figure S39. Recognition signals of the word (a) “eighteen”, (b) “sick” pronounced by male and female, respectively.

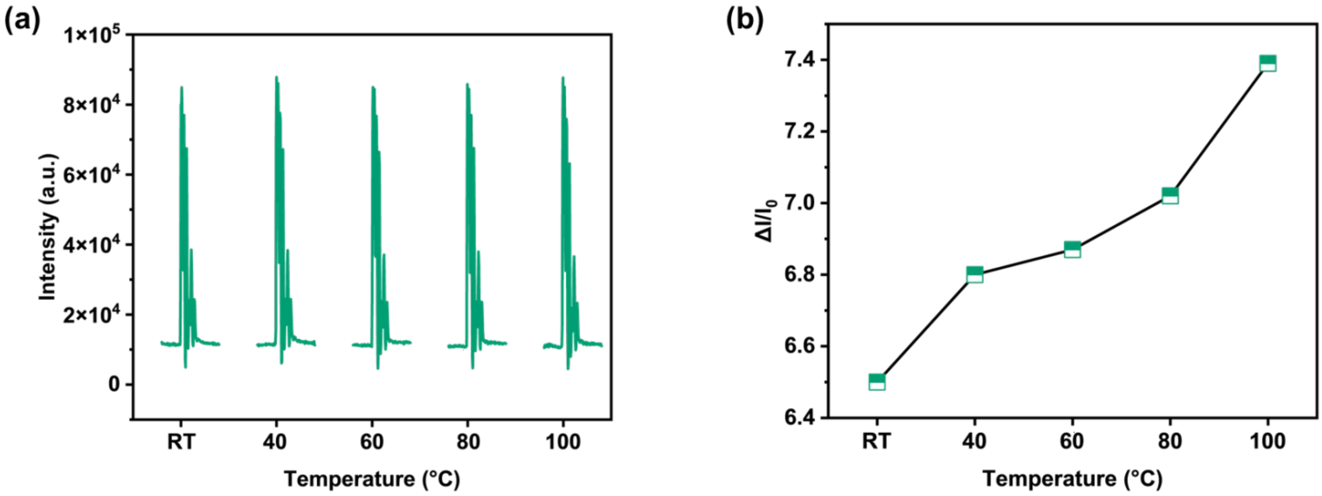

Figure S40. (a) Optical response signal at 406 nm of the HOF-TCPB@SF acoustic sensor to the sound (SPL = 110 dB) at temperatures of room temperature (RT, 22 °C), 40 °C, 60 °C, 80 °C, and 100 °C, respectively. (b) The relationship between relative fluorescence intensity variation ($\Delta I/I_0$) and temperature.

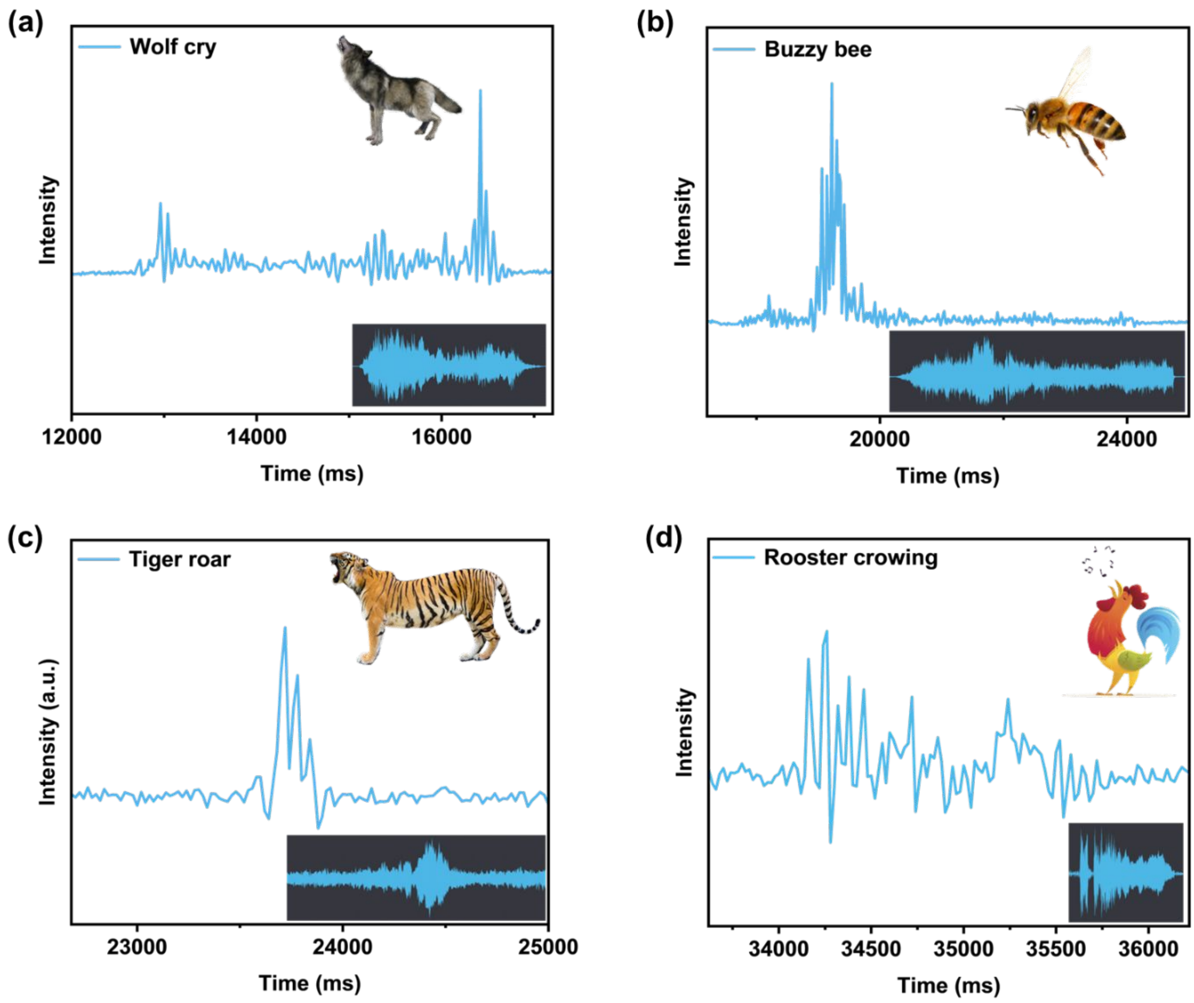

Figure S41. Discrimination of animal sound recordings including (a) wolf, (b) bee, (c) tiger, and (d) rooster.

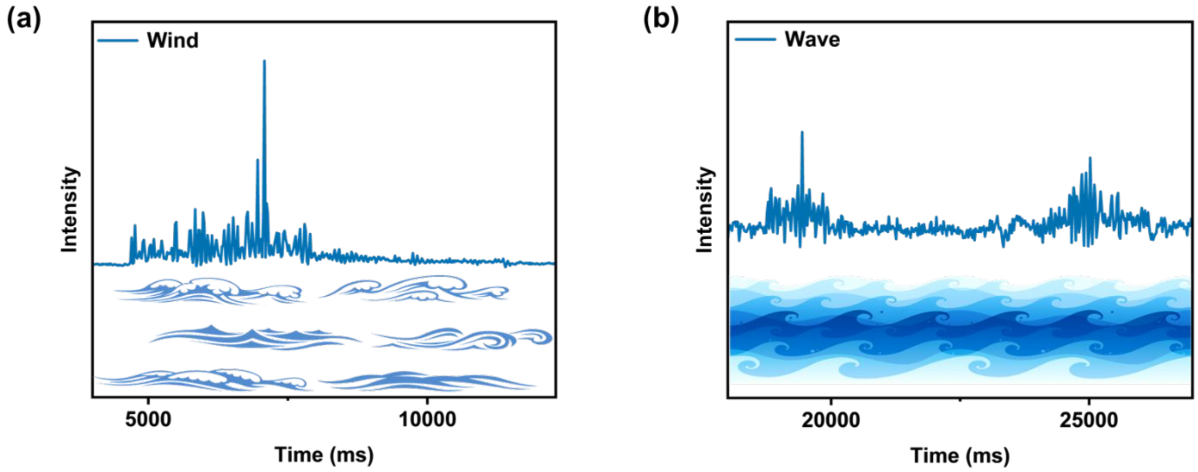

Figure S42. Discrimination of geophysical sound recordings including (a) wind and (b) wave.

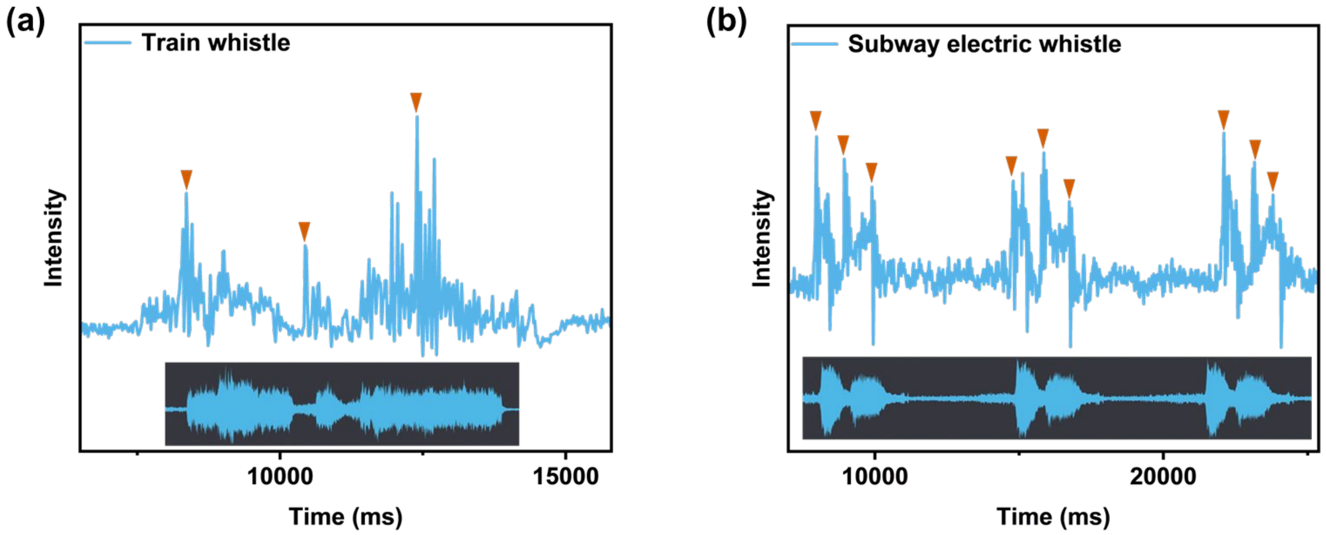

Figure S43. Discrimination of traffic noise recordings including (a) train whistle and (b) subway electric whistle.

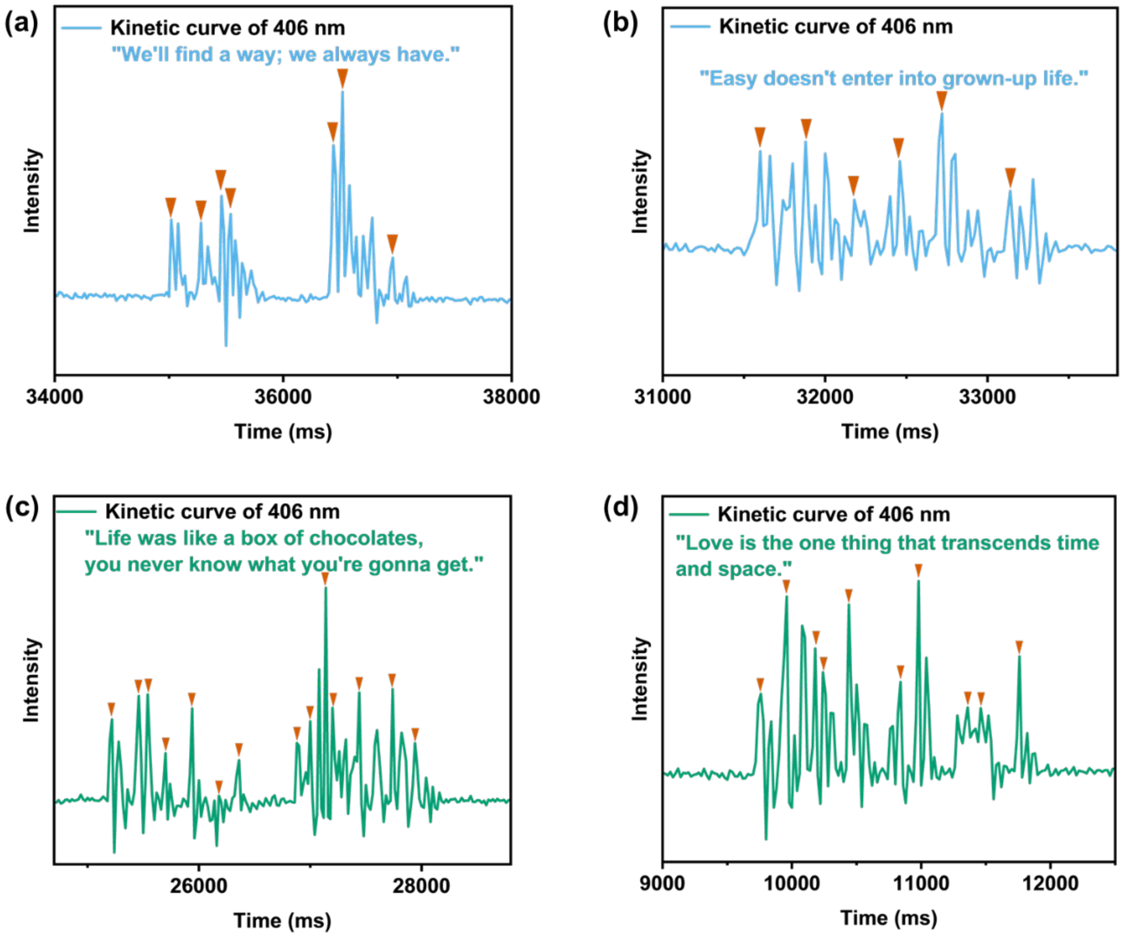

Figure S44. Recognition signals of different English movie lines.

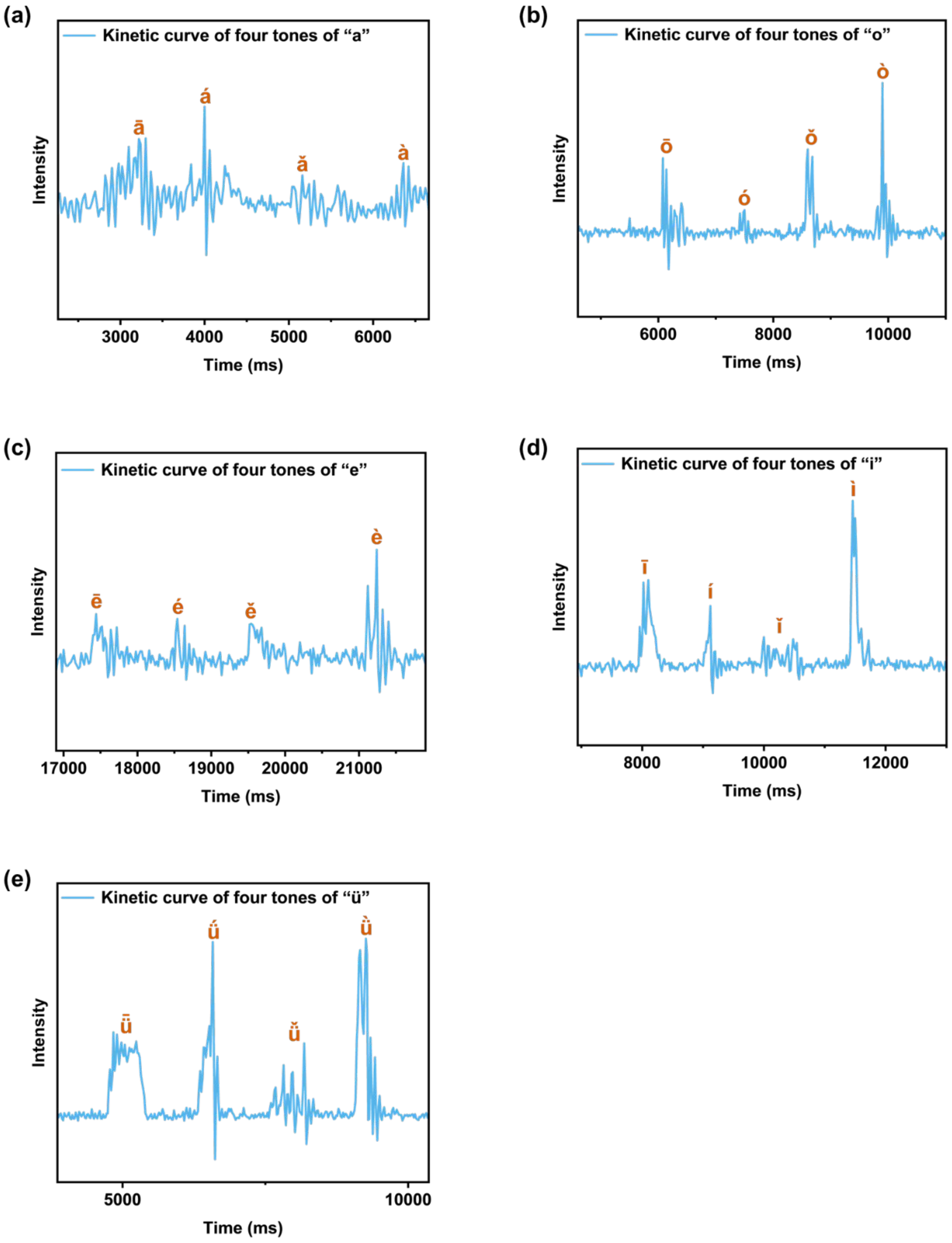

Figure S45. Optical signal responses toward the sounds of four tones of (a) “a”, (b) “o”, (c) “e”, (d) “i”, and (e) “ü”.

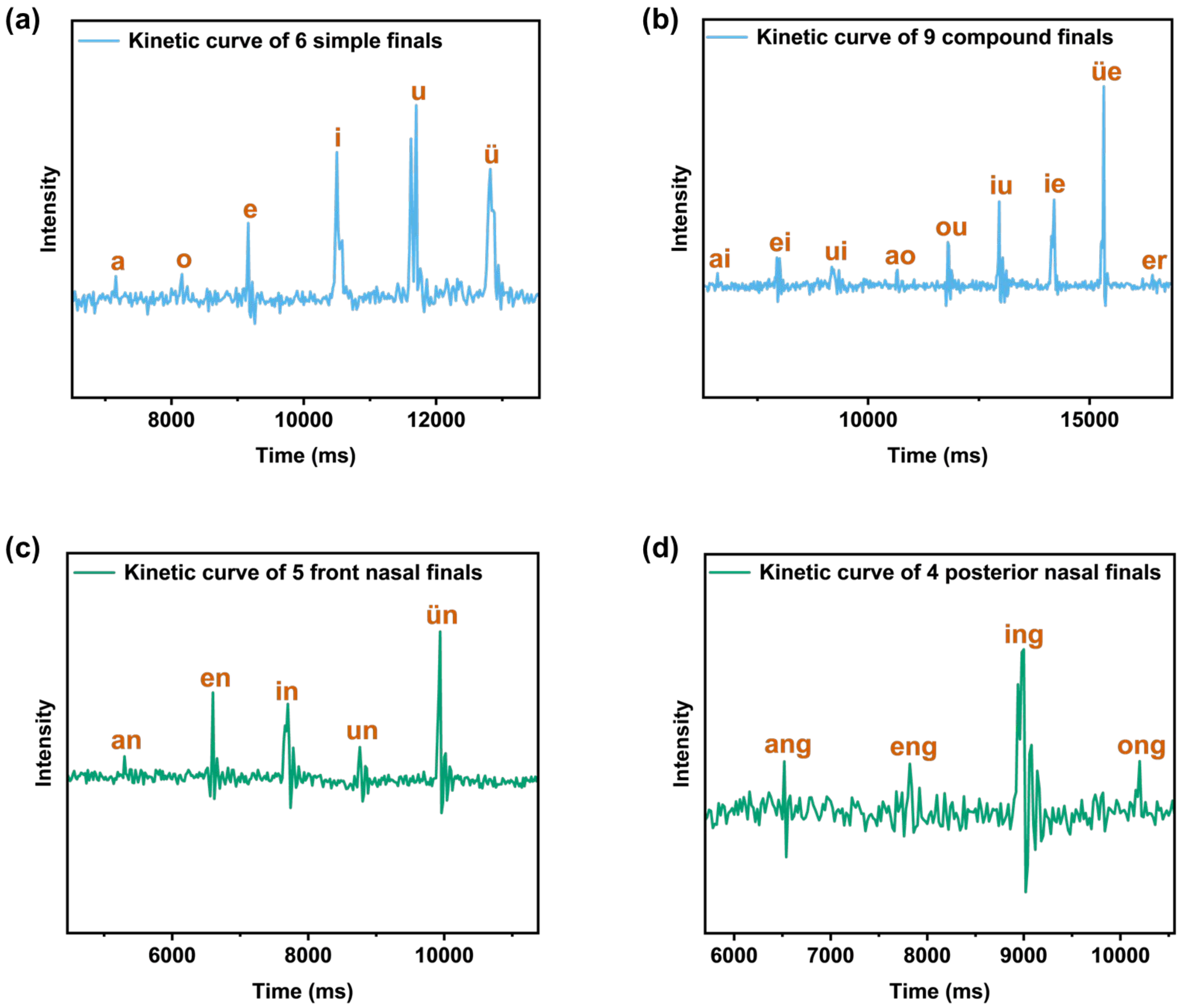

Figure S46. Optical signal responses toward the sounds of 24 finals including (a) 6 simple finals (b) 9 compound finals, (c) front nasal finals, and (d) 4 posterior nasal finals.

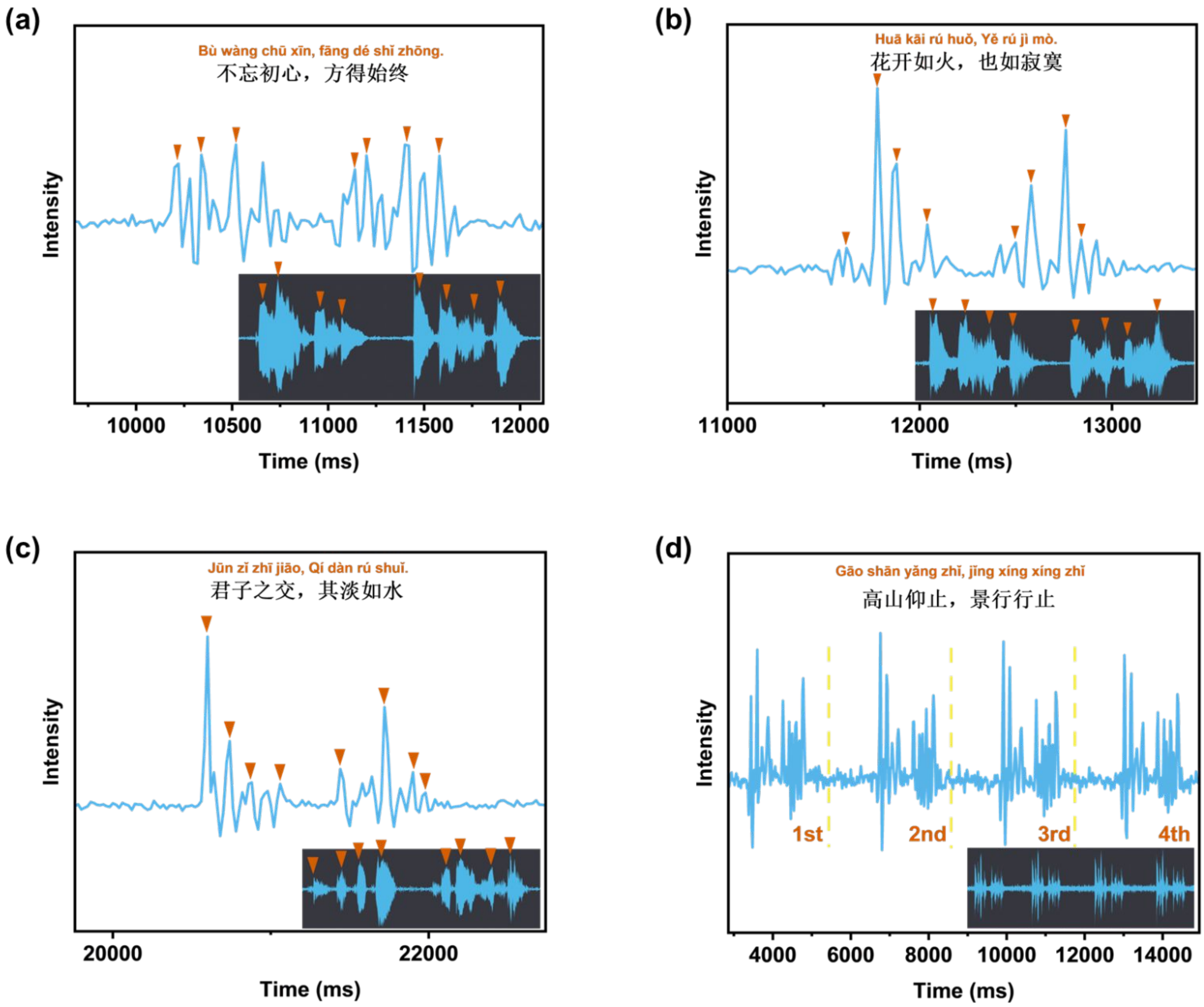

Figure S47. Kinetic curve of the sound of Chinese eight-character epigrams.

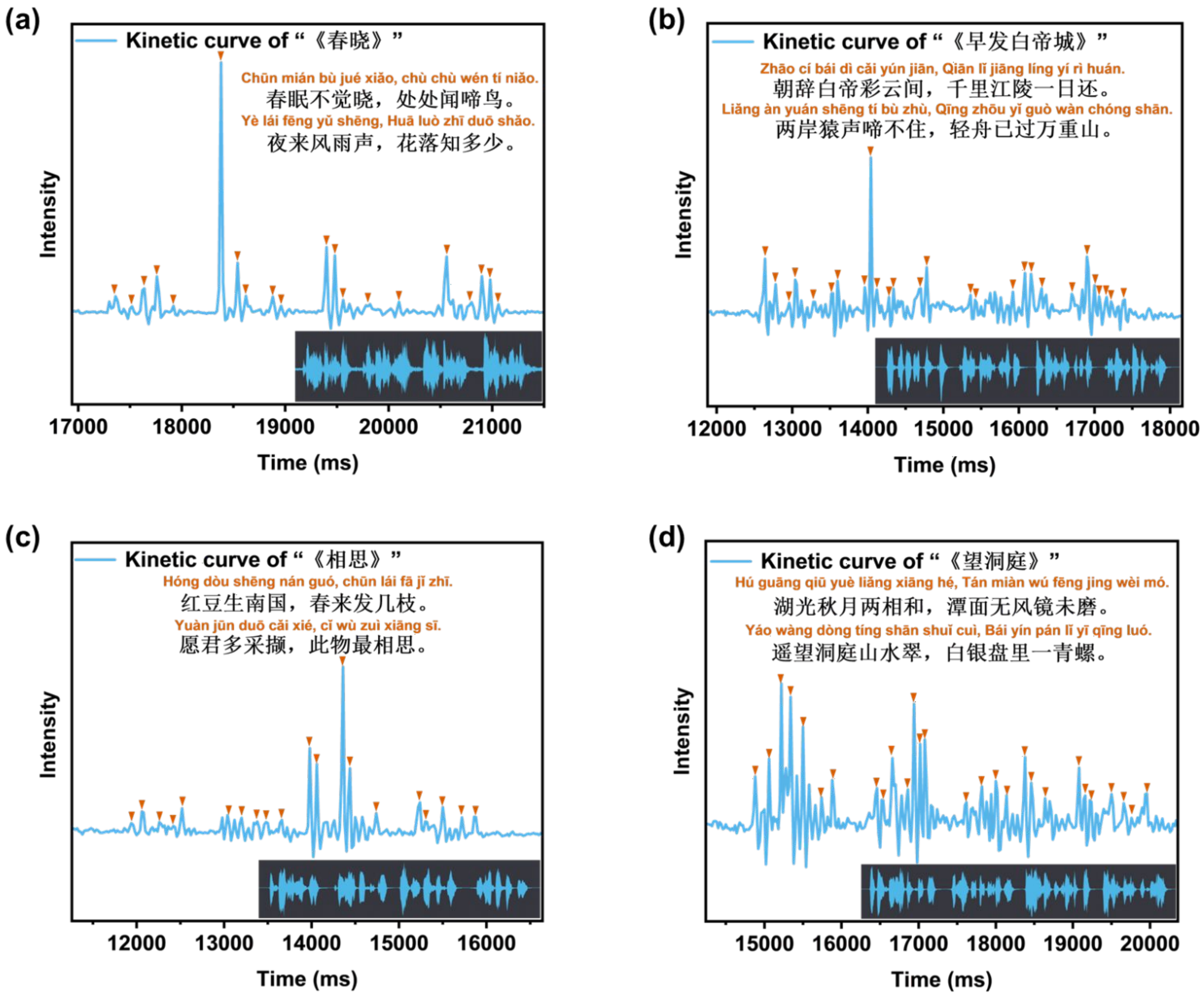

Figure S48. Kinetic curve of the sound of landscape poetries such as (a) “《春晓》”, (b) “《早发白帝城》”, (c) “《相思》”, (d) “《望洞庭》”.

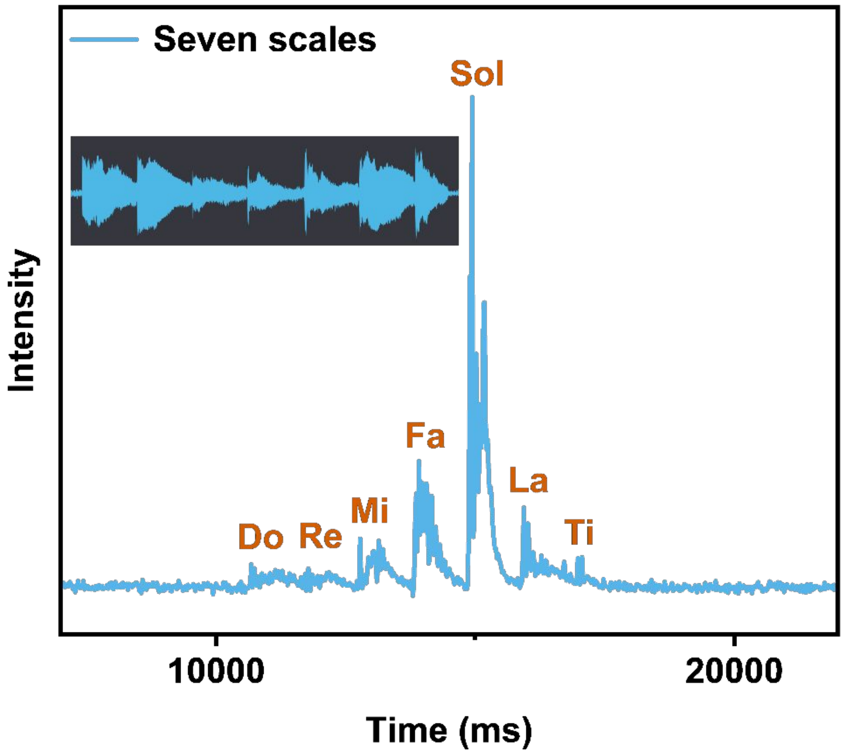

Figure S49. Optical signal variation of HOF-TCPB@SF acoustic sensor to the sound of seven different musical notes (“Do”, “Re”, “Mi”, “Fa”, “So”, “La”, and “Ti”).

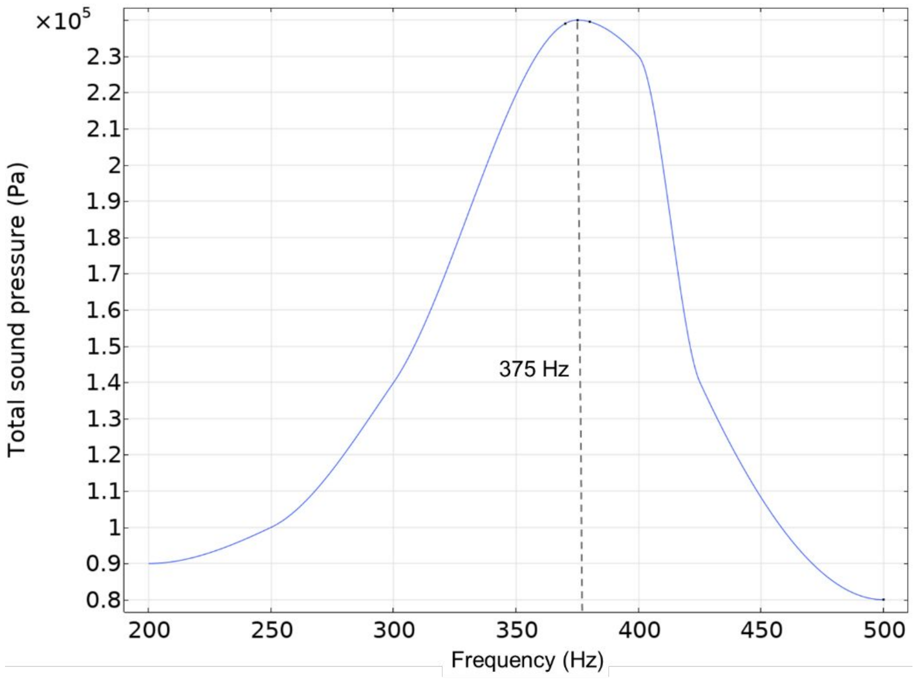

Figure S50. Dependence of total sound pressure on sound frequency obtained by FES.

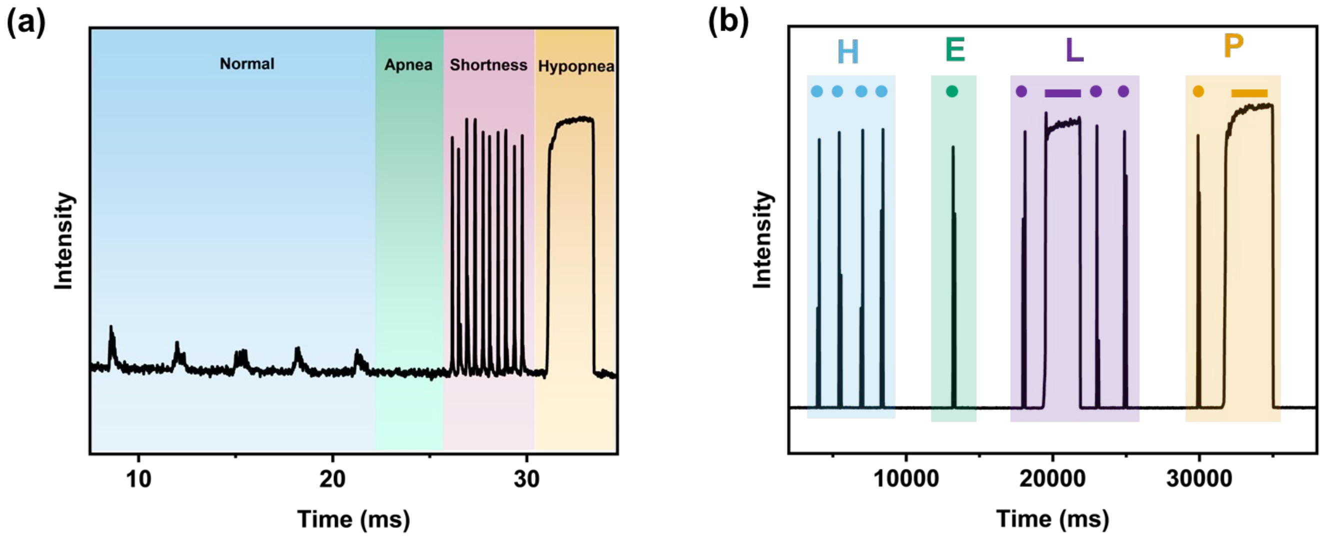

Figure S51. (a) Breath monitoring for the detection of respiratory states including normal, apnea, shortness, and hypopnea. (b) Communication for quadriplegic aphasia to convey "HELP" information.

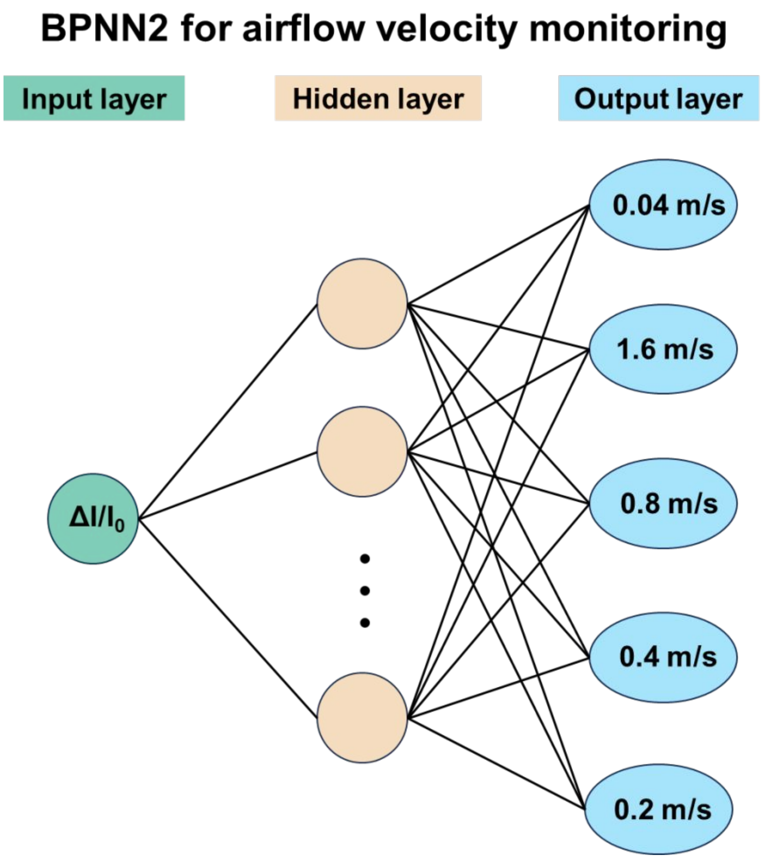

Figure S52. (a) BPNN2 for airflow velocity monitoring.

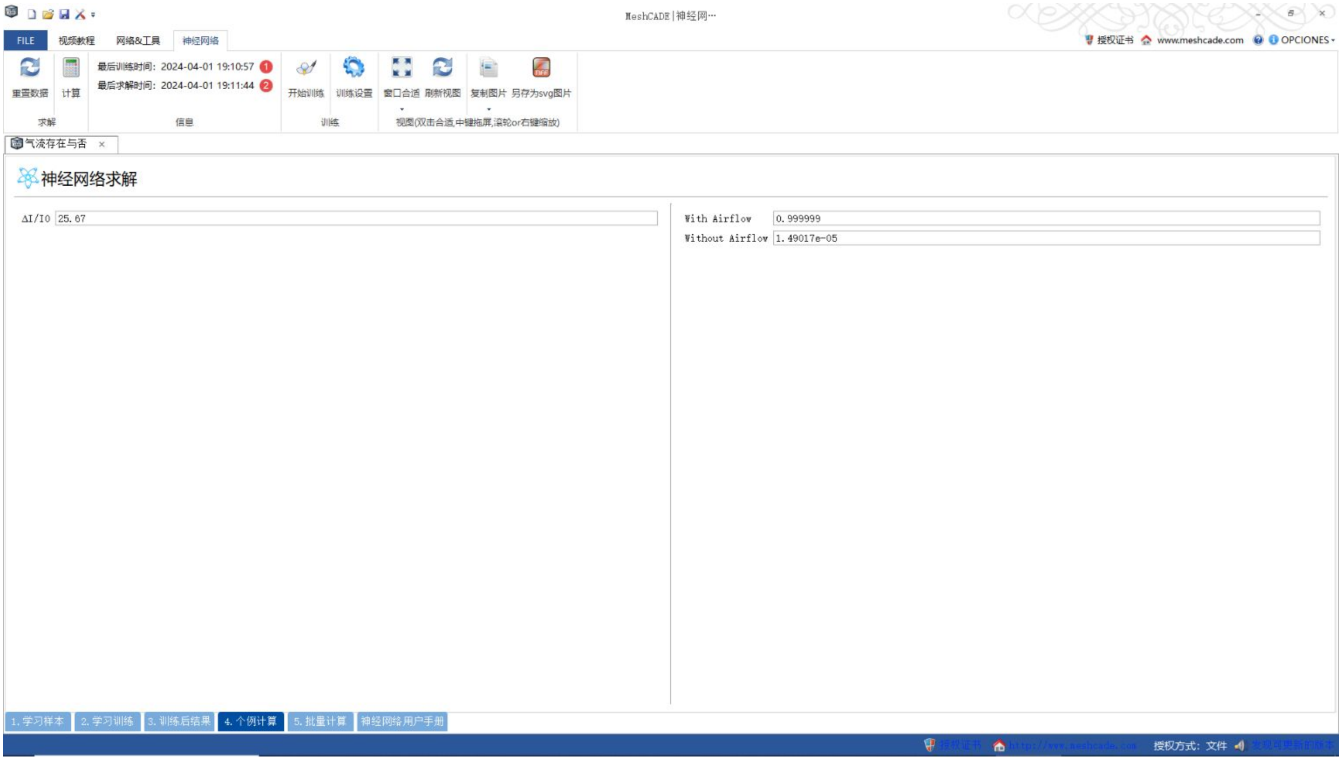

Figure S53. The human-computer interaction by BPNN1 for the real-time detection of whether the presence of airflow in maishishenjingwangluo software.

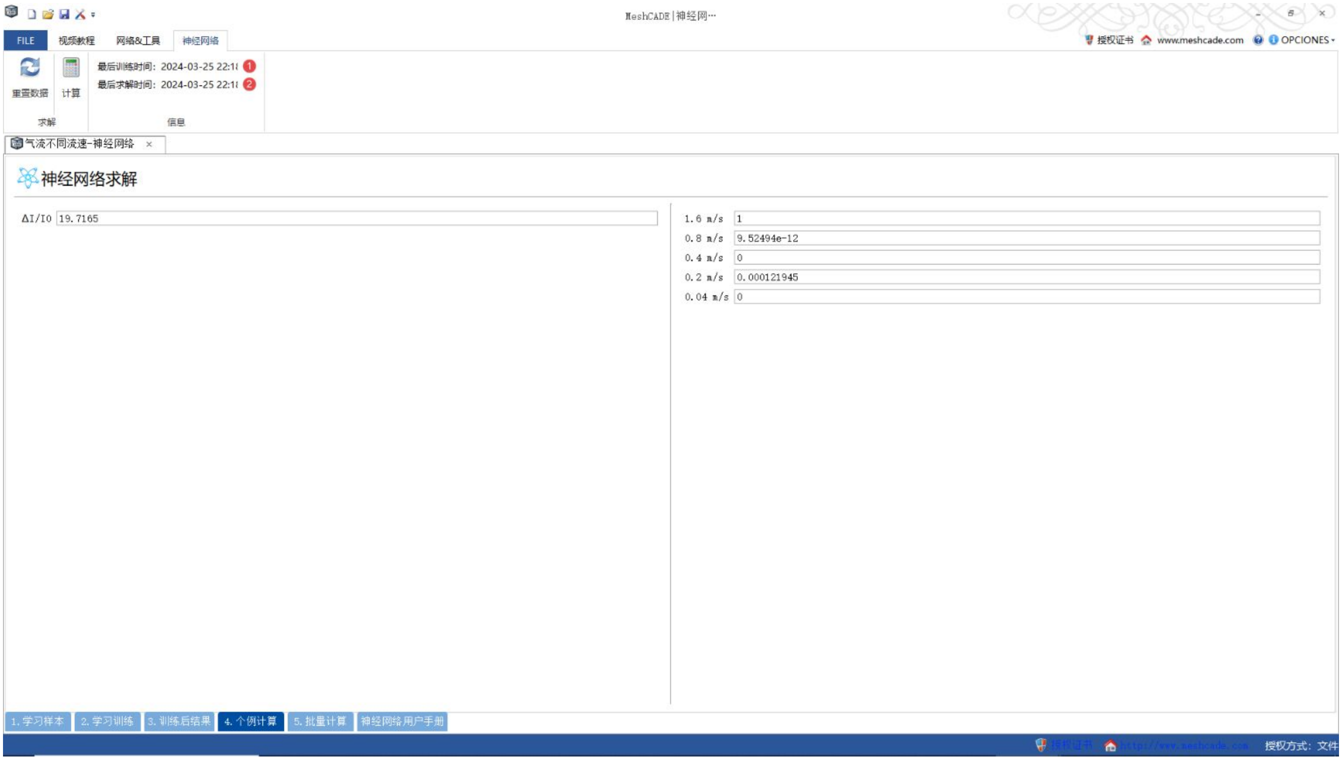

Figure S54. The human-computer interaction by BPNN2 for the real-time detection of different airflow velocities in maishishenjingwangluo software.

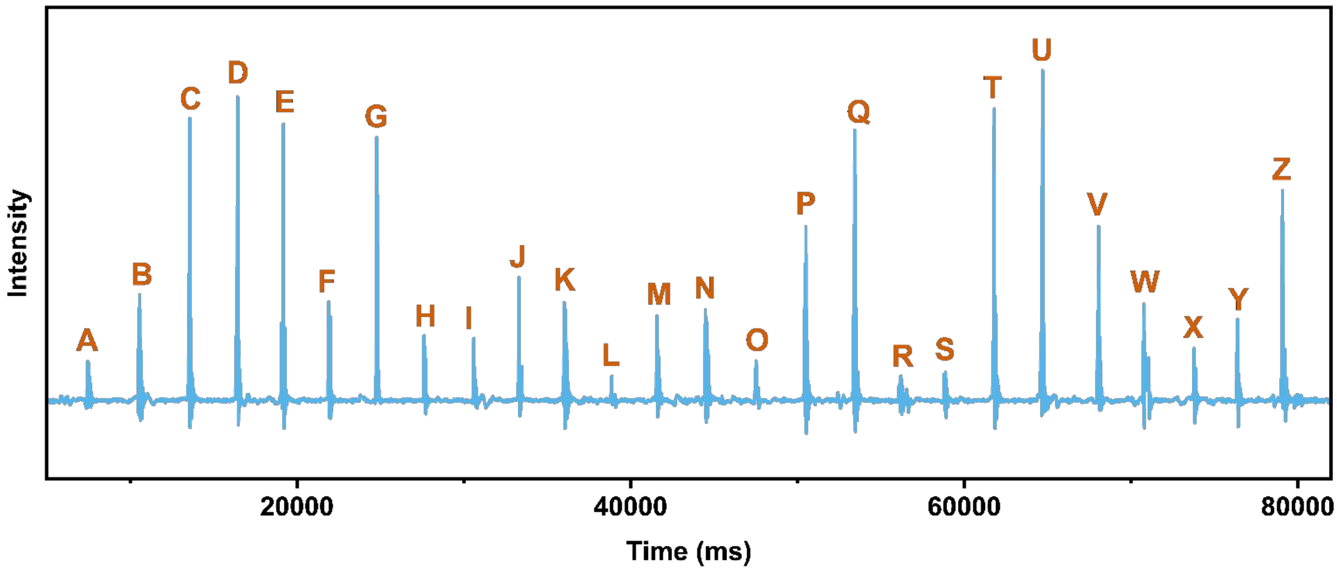

Figure S55. The recorded optical signal response of 26 English letters.

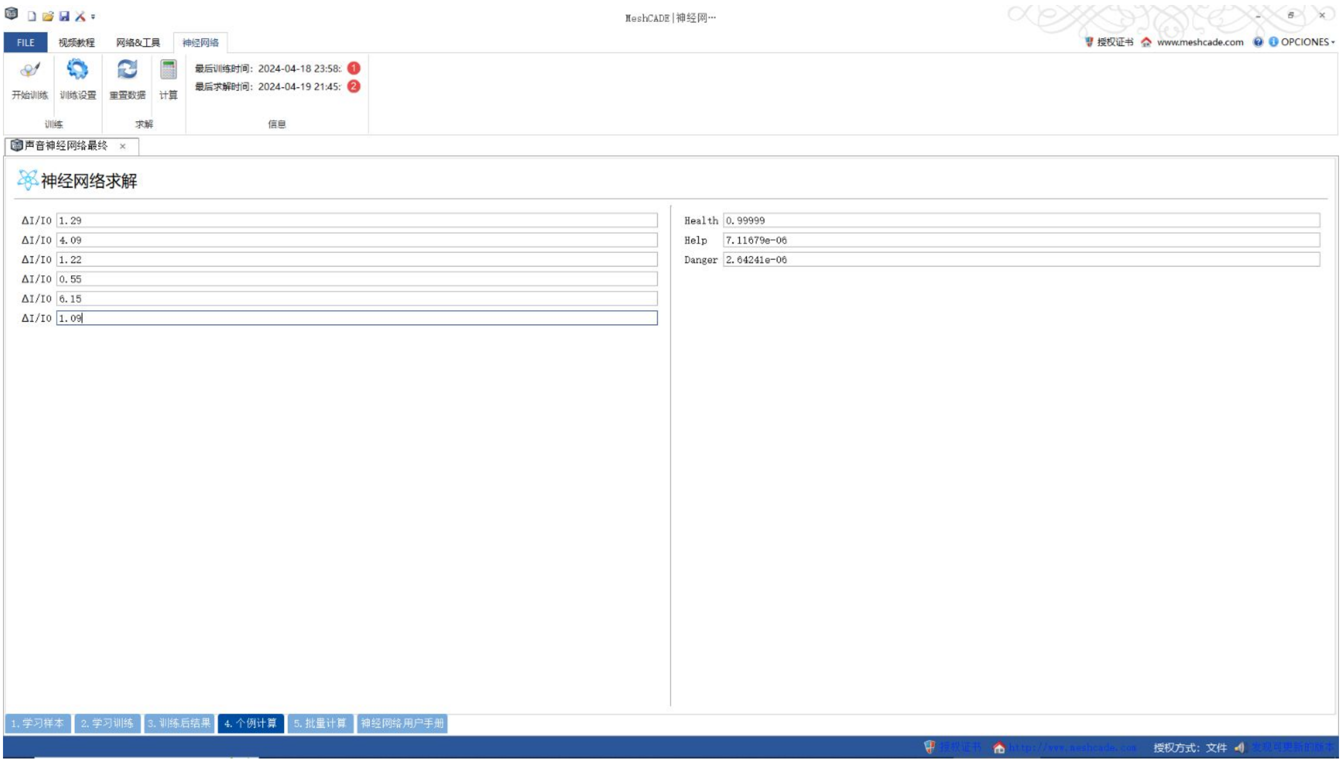

Figure S56. The human-computer interaction by BPNN3 for the real-time detection of speech information such as "health", "help", "danger" in maishishenjingwangluo software.

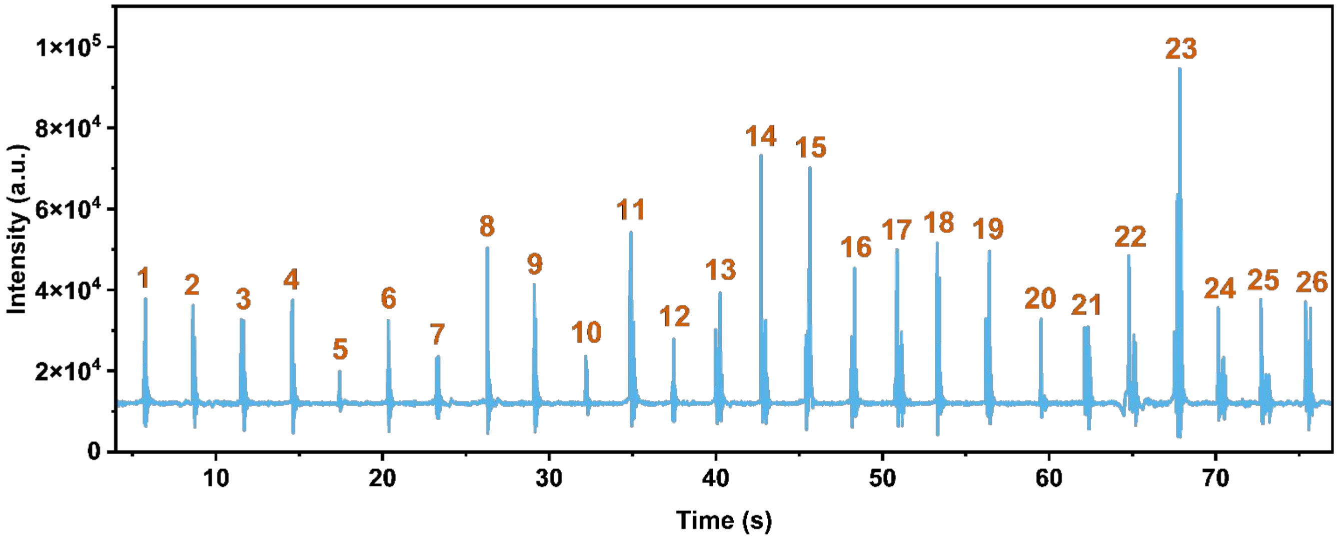

Figure S57. The recorded optical signal responses of English pronunciation of numbers 1 – 26.

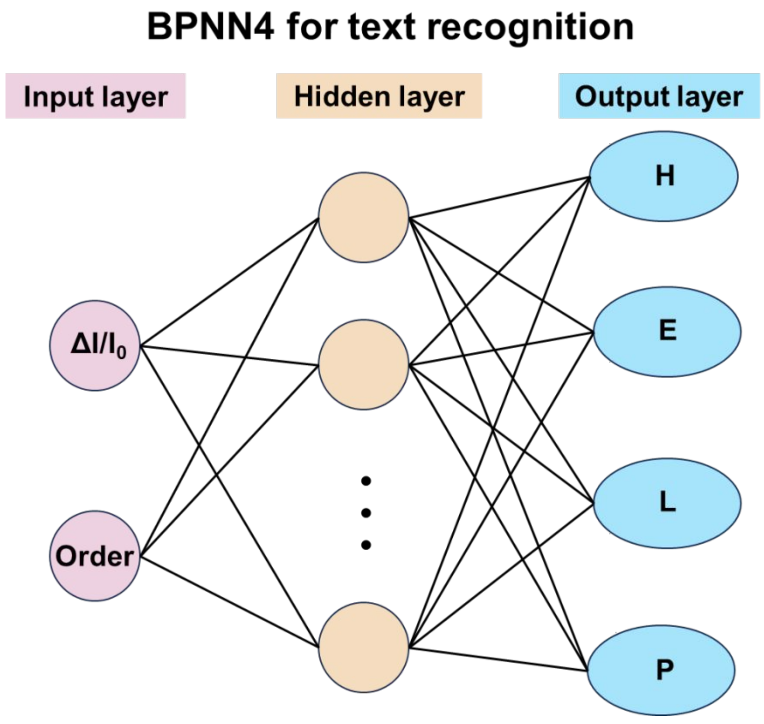

Figure S58. BPNN4 for text recognition.

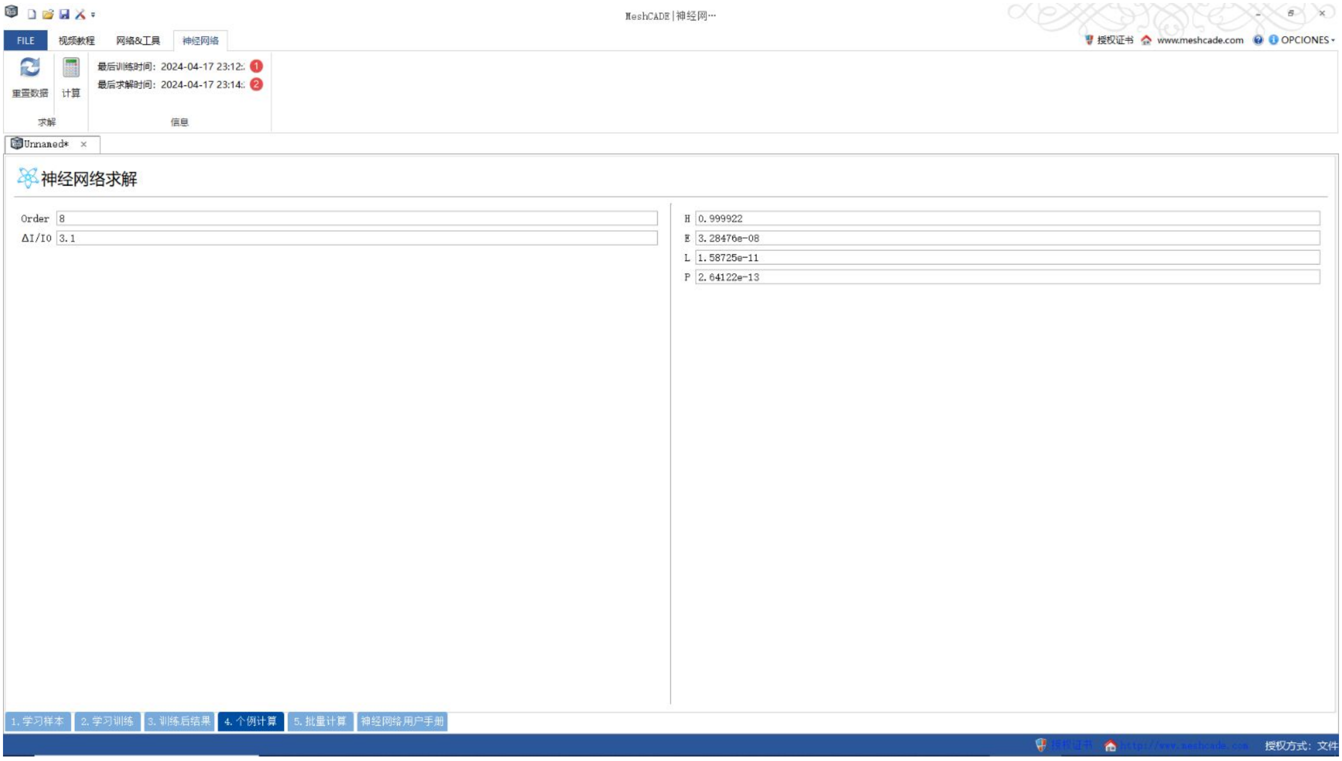

Figure S59. The human-computer interaction by BPNN4 for the real-time detection of different text information in maishishenjingwangluo software.

Table S1. Weight percentage and atomic percentage of C, N and O Elements in EDX energy spectrum of SF and HOF-TCPB@SF.

Element	Weight percentage (%)		Atomic percentage (%)	
	SF	HOF-TCPB@SF	SF	HOF-TCPB@SF
C	53.79	57.91	59.49	63.70
N	18.08	13.13	17.14	12.38
O	36.55	28.97	23.36	23.92
Total	100	100	100	100

Table S2. The performance comparison of HOF-TCPB@SF airflow sensor with other airflow sensors.

Materials	Principle	Sensitivity	Response time	Detection range	Detection limit	Ref.
HOF-TCPB@SF	Optical	12.48 m^{-1} s	0.04 s	0.04 – 1.6 m s^{-1}	0.0076 m s^{-1}	This work
CNTs/CSF	Piezoresistance	4% in 3 m s^{-1}	1.3 s	0.05 – 3.5 m s^{-1}	0.05 m s^{-1}	[1]
RGO	Electrical	32-50% in 7.2 m s^{-1}	26 s	0.026 – 7.2 m s^{-1}	0.026 m s^{-1}	[2]
CNT/PDMS	Piezoresistance	N/A	0.5 s	1 – 5.5 m s^{-1}	1 m s^{-1}	[3]
Pt wires	Hot wire	N/A	0.1s	0.3 – 6 m s^{-1}	0.3 m s^{-1}	[4]
Al and Pt wires	Hot wire	N/A	0.12 s	0 – 5 m s^{-1}	0.028 m s^{-1}	[5]
PVDF	Piezoresistance	N/A	7.565 s	2.5 – 14 m s^{-1}	2.5 m s^{-1}	[6]
h-rGO	Optical	6.2 × 10^7 mV/RIU	0.5 s	3.5 – 35 mm s^{-1}	0.0035 m s^{-1}	[7]
SCNTNs	Piezoresistance	0.0124 s m^{-1}	0.021 s	0.11 – 5.51 m s^{-1}	0.11 m s^{-1}	[8]
GSEM	Electrical	9.12% s m^{-1} (0.0176 – 0.1 m s^{-1}) and 0.2% m^{-1} s (0.1 – 3.5 m s^{-1})	1.04 s	0.0176 – 3.5 m s^{-1}	0.0176 m s^{-1}	[9]
PEDOT: PSS-Cu^{2+} fibers	Electrical	N/A	0.43 s	0.5 – 3.5 m·s^{-1}	0.32 m·s^{-1}	[10]

CFs-PVA	Electrical	24.7% s m^{-1} (1 – 8 m s^{-1}) and 90.4% s m^{-1} (8 – 16 m s^{-1})	0.103 s	0.068 – 16 m s^{-1}	0.068 m s^{-1}	[11]
GCEF	Piezoresistance	44.59% – 0.82% s m^{-1} (0.0087–1.5 m s^{-1}) and −4.3% s m^{-1} (2 – 13 m s^{-1})	0.1 s	0.0087 – 23 m s^{-1}	0.0087 m s^{-1}	[12]
micro-TED	Electrical	N/A	1.7 s	20 – 100 Ml min^{-1}	0.004 m s^{-1}	[13]
SLIGF	Piezoresistance	0.1131 s m^{-1} (0.0023 – 0.471 m s^{-1}) and 0.00873 s m^{-1} (0.5 m s^{-1} – 2.35 m s^{-1})	0.5 s	0.0023 – 2.35 m s^{-1}	0.0023 m s^{-1}	[14]
SiNW-BHS	Piezoresistance	N/A	0.04 s	0.15 – 15.3 m s^{-1}	0.15 m s^{-1}	[15]
Piezoelectric Fiber	Piezoelectric	N/A	0.57 s	0.5 – 10 m s^{-1}	0.5 m s^{-1}	[16]
silver film /PDMS	Piezoresistance	0.016 – 0.13 s m^{-1}	1 s	0.2 – 1.2 m s^{-1}	0.2 m s^{-1}	[17]

Table S3. The performance comparison of HOF-TCPB@SF acoustic sensor with other acoustic sensors.

Materials	Principle	SPL range (dB)	DL (dB)	Sensitivity	RT (s)	Ref.
HOF-TCPB@SF	Optical	35 – 110	0.2980	105140.77 cps Pa^{-1} cm^{-2}	0.04	This work
TENG–FEST	electric	50 – 105	N/A	129 mV dB^{-1}	N/A	[18]
MCPP ANG	electric	100 – 115	N/A	3.37 V Pa^{-1}	N/A	[19]
PMAS	electric	94	N/A	40 mV Pa^{-1} cm^{-2}	N/A	[20]
PTFE-PET film	electric	50 − 120	N/A	3.2 mV Pa^{-1}	N/A	[21]
V-AuNW	electric	Not available	N/A	0.48 – 4.26 Pa^{-1}	N/A	[22]
Capacitive diaphragm	electric	29 – 134	N/A	11.1 mV Pa^{-1} mm^{-2}	N/A	[23]
Bi nanosheets	electric	$\geq$ 45	N/A	42000 Pa^{-1}	N/A	[24]
P(VDF-TrFE) nanofiber mesh	electric	70 − 110	N/A	70 mV Pa^{-1}	N/A	[25]
PVDF nanofibres	electric	60 – 115	N/A	266 mV Pa^{-1}	N/A	[26]
PTFE-Al film	electric	10 – 110	N/A	9.54 V Pa^{-1}	N/A	[27]

PVDF-TrFE/BTNP	electric	33 − 135	N/A	607 mV Pa^{-1}	N/A	[28]
PZT film	electric	94	N/A	> 45 mV·Pa^{-1}	N/A	[29]

Table S4. Summary of input and output information during the training for BPNN1.

Input	Output	
$\Delta I/I_0$	With airflow	Without airflow
25.67	1	0
0	0	1

In Table S4, all data is applied to train for the BPNN1. The relative fluorescence intensity variation ($\Delta I/I_0$) was inputted into BPNN1. With or without airflow ("1" or "0", respectively) as output information was outputted from BPNN1.

Table S5. Network structure information for BPNN1.

**Network structure information**				
Input layer	1 neuron	1 Paranoid		
Hidden layer 1	6 neurons	1 Paranoid		
Output layer	2 neurons			
Network type	FANN_NETTYPE_LAYER			
Training function	FANN_TRAIN_RPROP			
Error function	FANN_ERRORFUNC_LINEAR			
Termination function	FANN_STOPFUNC_MSE			
Hidden layer excitation function	FANN_SIGMOID_SYMMETRIC			
Output layer excitation function	FANN_SIGMOID_SYMMETRIC			
**Network weight value**				
Arrangement	Wire number	Output point (n)	Input point (m)	Weight value (W)
	0	0	2	0.17481
	1	1	2	-3.07314
	2	0	3	1.37
	3	1	3	1.99805
	4	0	4	-4.58466
**1**	5	1	4	-1.94224
	6	0	5	-4.693
	7	1	5	0.32988
	8	0	6	2.20701
	9	1	6	0.508405
	10	0	7	2.95302
	11	1	7	0.0742649
	12	2	9	2.2639
	13	3	9	0.0402846
	14	4	9	1.38753
	15	5	9	-4.28567
**2**	16	6	9	6.65358
	17	7	9	5.95632
	18	8	9	2.03433
	19	2	10	1.16045
	20	3	10	-0.341016

21	4	10	2.83896
22	5	10	2.32024
23	6	10	-3.59839
24	7	10	-4.23501
25	8	10	2.06291

**Input / output column coefficients for manual calculation**		
Listing	Minimum	Maximum
$\Delta I/I_0$	0	27.0211
With airflow	0	1
Without airflow	0	1

**Deviation statistics: mean variance**		
Listing	All rows	Calculation line
With airflow	3.625e-12	3.625e-12
Without airflow	1.37e-10	1.37e-10

Table S6. The summary of mean square error (MSE), original value (OV), calculated value (CV), variance (Var) for BPNN1.

Input item	With airflow (MSE = 3.625e-12)			Without airflow (MSE = 1.37e-10)		
$\Delta I/I_0$	OV	CV	Var	OV	CV	Var
25.67	1	0.999999	1e-12	0	1.5e-05	2.25e-10
0	0	2.5e-06	6.25e-12	1	0.999993	4.9e-11

Table S7. The MATLAB code of the BPNN1.

MATLAB code

```
function [fO0, fO1] = MPredict(fI0)
    fI0 = (fI0 - (0 + 27.0210526315789501 ) / 2.0) /(27.0210526315789501 - (0 + 27.0210526315789501 ) / 2.0);
    fWei0 = 0.17481;
    fWei1 = -3.07314;
    fWei2 = 1.37;
    fWei3 = 1.99805;
    fWei4 = -4.58466;
    fWei5 = -1.94224;
    fWei6 = -4.693;
    fWei7 = 0.32988;
    fWei8 = 2.20701;
    fWei9 = 0.508405;
    fWei10 = 2.95302;
    fWei11 = 0.0742649;
    fWei12 = 2.2639;
    fWei13 = 0.0402846;
    fWei14 = 1.38753;
    fWei15 = -4.28567;
    fWei16 = 6.65358;
    fWei17 = 5.95632;
```

```
fWei18 = 2.03433;
fWei19 = 1.16045;
fWei20 = -0.341016;
fWei21 = 2.83896;
fWei22 = 2.32024;
fWei23 = -3.59839;
fWei24 = -4.23501;
fWei25 = 2.06291;
f0 = fI0;
f1 = 1.0;
f2 = 0.0;
f3 = 0.0;
f4 = 0.0;
f5 = 0.0;
f6 = 0.0;
f7 = 0.0;
f2 = f2 + f0 * fWei0;
f2 = f2 + f1 * fWei1;
f2 = f2 * 0.5;
f2 = (2.0 / (1.0 + exp(-2.0 * f2))- 1.0);
f3 = f3 + f0 * fWei2;
f3 = f3 + f1 * fWei3;
f3 = f3 * 0.5;
f3 = (2.0 / (1.0 + exp(-2.0 * f3))- 1.0);
f4 = f4 + f0 * fWei4;
f4 = f4 + f1 * fWei5;
f4 = f4 * 0.5;
f4 = (2.0 / (1.0 + exp(-2.0 * f4))- 1.0);
f5 = f5 + f0 * fWei6;
f5 = f5 + f1 * fWei7;
f5 = f5 * 0.5;
f5 = (2.0 / (1.0 + exp(-2.0 * f5))- 1.0);
f6 = f6 + f0 * fWei8;
f6 = f6 + f1 * fWei9;
f6 = f6 * 0.5;
f6 = (2.0 / (1.0 + exp(-2.0 * f6))- 1.0);
f7 = f7 + f0 * fWei10;
f7 = f7 + f1 * fWei11;
f7 = f7 * 0.5;
f7 = (2.0 / (1.0 + exp(-2.0 * f7))- 1.0);
f8 = 1.0;
f9 = 0.0;
f10 = 0.0;
f9 = f9 + f2 * fWei12;
f9 = f9 + f3 * fWei13;
```

```
f9 = f9 + f4 * fWei14;
f9 = f9 + f5 * fWei15;
f9 = f9 + f6 * fWei16;
f9 = f9 + f7 * fWei17;
f9 = f9 + f8 * fWei18;
f9 = f9 * 0.5;
f9 = (2.0 / (1.0 + exp(-2.0 * f9))- 1.0);
f10 = f10 + f2 * fWei19;
f10 = f10 + f3 * fWei20;
f10 = f10 + f4 * fWei21;
f10 = f10 + f5 * fWei22;
f10 = f10 + f6 * fWei23;
f10 = f10 + f7 * fWei24;
f10 = f10 + f8 * fWei25;
f10 = f10 * 0.5;
f10 = (2.0 / (1.0 + exp(-2.0 * f10))- 1.0);
fO0 = f9;
fO1 = f10;
fO0 = fO0 * (1 - (0 + 1 ) / 2.0) + (0 + 1) / 2.0;
fO1 = fO1 * (1 - (0 + 1 ) / 2.0) + (0 + 1) / 2.0;
```

Table S8. Summary of input and output information during the training for BPNN2.

Input	Output				
$\Delta I/I_0$	1.6 m s^{-1}	0.8 m s^{-1}	0.4 m s^{-1}	0.2 m s^{-1}	0.04 m s^{-1}
19.7165	1	0	0	0	0
9.7288	0	1	0	0	0
4.8651	0	0	1	0	0
2.0584	0	0	0	1	0
0.3467	0	0	0	0	1

In Table S8, all data is applied to train for the BPNN2. The relative fluorescence intensity variation ($\Delta I/I_0$) was inputted into BPNN2. The different airflow velocities were outputted from BPNN2.

Table S9. Network structure information for BPNN2.

Network structure information		
Input layer	1 neuron	1 Paranoid
Hidden layer 1	6 neurons	1 Paranoid
Output layer	5 neurons	
Network type	FANN_NETTYPE_LAYER	
Training function	FANN_TRAIN_RPROP	
Error function	FANN_ERRORFUNC_LINEAR	

Termination function	FANN_STOPFUNC_MSE			
Hidden layer excitation function	FANN_SIGMOID_SYMMETRIC			
Output layer excitation function	FANN_SIGMOID_SYMMETRIC			
**Network weight value**				
Arrangement	Wire number	Output point (n)	Input point (m)	Weight value (W)
**1**	0	0	2	12.0955
	1	1	2	0.848278
	2	0	3	1500
	3	1	3	-1500
	4	0	4	12.0557
	5	1	4	7.01856
	6	0	5	4.05591
	7	1	5	3.06074
	8	0	6	1500
	9	1	6	-1500
	10	0	7	1500
	11	1	7	-1500
**2**	12	2	9	1500
	13	3	9	1500
	14	4	9	1500
	15	5	9	1500
	16	6	9	1500
	17	7	9	1500
	18	8	9	1500
	19	2	10	-49.6721
	20	3	10	12.3906
	21	4	10	112.443
	22	5	10	10.5016
	23	6	10	11.6962
	24	7	10	29.2433
	25	8	10	-45.2951
	26	2	11	-89.1856
	27	3	11	21.8758
	28	4	11	16.5364
	29	5	11	51.1697
	30	6	11	50.6574
	31	7	11	11.0287
	32	8	11	-5.26973
	33	2	12	0.223775
	34	3	12	4.06612
	35	4	12	-42.8608
	36	5	12	56.7651
	37	6	12	9.40143
	38	7	12	5.37593
	39	8	12	-4.15748
	40	2	13	4.85797
	41	3	13	1.90535
	42	4	13	-1.49915
	43	5	13	-243.267
	44	6	13	25.8257
	45	7	13	26.6975
	46	8	13	-16.7816
**Input / output column coefficients for manual calculation**				
Listing	Minimum	Maximum		
$\Delta I/I_0$	0.329365	20.7542		

1.6 m s^{-1}	0	1
0.8 m s^{-1}	0	1
0.4 m s^{-1}	0	1
0.2 m s^{-1}	0	1
0.04 m s^{-1}	0	1
**Deviation statistics: mean variance**		
Listing	All rows	Calculation line
1.6 m s^{-1}	0	0
0.8 m s^{-1}	0	0
0.4 m s^{-1}	0	0
0.2 m s^{-1}	4.35609e-07	4.35609e-07
0.04 m s^{-1}	0	0

Table S10. The summary of mean square error (MSE), original value (OV), calculated value (CV), variance (Var) for BPNN2.

Input item	1.6 m s^{-1} (MSE = 0)			0.8 m s^{-1} (MSE = 0)			0.4 m s^{-1} (MSE = 0)			0.2 m s^{-1} (MSE = 4.35609e-07)			0.04 m s^{-1} (MSE = 0)		
$\Delta I/I_0$	OV	CV	Var	OV	CV	Var	OV	CV	Var	OV	CV	Var	OV	CV	Var
19.7165	1	1	0	0	0	0	0	0	0	0	0.000122	1.4884e-08	0	0	0
9.7288	0	0	0	1	1	0	0	0	0	0	0	0	0	0	0
4.8651	0	0	0	0	0	0	1	1	0	0	0.000262	6.8644e-08	0	0	0
2.0584	0	0	0	0	0	0	0	0	0	1	0.998962	1.07744e-06	0	0	0
0.3467	0	0	0	0	0	0	0	0	0	0	0.0010085	1.01707e-06	1	1	0

Table S11. The MATLAB code of the BPNN2.

MATLAB code

```
function [fO0, fO1, fO2, fO3, fO4] = MPredict(fI0)
    fI0 = (fI0 - (0.329365 + 20.7542105263157914 ) / 2.0) /(20.7542105263157914 - (0.329365 + 20.7542105263157914 ) / 2.0);
    fWei0 = 12.0955;
    fWei1 = 0.848278;
    fWei2 = 1500;
    fWei3 = -1500;
    fWei4 = 12.0557;
    fWei5 = 7.01856;
    fWei6 = 4.05591;
    fWei7 = 3.06074;
    fWei8 = 1500;
    fWei9 = -1500;
    fWei10 = 1500;
    fWei11 = -1500;
    fWei12 = 1500;
    fWei13 = 1500;
    fWei14 = 1500;
    fWei15 = 1500;
    fWei16 = 1500;
    fWei17 = 1500;
    fWei18 = 1500;
    fWei19 = -49.6721;
```

```
fWei20 = 12.3906;
fWei21 = 112.443;
fWei22 = 10.5016;
fWei23 = 11.6962;
fWei24 = 29.2433;
fWei25 = -45.2951;
fWei26 = -89.1856;
fWei27 = 21.8758;
fWei28 = 16.5364;
fWei29 = 51.1697;
fWei30 = 50.6574;
fWei31 = 11.0287;
fWei32 = -5.26973;
fWei33 = 0.223775;
fWei34 = 4.06612;
fWei35 = -42.8608;
fWei36 = 56.7651;
fWei37 = 9.40143;
fWei38 = 5.37593;
fWei39 = -4.15748;
fWei40 = 4.85797;
fWei41 = 1.90535;
fWei42 = -1.49915;
fWei43 = -243.267;
fWei44 = 25.8257;
fWei45 = 26.6975;
fWei46 = -16.7816;
f0 = fI0;
f1 = 1.0;
f2 = 0.0;
f3 = 0.0;
f4 = 0.0;
f5 = 0.0;
f6 = 0.0;
f7 = 0.0;
f2 = f2 + f0 * fWei0;
f2 = f2 + f1 * fWei1;
f2 = f2 * 0.5;
f2 = (2.0 / (1.0 + exp(-2.0 * f2))- 1.0);
f3 = f3 + f0 * fWei2;
f3 = f3 + f1 * fWei3;
f3 = f3 * 0.5;
f3 = (2.0 / (1.0 + exp(-2.0 * f3))- 1.0);
f4 = f4 + f0 * fWei4;
f4 = f4 + f1 * fWei5;
```

```
f4 = f4 * 0.5;
f4 = (2.0 / (1.0 + exp(-2.0 * f4))- 1.0);
f5 = f5 + f0 * fWei6;
f5 = f5 + f1 * fWei7;
f5 = f5 * 0.5;
f5 = (2.0 / (1.0 + exp(-2.0 * f5))- 1.0);
f6 = f6 + f0 * fWei8;
f6 = f6 + f1 * fWei9;
f6 = f6 * 0.5;
f6 = (2.0 / (1.0 + exp(-2.0 * f6))- 1.0);
f7 = f7 + f0 * fWei10;
f7 = f7 + f1 * fWei11;
f7 = f7 * 0.5;
f7 = (2.0 / (1.0 + exp(-2.0 * f7))- 1.0);
f8 = 1.0;
f9 = 0.0;
f10 = 0.0;
f11 = 0.0;
f12 = 0.0;
f13 = 0.0;
f9 = f9 + f2 * fWei12;
f9 = f9 + f3 * fWei13;
f9 = f9 + f4 * fWei14;
f9 = f9 + f5 * fWei15;
f9 = f9 + f6 * fWei16;
f9 = f9 + f7 * fWei17;
f9 = f9 + f8 * fWei18;
f9 = f9 * 0.5;
f9 = (2.0 / (1.0 + exp(-2.0 * f9))- 1.0);
f10 = f10 + f2 * fWei19;
f10 = f10 + f3 * fWei20;
f10 = f10 + f4 * fWei21;
f10 = f10 + f5 * fWei22;
f10 = f10 + f6 * fWei23;
f10 = f10 + f7 * fWei24;
f10 = f10 + f8 * fWei25;
f10 = f10 * 0.5;
f10 = (2.0 / (1.0 + exp(-2.0 * f10))- 1.0);
f11 = f11 + f2 * fWei26;
f11 = f11 + f3 * fWei27;
f11 = f11 + f4 * fWei28;
f11 = f11 + f5 * fWei29;
f11 = f11 + f6 * fWei30;
f11 = f11 + f7 * fWei31;
f11 = f11 + f8 * fWei32;
```

```
f11 = f11 * 0.5;
f11 = (2.0 / (1.0 + exp(-2.0 * f11))- 1.0);
f12 = f12 + f2 * fWei33;
f12 = f12 + f3 * fWei34;
f12 = f12 + f4 * fWei35;
f12 = f12 + f5 * fWei36;
f12 = f12 + f6 * fWei37;
f12 = f12 + f7 * fWei38;
f12 = f12 + f8 * fWei39;
f12 = f12 * 0.5;
f12 = (2.0 / (1.0 + exp(-2.0 * f12))- 1.0);
f13 = f13 + f2 * fWei40;
f13 = f13 + f3 * fWei41;
f13 = f13 + f4 * fWei42;
f13 = f13 + f5 * fWei43;
f13 = f13 + f6 * fWei44;
f13 = f13 + f7 * fWei45;
f13 = f13 + f8 * fWei46;
f13 = f13 * 0.5;
f13 = (2.0 / (1.0 + exp(-2.0 * f13))- 1.0);
fO0 = f9;
fO1 = f10;
fO2 = f11;
fO3 = f12;
fO4 = f13;
fO0 = fO0 * (1 - (0 + 1 ) / 2.0) + (0 + 1) / 2.0;
fO1 = fO1 * (1 - (0 + 1 ) / 2.0) + (0 + 1) / 2.0;
fO2 = fO2 * (1 - (0 + 1 ) / 2.0) + (0 + 1) / 2.0;
fO3 = fO3 * (1 - (0 + 1 ) / 2.0) + (0 + 1) / 2.0;
fO4 = fO4 * (1 - (0 + 1 ) / 2.0) + (0 + 1) / 2.0;
```

Table S12. Summary of input and output information during the training for BPNN3.

Input						Output		
$\Delta I/I_0$						Health	Help	Danger
1.29	4.09	1.22	0.55	6.15	1.09	1	0	0
1.02	2.41	0.45	3	0	0	0	1	0
7.21	1.41	2.4	6.93	6.51	0.61	0	0	1

In Table S12, all data is applied to train for the BPNN3. The relative fluorescence intensity variation ($\Delta I/I_0$) was inputted into BPNN3. The word information “Health”, “Help” and “Danger” was outputted from BPNN3.

Table S13. Network structure information for BPNN3.

Network structure information				
Input layer	6 neurons	1 Paranoid		
Hidden layer 1	6 neurons	1 Paranoid		
Output layer	3 neurons			
Network type	FANN_NETTYPE_LAYER			
Training function	FANN_TRAIN_RPROP			
Error function	FANN_ERRORFUNC_LINEAR			
Termination function	FANN_STOPFUNC_MSE			
Hidden layer excitation function	FANN_SIGMOID_SYMMETRIC			
Output layer excitation function	FANN_SIGMOID_SYMMETRIC			
**Network weight value**				
Arrangement	Wire number	Output point (n)	Input point (m)	Weight value (W)
	0	0	7	0.990607
	1	1	7	2.5062
	2	2	7	1.13958
	3	3	7	-0.865628
	4	4	7	1.78153
	5	5	7	3.69398
	6	6	7	0.0256118
	7	0	8	0.369185
	8	1	8	1.0428
	9	2	8	-0.0268788
	10	3	8	0.285833
	11	4	8	-0.20458
	12	5	8	0.709128
	13	6	8	-1.29165
	14	0	9	0.640174
	15	1	9	2.65348
	16	2	9	1.68889
	17	3	9	0.36047
	18	4	9	3.64407
	19	5	9	3.8209
**1**	20	6	9	1.29974
	21	0	10	0.410003
	22	1	10	-2.04943
	23	2	10	0.061342
	24	3	10	2.8452
	25	4	10	-1.65679
	26	5	10	-2.93893
	27	6	10	1.97533
	28	0	11	1.45487
	29	1	11	-1.80799
	30	2	11	1.05054
	31	3	11	1.74716
	32	4	11	0.84985
	33	5	11	-0.76205
	34	6	11	0.251295
	35	0	12	1.94277
	36	1	12	-4.0421
	37	2	12	0.898181
	38	3	12	1.87933
	39	4	12	0.593867
	40	5	12	-0.973093
	41	6	12	0.307517

	42	7	14	2.77971
	43	8	14	2.51394
	44	9	14	1.32892
	45	10	14	-5.51516
	46	11	14	-1.71964
	47	12	14	-2.83167
	48	13	14	-1.97963
	49	7	15	-5.74686
	50	8	15	1.76616
	51	9	15	-5.81717
**2**	52	10	15	1.37282
	53	11	15	-1.6528
	54	12	15	-1.78399
	55	13	15	-1.87731
	56	7	16	0.318815
	57	8	16	3.03715
	58	9	16	1.57651
	59	10	16	1.15153
	60	11	16	6.34048
	61	12	16	6.11457
	62	13	16	-0.472473

**Input / output column coefficients for manual calculation**		
Listing	Minimum	Maximum
	0.969	7.58947
	1.3395	4.30526
$\Delta I/I_0$	0.4275	2.52632
	0.5225	7.29474
	0	6.85263
	0	1.14737
Health	0	1
Help	0	1
Danger	0	1

**Deviation statistics: mean variance**		
Listing	All rows	Calculation line
Health	3.04667e-10	3.04667e-10
Help	3.24167e-11	3.24167e-11
Danger	1.04167e-11	1.04167e-11

Table S14. The summary of mean square error (MSE), original value (OV), calculated value (CV), variance (Var) for BPNN3.

Input item						Health (MSE = 3.04667e-10)			Help (MSE = 3.24167e-11)			Danger (MSE =1.04167e-11)		
$\Delta I/I_0$						OV	CV	Var	OV	CV	Var	OV	CV	Var
1.29	4.09	1.22	0.55	6.15	1.09	1	0.999989	1.21e-10	0	7e-06	4.9e-11	0	2.5e-06	6.25e-12
1.02	2.41	0.45	3	0	0	0	2.7e-05	7.29e-10	1	0.999994	3.6e-11	0	3e-06	9e-12
7.21	1.41	2.4	6.93	6.51	0.61	0	8e-06	6.4e-11	0	3.5e-06	1.225e-11	1	0.999996	1.6e-11
1.29	4.09	1.22	0.55	6.15	1.09	1	0.999989	1.21e-10	0	7e-06	4.9e-11	0	2.5e-06	6.25e-12
1.02	2.41	0.45	3	0	0	0	2.7e-05	7.29e-10	1	0.999994	3.6e-11	0	3e-06	9e-12

Table S15. The MATLAB code of the BPNN3.

**MATLAB code**
function [fO0, fO1, fO2] = MPredict(fI0, fI1, fI2, fI3, fI4, fI5)

```
fI0 = (fI0 - (0.969 + 7.5894736842105264 ) / 2.0) /(7.5894736842105264 - (0.969 + 7.5894736842105264 ) / 2.0);
fI1 = (fI1 - (1.3394999999999999 + 4.3052631578947365 ) / 2.0) /(4.3052631578947365 - (1.3394999999999999 + 4.3052631578947365 ) / 2.0);
fI2 = (fI2 - (0.4275 + 2.5263157894736841 ) / 2.0) /(2.5263157894736841 - (0.4275 + 2.5263157894736841 ) / 2.0);
fI3 = (fI3 - (0.5225 + 7.2947368421052632 ) / 2.0) /(7.2947368421052632 - (0.5225 + 7.2947368421052632 ) / 2.0);
fI4 = (fI4 - (0 + 6.8526315789473689 ) / 2.0) /(6.8526315789473689 - (0 + 6.8526315789473689 ) / 2.0);
fI5 = (fI5 - (0 + 1.1473684210526318 ) / 2.0) /(1.1473684210526318 - (0 + 1.1473684210526318 ) / 2.0);
fWei0 = 0.990607;
fWei1 = 2.5062;
fWei2 = 1.13958;
fWei3 = -0.865628;
fWei4 = 1.78153;
fWei5 = 3.69398;
fWei6 = 0.0256118;
fWei7 = 0.369185;
fWei8 = 1.0428;
fWei9 = -0.0268788;
fWei10 = 0.285833;
fWei11 = -0.20458;
fWei12 = 0.709128;
fWei13 = -1.29165;
fWei14 = 0.640174;
fWei15 = 2.65348;
fWei16 = 1.68889;
fWei17 = 0.36047;
fWei18 = 3.64407;
fWei19 = 3.8209;
fWei20 = 1.29974;
fWei21 = 0.410003;
fWei22 = -2.04943;
fWei23 = 0.061342;
fWei24 = 2.8452;
fWei25 = -1.65679;
fWei26 = -2.93893;
fWei27 = 1.97533;
fWei28 = 1.45487;
fWei29 = -1.80799;
fWei30 = 1.05054;
fWei31 = 1.74716;
fWei32 = 0.84985;
fWei33 = -0.76205;
fWei34 = 0.251295;
fWei35 = 1.94277;
fWei36 = -4.0421;
fWei37 = 0.898181;
fWei38 = 1.87933;
```

```
fWei39 = 0.593867;
fWei40 = -0.973093;
fWei41 = 0.307517;
fWei42 = 2.77971;
fWei43 = 2.51394;
fWei44 = 1.32892;
fWei45 = -5.51516;
fWei46 = -1.71964;
fWei47 = -2.83167;
fWei48 = -1.97963;
fWei49 = -5.74686;
fWei50 = 1.76616;
fWei51 = -5.81717;
fWei52 = 1.37282;
fWei53 = -1.6528;
fWei54 = -1.78399;
fWei55 = -1.87731;
fWei56 = 0.318815;
fWei57 = 3.03715;
fWei58 = 1.57651;
fWei59 = 1.15153;
fWei60 = 6.34048;
fWei61 = 6.11457;
fWei62 = -0.472473;
f0 = fI0;
f1 = fI1;
f2 = fI2;
f3 = fI3;
f4 = fI4;
f5 = fI5;
f6 = 1.0;
f7 = 0.0;
f8 = 0.0;
f9 = 0.0;
f10 = 0.0;
f11 = 0.0;
f12 = 0.0;
f7 = f7 + f0 * fWei0;
f7 = f7 + f1 * fWei1;
f7 = f7 + f2 * fWei2;
f7 = f7 + f3 * fWei3;
f7 = f7 + f4 * fWei4;
f7 = f7 + f5 * fWei5;
f7 = f7 + f6 * fWei6;
f7 = f7 * 0.5;
```

```
f7 = (2.0 / (1.0 + exp(-2.0 * f7))- 1.0);
f8 = f8 + f0 * fWei7;
f8 = f8 + f1 * fWei8;
f8 = f8 + f2 * fWei9;
f8 = f8 + f3 * fWei10;
f8 = f8 + f4 * fWei11;
f8 = f8 + f5 * fWei12;
f8 = f8 + f6 * fWei13;
f8 = f8 * 0.5;
f8 = (2.0 / (1.0 + exp(-2.0 * f8))- 1.0);
f9 = f9 + f0 * fWei14;
f9 = f9 + f1 * fWei15;
f9 = f9 + f2 * fWei16;
f9 = f9 + f3 * fWei17;
f9 = f9 + f4 * fWei18;
f9 = f9 + f5 * fWei19;
f9 = f9 + f6 * fWei20;
f9 = f9 * 0.5;
f9 = (2.0 / (1.0 + exp(-2.0 * f9))- 1.0);
f10 = f10 + f0 * fWei21;
f10 = f10 + f1 * fWei22;
f10 = f10 + f2 * fWei23;
f10 = f10 + f3 * fWei24;
f10 = f10 + f4 * fWei25;
f10 = f10 + f5 * fWei26;
f10 = f10 + f6 * fWei27;
f10 = f10 * 0.5;
f10 = (2.0 / (1.0 + exp(-2.0 * f10))- 1.0);
f11 = f11 + f0 * fWei28;
f11 = f11 + f1 * fWei29;
f11 = f11 + f2 * fWei30;
f11 = f11 + f3 * fWei31;
f11 = f11 + f4 * fWei32;
f11 = f11 + f5 * fWei33;
f11 = f11 + f6 * fWei34;
f11 = f11 * 0.5;
f11 = (2.0 / (1.0 + exp(-2.0 * f11))- 1.0);
f12 = f12 + f0 * fWei35;
f12 = f12 + f1 * fWei36;
f12 = f12 + f2 * fWei37;
f12 = f12 + f3 * fWei38;
f12 = f12 + f4 * fWei39;
f12 = f12 + f5 * fWei40;
f12 = f12 + f6 * fWei41;
f12 = f12 * 0.5;
```

```
f12 = (2.0 / (1.0 + exp(-2.0 * f12))- 1.0);
f13 = 1.0;
f14 = 0.0;
f15 = 0.0;
f16 = 0.0;
f14 = f14 + f7 * fWei42;
f14 = f14 + f8 * fWei43;
f14 = f14 + f9 * fWei44;
f14 = f14 + f10 * fWei45;
f14 = f14 + f11 * fWei46;
f14 = f14 + f12 * fWei47;
f14 = f14 + f13 * fWei48;
f14 = f14 * 0.5;
f14 = (2.0 / (1.0 + exp(-2.0 * f14))- 1.0);
f15 = f15 + f7 * fWei49;
f15 = f15 + f8 * fWei50;
f15 = f15 + f9 * fWei51;
f15 = f15 + f10 * fWei52;
f15 = f15 + f11 * fWei53;
f15 = f15 + f12 * fWei54;
f15 = f15 + f13 * fWei55;
f15 = f15 * 0.5;
f15 = (2.0 / (1.0 + exp(-2.0 * f15))- 1.0);
f16 = f16 + f7 * fWei56;
f16 = f16 + f8 * fWei57;
f16 = f16 + f9 * fWei58;
f16 = f16 + f10 * fWei59;
f16 = f16 + f11 * fWei60;
f16 = f16 + f12 * fWei61;
f16 = f16 + f13 * fWei62;
f16 = f16 * 0.5;
f16 = (2.0 / (1.0 + exp(-2.0 * f16))- 1.0);
fO0 = f14;
fO1 = f15;
fO2 = f16;
fO0 = fO0 * (1 - (0 + 1 ) / 2.0) + (0 + 1) / 2.0;
fO1 = fO1 * (1 - (0 + 1 ) / 2.0) + (0 + 1) / 2.0;
fO2 = fO2 * (1 - (0 + 1 ) / 2.0) + (0 + 1) / 2.0;
```

Table S16. Summary of input and output information during the training for BPNN4.

Input		Output			
Order	$\Delta I/I_0$	H	E	L	P

8	3.1	1	0	0	0
5	0.7	0	1	0	0
12	1.24	0	0	1	0
16	2.65	0	0	0	1

In Table S16, all data is applied to train for the BPNN4. The relative fluorescence intensity variation ($\Delta I/I_0$) and the order of letters were inputted into BPNN4. The word information "H, E, L, P" was outputted from BPNN4.

Table S17. Network structure information for BPNN4.

Network structure information				
Input layer	2 neurons	1 Paranoid		
Hidden layer 1	6 neurons	1 Paranoid		
Output layer	4 neurons			
Network type	FANN_NETTYPE_LAYER			
Training function	FANN_TRAIN_RPROP			
Error function	FANN_ERRORFUNC_LINEAR			
Termination function	FANN_STOPFUNC_MSE			
Hidden layer excitation function	FANN_SIGMOID_SYMMETRIC			
Output layer excitation function	FANN_SIGMOID_SYMMETRIC			
**Network weight value**				
Arrangement	Wire number	Output point (n)	Input point (m)	Weight value (W)
	0	0	3	-0.776266
	1	1	3	3.13646
	2	2	3	-0.0182008
	3	0	4	22.0693
	4	1	4	0.307388
	5	2	4	1.7251
	6	0	5	1.59338
	7	1	5	13.0292
**1**	8	2	5	-0.63666
	9	0	6	0.361406
	10	1	6	-0.791783
	11	2	6	-5.32844
	12	0	7	-3.1102
	13	1	7	3.4226
	14	2	7	1.26298
	15	0	8	3.72088
	16	1	8	5.33203
	17	2	8	-1.40001
	18	3	10	17.3564
	19	4	10	-2.75608
	20	5	10	-5.05949
	21	6	10	5.23601
	22	7	10	10.5437
**2**	23	8	10	-0.808921
	24	9	10	-8.80555
	25	3	11	-2.63722
	26	4	11	-19.1177
	27	5	11	-16.6313
	28	6	11	19.1206
	29	7	11	1.10796

30	8	11	1.75138
31	9	11	-0.49484
32	3	12	-1.12542
33	4	12	1.15989
34	5	12	-0.697303
35	6	12	1.14895
36	7	12	-16.5043
37	8	12	-1.8436
38	9	12	-3.2518
39	3	13	-2.31432
40	4	13	0.534171
41	5	13	-1.42494
42	6	13	3.13826
43	7	13	-34.9407
44	8	13	28.6875
45	9	13	-5.63246

Input / output column coefficients for manual calculation

Listing	Minimum	Maximum
Order	4.75	16.8421
$\Delta I/I_0$	0.665	3.26316
H	0	1
E	0	1
L	0	1
P	0	1

Deviation statistics: mean variance

Listing	All rows	Calculation line
H	1.52331e-09	1.52331e-09
E	0	0
L	6.61156e-09	6.61156e-09
P	1.345e-10	1.345e-10

Table S18. The summary of mean square error (MSE), original value (OV), calculated value (CV), variance (Var) for BPNN4.

Input item		Health (MSE = 1.52331e-09)			Health (MSE = 0)			Health (MSE = 6.61156e-09)			Health (MSE = 1.345e-10)		
Order	$\Delta I/I_0$	OV	CV	Var	OV	CV	Var	OV	CV	Var	OV	CV	Var
8	3.1	1	0.999922	6.084e-09	0	0	0	0	0	0	0	0	0
5	0.7	0	5e-07	2.5e-13	1	1	0	0	0.0001075	1.15562e-08	0	0	0
12	1.24	0	0	0	0	0	0	1	0.999907	8.649e-09	0	3e-06	9e-12
16	2.65	0	3e-06	9e-12	0	0	0	0	7.9e-05	6.241e-09	1	0.999977	5.29e-10

Table S19. The MATLAB code of the BPNN4.

MATLAB code

```
function [fO0, fO1, fO2, fO3] = MPredict(fI0, fI1)
    fI0 = (fI0 - (4.75 + 16.8421052631578938 ) / 2.0) /(16.8421052631578938 - (4.75 + 16.8421052631578938 ) / 2.0);
    fI1 = (fI1 - (0.6649999999999999 + 3.2631578947368425 ) / 2.0) /(3.2631578947368425 - (0.6649999999999999 + 3.2631578947368425 ) / 2.0);
    fWei0 = -0.776266;
    fWei1 = 3.13646;
    fWei2 = -0.0182008;
    fWei3 = 22.0693;
    fWei4 = 0.307388;
```

```
fWei5 = 1.7251;
fWei6 = 1.59338;
fWei7 = 13.0292;
fWei8 = -0.63666;
fWei9 = 0.361406;
fWei10 = -0.791783;
fWei11 = -5.32844;
fWei12 = -3.1102;
fWei13 = 3.4226;
fWei14 = 1.26298;
fWei15 = 3.72088;
fWei16 = 5.33203;
fWei17 = -1.40001;
fWei18 = 17.3564;
fWei19 = -2.75608;
fWei20 = -5.05949;
fWei21 = 5.23601;
fWei22 = 10.5437;
fWei23 = -0.808921;
fWei24 = -8.80555;
fWei25 = -2.63722;
fWei26 = -19.1177;
fWei27 = -16.6313;
fWei28 = 19.1206;
fWei29 = 1.10796;
fWei30 = 1.75138;
fWei31 = -0.49484;
fWei32 = -1.12542;
fWei33 = 1.15989;
fWei34 = -0.697303;
fWei35 = 1.14895;
fWei36 = -16.5043;
fWei37 = -1.8436;
fWei38 = -3.2518;
fWei39 = -2.31432;
fWei40 = 0.534171;
fWei41 = -1.42494;
fWei42 = 3.13826;
fWei43 = -34.9407;
fWei44 = 28.6875;
fWei45 = -5.63246;
f0 = fI0;
f1 = fI1;
f2 = 1.0;
f3 = 0.0;
```

```
f4 = 0.0;
f5 = 0.0;
f6 = 0.0;
f7 = 0.0;
f8 = 0.0;
f3 = f3 + f0 * fWei0;
f3 = f3 + f1 * fWei1;
f3 = f3 + f2 * fWei2;
f3 = f3 * 0.5;
f3 = (2.0 / (1.0 + exp(-2.0 * f3))- 1.0);
f4 = f4 + f0 * fWei3;
f4 = f4 + f1 * fWei4;
f4 = f4 + f2 * fWei5;
f4 = f4 * 0.5;
f4 = (2.0 / (1.0 + exp(-2.0 * f4))- 1.0);
f5 = f5 + f0 * fWei6;
f5 = f5 + f1 * fWei7;
f5 = f5 + f2 * fWei8;
f5 = f5 * 0.5;
f5 = (2.0 / (1.0 + exp(-2.0 * f5))- 1.0);
f6 = f6 + f0 * fWei9;
f6 = f6 + f1 * fWei10;
f6 = f6 + f2 * fWei11;
f6 = f6 * 0.5;
f6 = (2.0 / (1.0 + exp(-2.0 * f6))- 1.0);
f7 = f7 + f0 * fWei12;
f7 = f7 + f1 * fWei13;
f7 = f7 + f2 * fWei14;
f7 = f7 * 0.5;
f7 = (2.0 / (1.0 + exp(-2.0 * f7))- 1.0);
f8 = f8 + f0 * fWei15;
f8 = f8 + f1 * fWei16;
f8 = f8 + f2 * fWei17;
f8 = f8 * 0.5;
f8 = (2.0 / (1.0 + exp(-2.0 * f8))- 1.0);
f9 = 1.0;
f10 = 0.0;
f11 = 0.0;
f12 = 0.0;
f13 = 0.0;
f10 = f10 + f3 * fWei18;
f10 = f10 + f4 * fWei19;
f10 = f10 + f5 * fWei20;
f10 = f10 + f6 * fWei21;
f10 = f10 + f7 * fWei22;
```

```
f10 = f10 + f8 * fWei23;
f10 = f10 + f9 * fWei24;
f10 = f10 * 0.5;
f10 = (2.0 / (1.0 + exp(-2.0 * f10))- 1.0);
f11 = f11 + f3 * fWei25;
f11 = f11 + f4 * fWei26;
f11 = f11 + f5 * fWei27;
f11 = f11 + f6 * fWei28;
f11 = f11 + f7 * fWei29;
f11 = f11 + f8 * fWei30;
f11 = f11 + f9 * fWei31;
f11 = f11 * 0.5;
f11 = (2.0 / (1.0 + exp(-2.0 * f11))- 1.0);
f12 = f12 + f3 * fWei32;
f12 = f12 + f4 * fWei33;
f12 = f12 + f5 * fWei34;
f12 = f12 + f6 * fWei35;
f12 = f12 + f7 * fWei36;
f12 = f12 + f8 * fWei37;
f12 = f12 + f9 * fWei38;
f12 = f12 * 0.5;
f12 = (2.0 / (1.0 + exp(-2.0 * f12))- 1.0);
f13 = f13 + f3 * fWei39;
f13 = f13 + f4 * fWei40;
f13 = f13 + f5 * fWei41;
f13 = f13 + f6 * fWei42;
f13 = f13 + f7 * fWei43;
f13 = f13 + f8 * fWei44;
f13 = f13 + f9 * fWei45;
f13 = f13 * 0.5;
f13 = (2.0 / (1.0 + exp(-2.0 * f13))- 1.0);
fO0 = f10;
fO1 = f11;
fO2 = f12;
fO3 = f13;
fO0 = fO0 * (1 - (0 + 1 ) / 2.0) + (0 + 1) / 2.0;
fO1 = fO1 * (1 - (0 + 1 ) / 2.0) + (0 + 1) / 2.0;
fO2 = fO2 * (1 - (0 + 1 ) / 2.0) + (0 + 1) / 2.0;
fO3 = fO3 * (1 - (0 + 1 ) / 2.0) + (0 + 1) / 2.0;
```

Reference

(1) Wang, H.; Li, S.; Wang, Y.; Wang, H.; Shen, X.; Zhang, M.; Lu, H.; He, M.; Zhang, Y. Bioinspired Fluffy Fabric with In Situ Grown Carbon Nanotubes for Ultrasensitive Wearable Airflow Sensor. *Adv. Mater.* **2020**, *32*, 1908214.

(2) Xu, Z.; Wu, K.; Zhang, S.; Meng, Y.; Li, H.; Li, L. Highly sensitive airflow sensors with an ultrathin reduced graphene oxide film inspired by gas exfoliation of graphite oxide. *Mater. Horiz.* **2017**, *4*, 383-388.

(3) Park, J.; Lee, Y.; Hong, J.; Ha, M.; Jung, Y.-D.; Lim, H.; Kim, S. Y.; Ko, H. Giant Tunneling Piezoresistance of Composite Elastomers with Interlocked Microdome Arrays for Ultrasensitive and Multimodal Electronic Skins. *ACS Nano* **2014**, *8*, 4689-4697.

(4) Zhao, S.; Zhu, R. Electronic Skin with Multifunction Sensors Based on Thermosensation. *Adv. Mater.* **2017**, *29*, 1606151.

(5) Sadeghi, M. M.; Peterson, R. L.; Najafi, K. Air flow sensing using micro-wire-bonded hair-like hot-wire anemometry. *J. Micromech. Microeng.* **2013**, *23*, 085017.

(6) Bian, Y.; Liu, R.; Hui, S. Fabrication of a polyvinylidene difluoride fiber with a metal core and its application as directional air flow sensor. *Funct. Mater. Lett.* **2016**, *09*, 1650001.

(7) Xing, F.; Yang, Y.; Shen, J.; Jiang, W.; Liu, Z.; Zhu, S.; Yuan, X. Ultra-high sensitivity, multi-parameter monitoring of dynamical gas parameters using a reduced graphene oxide microcavity. *Sens. Actuators B Chem.* **2016**, *235*, 474-480.

(8) Jiang, Q.; Li, R.; Wang, F.; Shi, X.; Chen, F.; Huang, Y.; Wang, B.; Zhang, W.; Wu, X.; Wei, F.; Zhang, R. Ultrasensitive Airflow Sensors Based on Suspended Carbon Nanotube Networks. *Adv. Mater.* **2022**, *34*, 2107062.

(9) Zhou, W.; Xiao, P.; Liang, Y.; Wang, Q.; Liu, D.; Yang, Q.; Chen, J.; Nie, Y.; Kuo, S.-W.; Chen, T. Bionic Adaptive Thin-Membranes Sensory System Based on Microspring Effect for High-Sensitive Airflow Perception and Noncontact Manipulation. *Adv. Funct. Mater.* **2021**, *31*, 2105323.

(10) Wang, P.; Wang, M.; Zhu, J.; Wang, Y.; Gao, J.; Gao, C.; Gao, Q. Surface engineering via self-assembly on PEDOT: PSS fibers: Biomimetic fluff-like morphology and sensing application. *Chem. Eng. J.* **2021**, *425*, 131551.

(11) Luo, J.; Ji, N.; Zhang, W.; Ge, P.; Liu, Y.; Sun, J.; Wang, J.; Zhuo, Q.; Qin, C.; Dai, L. Ultrasensitive airflow sensor prepared by electrostatic flocking for sound recognition and motion monitoring. *Mater. Horiz.* **2022**, *9*, 1503-1512.

(12) Zhou, W.; Xiao, P.; Zhang, C.; Yang, Q.; Chen, T. Dynamic competitive strains enabled self-supporting Janus nanostructured films for high-performance airflow perception. *Mater. Horiz.* **2023**, *10*, 1264-1273.

(13) Liu, Z.; Zhang, S.; Wu, Z.; Mu, E.; Wei, H.; Liu, Y.; Shi, H.; Hu, Z. High-performance integrated chip-level thermoelectric device for power generation and microflow detection. *Nano Energy* **2023**, *114*, 108611.

(14) Huang, L.; Liu, Y.; Li, G.; Song, Y.; Su, J.; Cheng, L.; Guo, W.; Zhao, G.; Shen, H.; Yan, Z.; Tang, B. Z.; Ye, R. Ultrasensitive, Fast-Responsive, Directional Airflow Sensing by Bioinspired Suspended Graphene Fibers. *Nano Lett.* **2023**, *23*, 597-605.

(15) Huang, S.; Zhang, B.; Lin, Y.; Lee, C.-S.; Zhang, X. Compact Biomimetic Hair Sensors Based on Single Silicon Nanowires for Ultrafast and Highly-Sensitive Airflow Detection. *Nano Lett.* **2021**, *21*, 4684-4691.

(16) Bian, Y.; Zhang, Y.; Xia, X. Design and Fabrication of a Multi-electrode Metal-core Piezoelectric Fiber and Its Application as an Airflow Sensor. *J. Bionic Eng.* **2016**, *13*, 416-425.

(17) Nie, P.; Wang, R.; Xu, X.; Cheng, Y.; Wang, X.; Shi, L.; Sun, J. High-Performance Piezoresistive Electronic Skin with Bionic Hierarchical Microstructure and Microcracks. *ACS Appl. Mater. Interfaces* **2017**, *9*, 14911-14919.

(18) Liu, Y.; Li, E.; Wang, X.; Chen, Q.; Zhou, Y.; Hu, Y.; Chen, G.; Chen, H.; Guo, T. Self-powered artificial auditory pathway for intelligent neuromorphic computing and sound detection. *Nano Energy* **2020**, *78*, 105403.

(19) Yu, Z.; Zhang, Y.; Wang, Y.; Zheng, J.; Fu, Y.; Chen, D.; Wang, G.; Cui, J.; Yu, S.; Zheng, L.; Zhou, H.; Li, D. Integrated piezo-tribo hybrid acoustic-driven nanogenerator based on porous MWCNTs/PVDF-TrFE aerogel bulk with embedded PDMS tympanum structure for broadband sound energy harvesting. *Nano Energy* **2022**, *97*, 107205.

(20) Wang, H. S.; Hong, S. K.; Han, J. H.; Jung, Y. H.; Jeong, H. K.; Im, T. H.; Jeong, C. K.; Lee, B.-Y.; Kim, G.; Yoo, C. D.; Lee, K. J. Biomimetic and flexible piezoelectric mobile acoustic sensors with multiresonant ultrathin structures for machine learning biometrics. *Sci. Adv.* **2021**, *7*, eabe5683.

(21) Lin, Z.; Zhang, G.; Xiao, X.; Au, C.; Zhou, Y.; Sun, C.; Zhou, Z.; Yan, R.; Fan, E.; Si, S.; Weng, L.; Mathur, S.; Yang, J.; Chen, J. A Personalized Acoustic Interface for Wearable Human–Machine Interaction. *Adv. Funct. Mater.* **2022**, *32*, 2109430.

(22) Gong, S.; Yap, L. W.; Zhu, Y.; Zhu, B.; Wang, Y.; Ling, Y.; Zhao, Y.; An, T.; Lu, Y.; Cheng, W. A Soft Resistive Acoustic Sensor Based on Suspended Standing Nanowire Membranes with Point Crack Design. *Adv. Funct. Mater.* **2020**, *30*, 1910717.

(23) Lee, S.; Kim, J.; Roh, H.; Kim, W.; Chung, S.; Moon, W.; Cho, K. A High-Fidelity Skin-Attachable Acoustic Sensor for Realizing Auditory Electronic Skin. *Adv. Mater.* **2022**, *34*, 2109545.

(24) Xiao, Q.; Ma, B.; Wang, S.-Y.; Li, X.-Y.; Yan, F.; Wang, Q.; Zhang, H.-L. Utilizing Topological Insulator Two-Dimensional Bismuth for Ultrasensitive Acoustic Detection. *Small* **2023**, *19*, 2303608.

(25) Wang, W.; Stipp, P. N.; Ouaras, K.; Fathi, S.; Huang, Y. Y. S. Broad Bandwidth, Self-Powered Acoustic Sensor Created by Dynamic Near-Field Electrospinning of Suspended, Transparent Piezoelectric Nanofiber Mesh. *Small* **2020**, *16*, 2000581.

(26) Lang, C.; Fang, J.; Shao, H.; Ding, X.; Lin, T. High-sensitivity acoustic sensors from nanofibre webs. *Nat. Commun.* **2016**, *7*, 11108.

(27) Yang, J.; Chen, J.; Liu, Y.; Yang, W.; Su, Y.; Wang, Z. L. Triboelectrification-Based Organic Film Nanogenerator for Acoustic Energy Harvesting and Self-Powered Active Acoustic Sensing. *ACS Nano* **2014**, *8*, 2649-2657.

(28) Park, J.; Kang, D.-h.; Chae, H.; Ghosh, S. K.; Jeong, C.; Park, Y.; Cho, S.; Lee, Y.; Kim, J.; Ko, Y.; Kim, J. J.; Ko, H. Frequency-selective acoustic and haptic smart skin for dual-mode dynamic/static human-machine interface. *Sci. Adv.* **2022**, *8*, eabj9220.

(29) Han, J. H.; Kwak, J.-H.; Joe, D. J.; Hong, S. K.; Wang, H. S.; Park, J. H.; Hur, S.; Lee, K. J. Basilar membrane-inspired self-powered acoustic sensor enabled by highly sensitive multi tunable frequency band. *Nano Energy* **2018**, *53*, 198-205.
